# A Hydrazone Ligand for Iridium-Catalyzed C–H Borylation: Enhanced Reactivity and Selectivity for Fluorinated Arenes

**DOI:** 10.1021/acs.organomet.4c00174

**Published:** 2024-05-20

**Authors:** Christopher D. Peruzzi, Susanne L. Miller, Jonathan E. Dannatt, Behnaz Ghaffari, Robert E. Maleczka, Milton R. Smith

**Affiliations:** † Department of Chemistry, Michigan State University, 578 South Shaw Lane, East Lansing, Michigan 48824, United States; ‡ Department of Chemistry, University of Dallas, 1845 East Northgate Drive, Irving, Texas 75062, United States

## Abstract

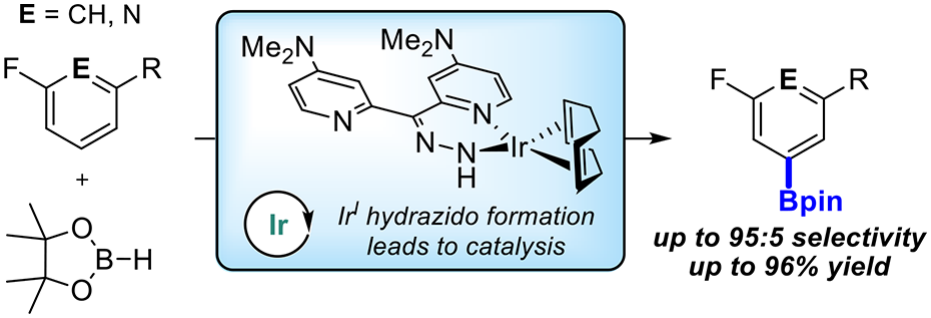

Ir-catalyzed C–H borylations of fluorinated and cyanated arenes with high *meta*-to-F/CN are described. Use of a dipyridyl hydrazone framework as the ancillary ligand and pinacolborane (HBpin) as the functionalizing reagent generates catalysts that are significantly more active *and* selective than 4,4′-di-*tert*-butyl-2,2′-bipyridine (dtbpy) for both electron-deficient and electron-rich substrates. Investigation of the ligand framework resulted in the observation of formal *N*-borylation of the hydrazone by HBpin, as evidenced by NMR spectroscopy and X-ray crystallography. Subsequent stoichiometric reactions of this adduct with an iridium precatalyst revealed the formation of an unusual Ir^I^ hydrazido. Isolation and use of this hydrazido reproduce the selectivity of *insitu* generated catalysts, suggesting that it leads to formation of the active species.

Ir-catalyzed C–H borylation (CHB) has become a ubiquitous, state of the art method for the direct formation of both alkyl and aryl boronic esters. Traditionally these reactions are sterically directed; however, many elegant catalysts have been designed to direct the C–H functionalization. Ortho-selectivity has been achieved using chelate,^1^ relay-directed,^2−4^ and outer-sphere interactions.^5^*Meta*- and *para*-selective borylations, though more difficult, have recently been realized through noncovalent interactions such as hydrogen bonding^6,7^ and electrostatic interactions.^8−10^ There are few systems capable, however, of achieving high selectivity in the direct borylation of fluoroarenes. The highly selective reactions are limited to cases where oxidative addition is reversible (“*ortho* fluorine effect”),^11−13^ directing groups are installed onto the substrate,^1,14^ or borylation–deborylation strategies,^15^ and the majority are *ortho*-selective.^16−19^ With the prominence of fluorine in pharmaceuticals^20^ and medicinal chemistry,^21^ developing C–H functionalizations with selectivities complementary to the existing methods is important.

The major challenges with site selectivity arise from the intrinsic properties associated with fluorine. Fluorine is only 20% larger than hydrogen,^22^ causing poor steric discrimination in the context of Ir-catalyzed CHBs,^23−25^ and is nonpolarizable,^22^ preventing strong electrostatic interactions to guide selectivity. Furthermore, experimental work from Jones, Perutz, and co-workers^12^ in addition to subsequent computational studies from Eisenstein demonstrated that across many transition metal–fluoroaryl complexes, the metal–carbon bond strength increases with increasing *ortho* fluorine substituents.^11,26^ Their findings suggest that, generally, regioselectivity for C–H activation is thermodynamically favored at sites *proximal* to F. Prior work from our group^27,28^ also has shown that, in agreement with increased metal–carbon bond strengths, the more acidic C–H bonds are more reactive. Thus, an electronically enhanced selectivity for borylation *ortho*-to-F should be found. The clash of the electronic and thermodynamic preference for *ortho*-to-F selectivity with the steric selectivity of CHBs results in, typically, poor regioselectivity for the CHB of fluoroarenes when utilizing Ir without the use of blocking groups or directing effects.

Thermodynamically, there is a small difference in the bond dissociation energies of the C–H bonds in fluorobenzene (<2.5 kcal mol^–1^).^28^ To achieve kinetic control, the barrier that leads to the thermodynamic product must be at least 2.5 kcal mol^–1^ (at 298 K) higher than the barrier leading to the kinetic product. Moreover, the reaction must be run under conditions in which equilibrium is not reached. Toward this aim, several CHB systems (Scheme 1) have been developed for their selectivity in nondirected functionalizations of C–H bonds. Recent work by the Chirik group (Scheme 1B) demonstrated CHBs with an electron-deficient Co catalyst bearing a terpyridine ligand enables slow C–H cleavage, affording up to 99:1 *meta*-to-F site selectivities.^29^ This is distinct from their [(^*i*Pr^PNP)Co] system,^29^ where high *ortho*-to-F selectivity is observed. The Driess group also reported a sterically encumbered Co catalyst generated from a pyridine bis-silylene ligand framework that provides high selectivities for meta functionalizations.^30^ Notably, these systems utilizing earth-abundant cobalt are amenable to only activated, electron-deficient arenes. Furthermore, the cobalt-based systems do not tolerate heavier halogens due to more favorable C–X cleavage (X = Cl, Br, I).

**Scheme 1:**
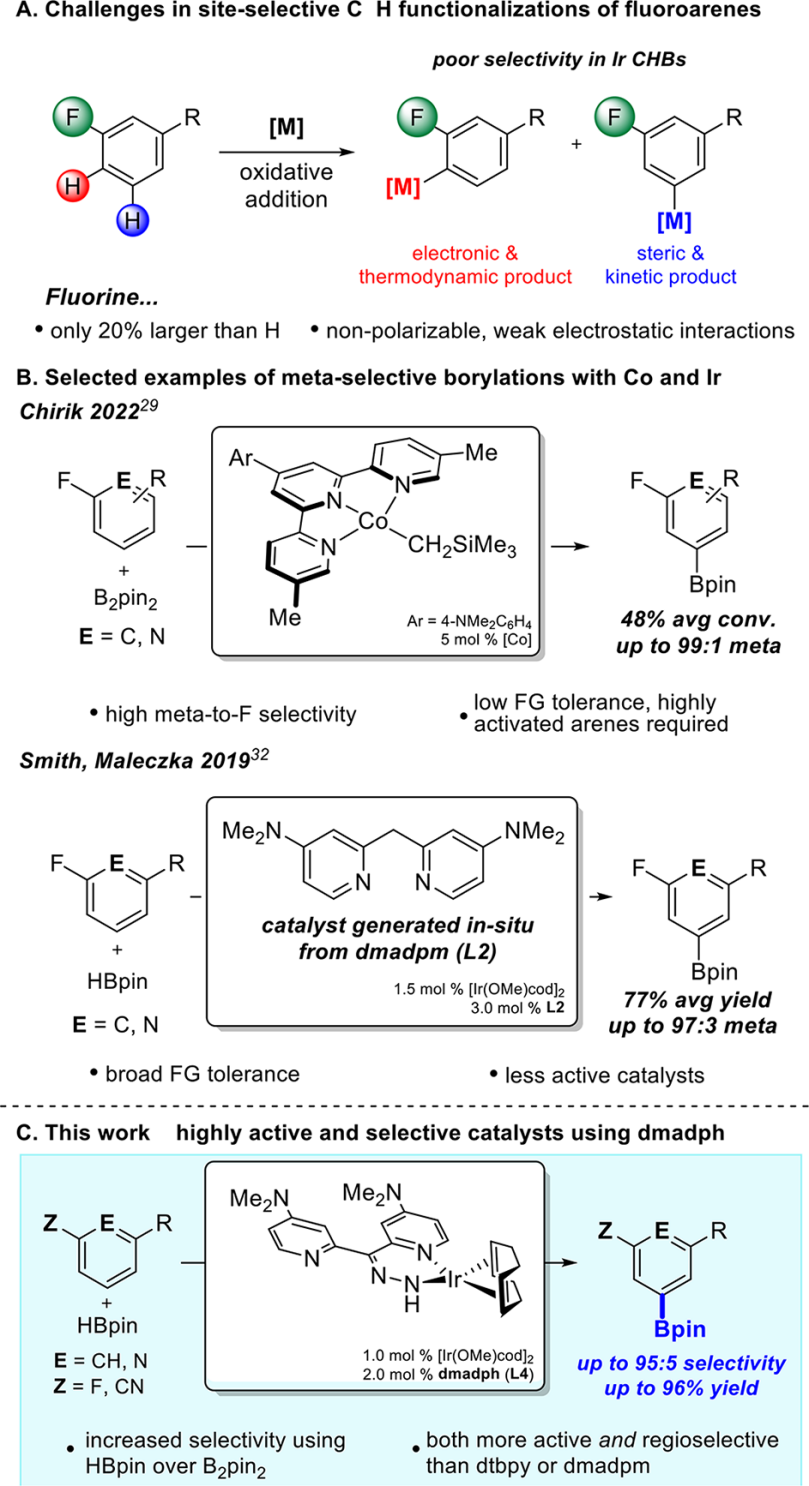
Challenges in Selective Fluoroarene Functionalizations

A well-known advantage of iridium catalysis is high functional group tolerance, but selective activation *meta* or *para* to fluorine via iridium catalysis is underdeveloped.^15,31^ Currently, high *ortho*-to-F selectivity can be achieved with an iridium-terpyridine catalyst that was developed by Ilies.^18^ The only system with high *meta*-to-F selectivity was developed by our group, utilizing **L2** (Scheme 1B) as the ligand.^32^ However, catalysts generated from **L2** are considerably less active than traditional bipyridines or phenanthrolines, requiring at least twice the reaction time for comparable conversions. Thus, we desired to generate a catalyst (Scheme 1C) that achieved both high *meta* or *para* to fluorine selectivity and retained activity on the order of iridium catalysts generated from bipyridines such as dtbpy (**L3**).

Inspired by prior demonstrations of hydrazone-based ligands in Ir-catalyzed CHBs^33,34^ and our prior studies of **L1** and **L2**, **L4** was designed to achieve this goal. Catalysts generated by **L4** were much more reactive than the dipyridylmethane-type ligands (**L1**, **L2**) and on par with those generated by dtbpy (**L3**). Solvent choice proved to be vital to improving *meta*-to-F selectivity, as using a nonpolar solvent (Scheme 2, entries 6 and 7) greatly diminished selectivity. Additionally, there is an effect of temperature on the observed selectivity, with lower temperatures improving the *meta*-to-F selectivity, consistent with kinetic control. This effect is distinct from Ir/bipyridine catalyzed CHBs, where temperature marginally impacts the regioselectivity.^35^ Though this effect was observed, 40 °C was the optimal temperature (Scheme 2, entry 8) for high activity while maintaining improved selectivity.

**Scheme 2:**
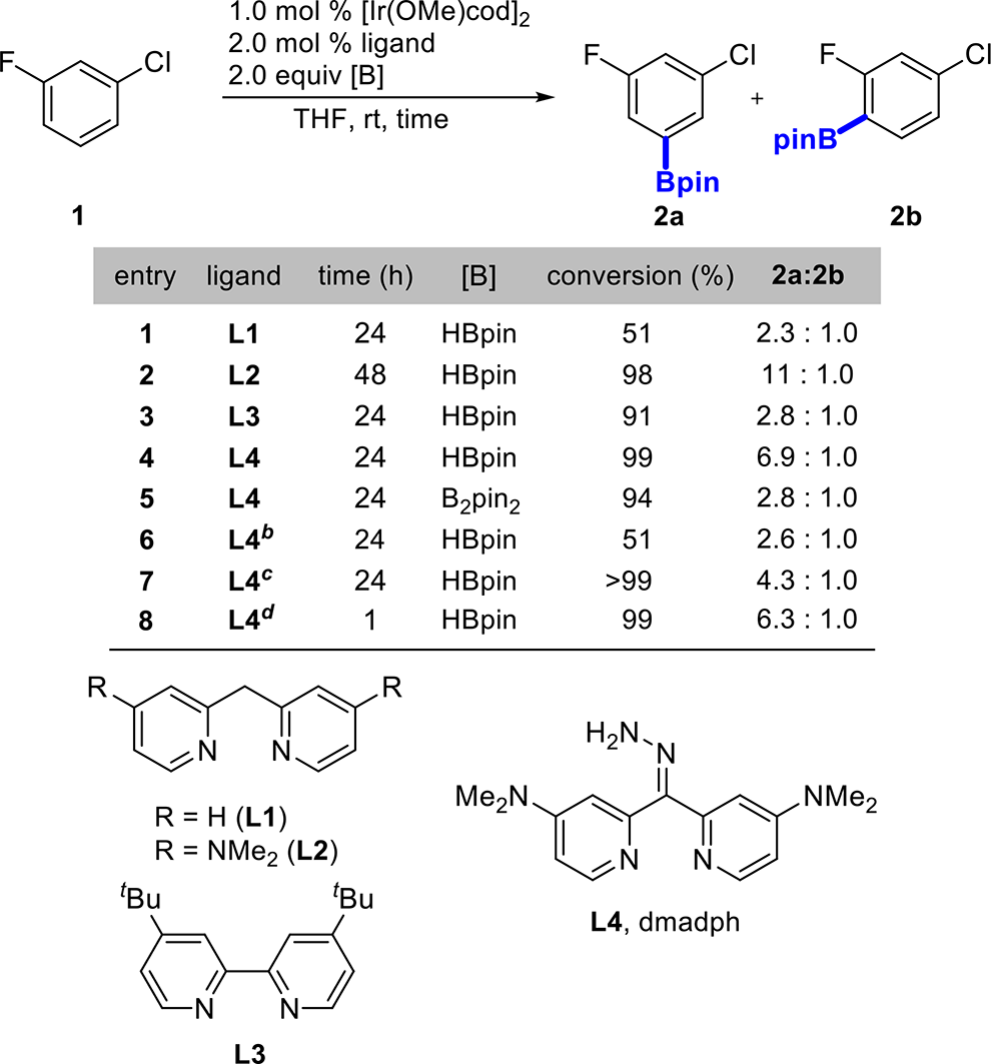
Optimization of Borylation Conditions Reaction conditions: fluoroarene (1.0 mmol), HBpin (2.0 mmol) or B_2_pin_2_ (1.0 mmol), [Ir(OMe)cod]_2_ (1.0 mol %), ligand (2.0 mol %), solvent (2.0 mL). *n*-Hexane used as solvent. CH_2_Cl_2_ used as solvent. Reaction run at 40 °C.

To demonstrate the advantages of using **L4** over dtbpy, we examined 1,3-disubstituted fluorinated arenes as shown in Table 1. Catalysts generated by **L4** afforded improved activity and selectivity for all of the 1,3-disubstituted arenes examined. Borylations of electron-poor substrates **4a**–**f** were essentially complete within 2 h, with kinetic selectivities of up to 18.0:1.0 and high yields. Notably, activated fluoroarenes containing heavier halogens (**4e**,**f**) were significantly less reactive when dtbpy was used as the ancillary ligand. Electron-rich substrates **4i**–**k** still required longer reaction times; however, a nearly 3-fold improvement in both conversion to products and selectivities were found with **L4**. The borylation of fluorobenzene (**4l**) shows improved site selectivity without the influence of other functional groups. We also wanted to examine **5** as a nonfluorinated substrate that typically requires elevated temperatures, prolonged reaction times, and a more reactive borylating reagent (B_2_pin_2_)^23,35^ to achieve good conversion. Under much milder conditions, **L4** achieves 72% conversion in 24 h, whereas dtbpy only reaches 18% conversion, demonstrating the superior activity of the catalysts generated.

**Table 1:**
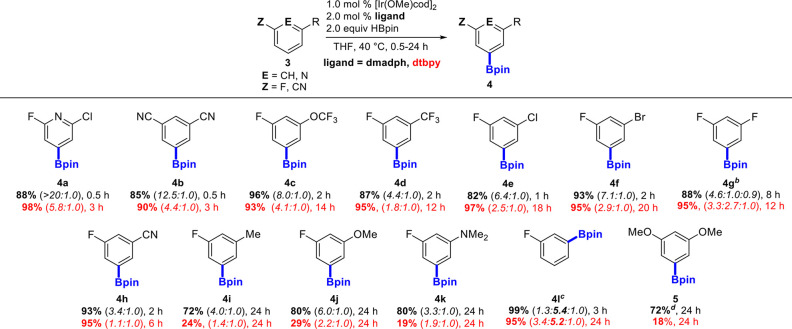
Meta-Selective C–H Borylations of 1,3-disubstituted Fluorinated and Cyanated Arenes^a^

a Reaction conditions: fluoroarene (**3**, 1.0 mmol), HBpin (2.0 mmol), [Ir(OMe)cod]_2_ (1.0 mol %), and **ligand** (2.0 mol %) in THF (2.5 mL), 40 °C, 0.5–24 h. Isolated yields are reported after column chromatography for dmadph, and selectivities are from crude reaction mixtures. Percent conversions found from ^19^F NMR are reported for dtbpy. Numbers in parentheses correspond to the ratio of *meta*:*ortho* to F borylated isomers.

b Ratio of 5:2,5:4 borylated isomers given in parentheses.

c 5 equiv of fluorobenzene was used to suppress diborylation. Ratio of *ortho*:*meta*:*para* to F borylated isomers given in parentheses.

d Reaction run at 65 °C.

In trying to rationalize the greatly improved selectivity when using **L4**, we considered the ligand framework and potential structural changes or reactions that could occur during catalysis.^36^ Previous work demonstrates that N–H and O–H sites are rapidly *N*- and *O*-borylated in CHB reactions catalyzed by an Ir species with B_2_pin_2_ or pinacolborane.^6,37,38^ As shown in Scheme 3a, the hydrazone is rapidly *N*-borylated in MeCN (without Ir) forming hydrazone–boronate adduct **6**. A sharp singlet was observed in the ^11^B NMR (2.96 ppm, ω_1/2_ = 49 Hz), evidencing the presence of a four-coordinate boron center. This was further validated by ^1^H NMR, as inequivalent methyl groups of the pinacolate were observed due to hindered rotation of the adduct. Single crystals suitable for X-ray crystallography were obtained by crystallization in CH_3_CN at −34 °C, unequivocally confirming the structure. It is noteworthy that with the precatalyst, the *N-*borylation occurs on the order of seconds rather than hours.

**Scheme 3:**
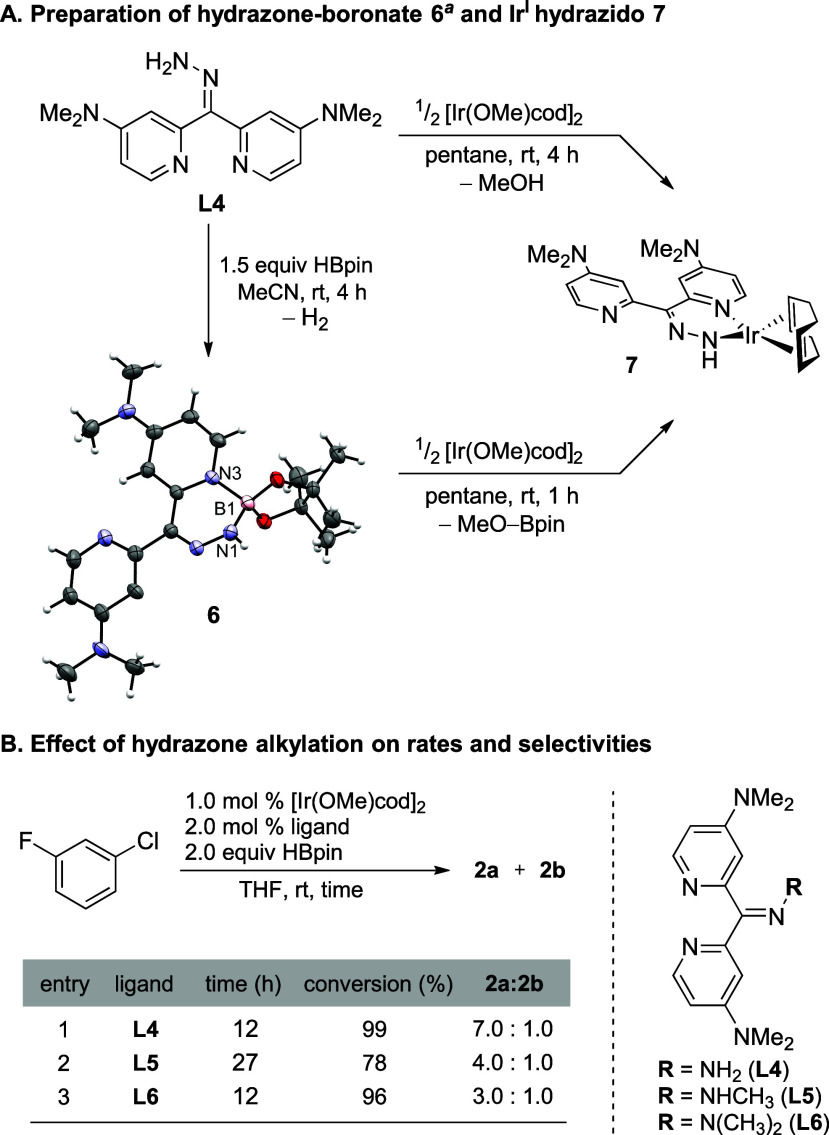
Investigation of the Hydrazone Ligand Framework Molecular structure displayed with 50% probability ellipsoids and a partial labeling scheme (cocrystallized CH_3_CN and H_2_O omitted for clarity). N1–B1 = 1.520 Å, N3–B1 = 1.587 Å.

We originally hypothesized that the hydrazone–boronate adduct **6** formed *in situ* during borylation and introduced an increased steric demand to the metal, improving selectivities. In practice, the stoichiometric reactions of both **L4** and **6** with [Ir(OMe)cod]_2_ in pentane lead to exclusive formation of the Ir^I^ hydrazido **7** (Scheme 3a) and methanol or MeO-Bpin, respectively. Further reaction of **7** with an additional equivalent of pinacolborane led to intractable mixtures of products. However, these results indicate that **L4** binds uniquely to iridium, unlike our previous work with **L2** or bipyridines. While catalytic amounts of material are difficult to characterize, the judicious choice of substrate can allow some analysis of the species generated during the reaction. Thus, the CHB of pentafluorobenzene was monitored via a NMR tube reaction (see the Supporting Information for details), and ^11^B NMR evidenced the formation of a new N–B bond during the reaction. Based on this evidence, both hydrogens in the amino hydrazone **L4** may be important for the reactivity and selectivity observed. To explore this, we synthesized substituted analogues of **L4** (Scheme 3b). Alkylation of the free amine of the hydrazone in **L5** and **L6** proved to be deleterious to both regioselectivity and activity. These results implicate the importance of amine in hydrazone **L4**. We hypothesize that the hydrazone amine forms both the Ir–hydrazido and the N–Bpin in the active catalyst.

Furthermore, we wanted to determine if the isolated hydrazido **7** and boronate adduct **6** lead to active catalyst formation by comparison with *in situ* generation in Table 1. When both were used for a borylation of fluorochlorobenzene (Scheme 4), selectivities nearly identical to those found when generating the catalyst *in situ* were observed. With these results in mind, a bis(boryl)Ir^III^ is likely operating in a canonical Ir^III^/Ir^V^ catalytic cycle.

**Scheme 4:**
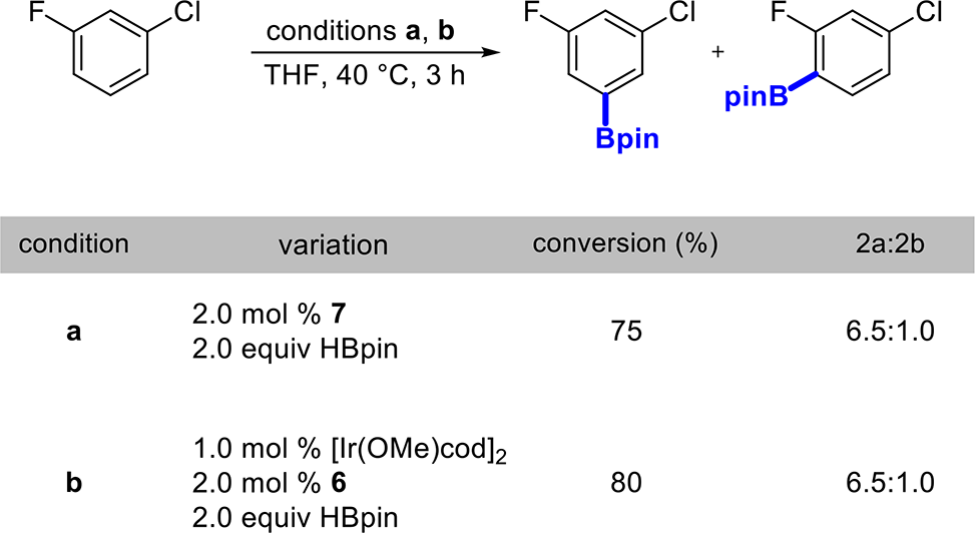
Borylation of Fluorochlorobenzene with Isolated Adduct and Ir^I^ Hydrazido See the Supporting Information for full details on the experimental procedures.

In summary, a new dipyridyl hydrazone ligand, dmadph, has been used in Ir-catalyzed C–H borylations of fluorinated arenes to afford significantly greater kinetic products than with dtbpy. We have shown that dmadph generates catalysts that are *both* more active and selective than those generated from dtbpy. Additionally, HBpin is utilized to increase m*e*ta selectivity, an effect that we previously observed with the dipyridylmethane type ligands.^32^ The origin of this unusual increase in regioselectivity using HBpin with both **L2***and***L4** is unclear at this time and warrants further investigations, which are ongoing.

## Supporting Information Available

Experimental procedures, including preparation of starting materials, compound characterization data, and product spectra (PDF)
