# Supplementary material for: A Hydrazone Ligand for Iridium-Catalyzed C–H Borylation: Enhanced Reactivity and Selectivity for Fluorinated Arenes
Source: Organometallics. 2024 May 20;43(11):1208–12. doi: 10.1021/acs.organomet.4c00174 (PMC11167643; doi:10.1021/acs.organomet.4c00174)
Supplement: Supplementary file 1 — om4c00174_si_001.pdf [file om4c00174_si_001.pdf]

A Hydrazone Ligand for Iridium-Catalyzed C–H Borylation: Enhanced Reactivity and Selectivity for Fluorinated Arenes

Christopher D. Peruzzi,[†] Susanne L. Miller,[†] Jonathan E. Dannatt,[†,‡] Behnaz Ghaffari,[†,§] Robert E. Maleczka, Jr,[†,*] and Milton R. Smith, III[†,*]

[†]Department of Chemistry, Michigan State University, 578 South Shaw Lane, East Lansing, Michigan, 48824, United States.
[‡]Department of Chemistry, University of Dallas, 1845 East Northgate Drive, Irving, Texas, 75062, United States.

Emails: maleczka@chemistry.msu.edu; smithmil@msu.edu

Supporting Information

I. General Information 2
II. Preparation of dmadph Ligand 4
III. Optimization of Reaction Conditions 7
IV. General Procedure for Borylation of Arenes and Hetero(arenes) 7
V. Characterization of Borylated Products 8
VI. Preparation of Ir^{I} hydrazido from dmadph and 6 18
VII. Borylation of fluorochlorobenzene with 6 and 7 19
VIII. Preparation of dmadph-borane adduct for X-ray crystallography 20
IX. NMR Tube Borylation of Pentafluorobenzene 21
X. Crystallographic Data for 6 21
XI. References 24
XII. NMR Spectra 25

I. General Information

Pinacolborane (HBpin) (97% stabilized with 1% triethylamine) was purchased commercially and used as received without further purification. The iridium catalyst, bis(η^4-1,5-cyclooctadiene)-di-μ-methoxy-diiridium (I), $[Ir(OMe)(cod)]_2$, was prepared by a literature procedure.[1]

All substrates were obtained commercially. Liquid substrates were purified by distillation and solid substrates were purified by sublimation or recrystallization.

All reactions were prepared in 3.0 mL Wheaton microreactor vials equipped with stir bars and pressure caps in a glovebox under a nitrogen atmosphere and then transferred to a preheated aluminum block outside of the glovebox. THF and *n*-hexane were obtained from wet stills refluxing over sodium and benzophenone. Methylene chloride and acetonitrile were obtained from dry stills according to the literature procedure.[2]

Reactions were monitored by ^{19}F NMR, and crude reaction ratios were verified by ^{1}H NMR or ^{19}F NMR for fluorine containing substrates. NMR spectra were recorded on a Varian 500 MHz DD2 Spectrometer equipped with a 1H-19F/15N-31P 5mm Pulsed Field Gradient (PFG) Probe. Spectra were taken in deuterated solvents referenced to residual solvent signals in ^{1}H NMR and ^{13}C{^{1}H} NMR. ^{13}C{^{1}H} NMR resonances for the boron-bearing carbon atom were not observed due to quadrupolar relaxation. NMR spectra were processed for display using the MNova software with only phasing and baseline corrections applied. For all NMR spectra, no peaks were manually corrected, suppressed or altered in any form, and unprocessed fids are available upon request.

Single crystal analyses were performed by Michigan State University Center for Crystallographic Research on a Charge Coupled Device (CCD diffractometer).

High-resolution mass spectra were obtained at the Michigan State University Mass Spectrometry Core using electron spray ionization (ESI+). Low resolution GC-MS was obtained on a Shimadzu GCMS-QP2010SE.

Silica used for purification of crude material was standard laboratory grade 230 - 400 mesh designed for flash chromatography applications. Purification of crude materials on a 1 mmol scale was achieved by standard flash chromatography methods employing 2-3 g silica gel plugs in small chromatography columns of dimension approximately 2 x 30 cm. The concentrated crude materials were dissolved in a minimum amount of solvent, applied to the silica gel with a Pasteur pipette

and eluted into test tubes. Compounds that eluted were visualized by spotting on TLC plates and irradiating with 254 nm UV light.

II. Preparation of dmadph Ligand

Synthesis of 4-(dimethylamino)picolinic acid hydrogen chloride[3]

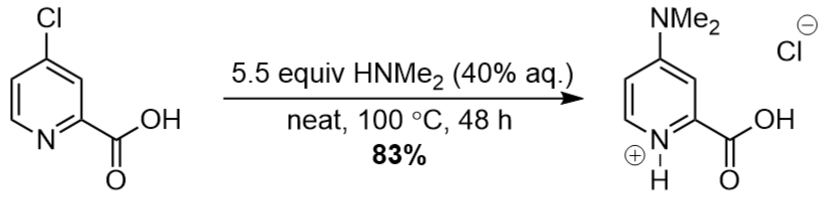

To a 350 mL pressure tube equipped with a stir bar, 4-chloropicolinic acid (18.0 g, 0.114 mol, 1 equiv) was added. Next, dimethylamine (40% in H_2O, 80.0 mL, 0.632 mol, 5.5 equiv) was added directly to the pressure tube. The pressure tube was then sealed and heated at 100 °C for 48 h. After cooling the pressure tube, the pH was adjusted to 11 using 10 M NaOH. The solvent was removed under vacuum, and the resulting solid was extracted with ethyl acetate (3 x 150mL). The solvent was removed on a rotary evaporator to yield the crude 4-(dimethylamino)picolinate. The solid was then recrystallized using a minimal amount of hot HCl (pH = 3) and allowing it to cool at -10 °C overnight. The resulting pale-yellow needles were filtered and washed with a 50/50 mixture of EtOH/Et_2O until the washings were colorless. The solid was then further dried to yield 4-(dimethylamino)picolinic acid hydrogen chloride (**15.6 g, 83%**) as white crystals (mp 236.0-239.8 °C dec).

^{1}H NMR (500 MHz, D_2O) δ 7.78 (d, *J* = 7.2 Hz, 1H), 7.09 (d, *J* = 2.9 Hz, 1H), 6.68 (dd, *J* = 7.3, 3.0 Hz, 1H), 3.00 (s, 6H).

^{13}C{^{1}H} NMR (126 MHz, D_2O) δ 162.9, 157.9, 139.4, 138.4, 108.0, 107.6, 39.7, 39.5.

Synthesis of ethyl-4-(dimethylamino)picolinate

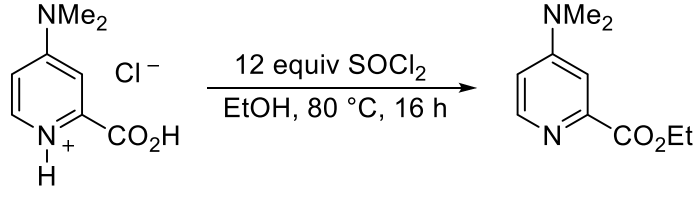

The title compound was synthesized according to a known literature procedure[3] for the analogous methyl ester, with a slight modification to the work up procedure as follows.

A 250 mL 3-neck round bottom flask was equipped with a Dimroth condenser, pressure equalized addition funnel, and a stir bar. 4-(Dimethylamino)picolinic acid hydrogen chloride (5.127 g, 25.3 mmol, 1 equiv) was weighed into the flask followed by 65 mL of ethanol. Thionyl chloride (22.0

mL, 303.3 mmol, 12 equiv) was added to the addition funnel and the flask was then cooled to 0 °C. Thionyl chloride was added dropwise over a period of 25 minutes while keeping the solution close to 0 °C. When the addition was complete, the solution was brought to reflux for 16 hours. Upon cooling, the solution was quenched using sat. aq. $NaHCO_3$ until pH = 7. The pH was balanced to approximately pH = 10 using 10 M NaOH and the resulting solution was extracted with CH_2Cl_2 (3 x 60 mL). The combined CH_2Cl_2 layers were passed through a plug of basic alumina and dried over $MgSO_4$. The drying agent was then gravity filtered and the solvent removed under vacuum to yield ethyl-4-(dimethylamino)picolinate (**5.134 g, 88%**) as an orange oil.

^{1}H NMR (500 MHz, $CDCl_3$) δ 8.33 (d, *J* = 5.9 Hz, 1H), 7.39 (d, *J* = 2.7 Hz, 1H), 6.59 (dd, *J* = 5.9, 2.8 Hz, 1H), 4.45 (q, *J* = 7.1 Hz, 2H), 3.05 (s, 6H), 1.43 (t, *J* = 7.1 Hz, 3H).

^{13}C{^{1}H} NMR (126 MHz, $CDCl_3$) δ 166.4, 154.8, 149.8, 148.4, 108.7, 108.1, 61.7, 39.2, 14.4.

Synthesis of bis(4-(dimethylamino)pyridin-2-yl)methanone

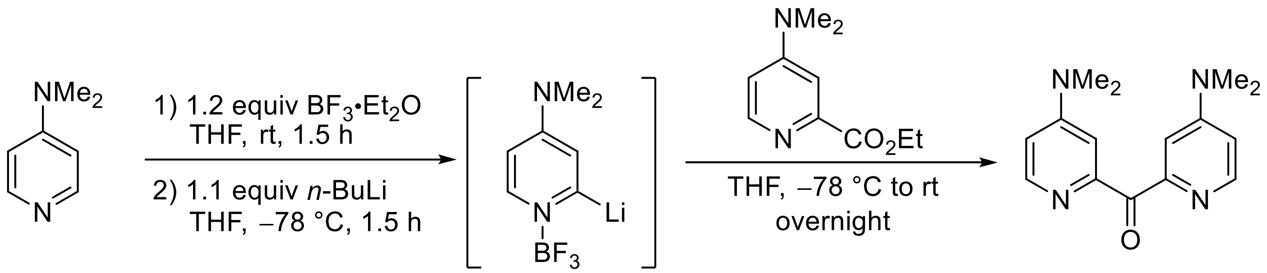

To an oven-dried, 250 mL round bottom flask equipped with a stir bar, 4-(dimethylamino)pyridine (0.7436 g, 6.0 mmol, 1.0 equiv) was added along with 90 mL of dry THF. The clear solution was sparged with N_2 for 30 minutes. After this, $BF_3{\cdot}OEt_2$ (0.890 mL, 7.2 mmol, 1.2 equiv) was added dropwise to the DMAP solution causing it to briefly warm up and turn the clear, colorless solution to a slightly cloudy and yellow solution and stirred for 1 h at room temperature. Afterwards, the mixture was cooled to –78 °C and the temperature was carefully monitored with a thermocouple probe inserted through a septum into the mixture. Next, *n*-BuLi (2.5 M in hexanes, 2.60 mL, 1.1 equiv) was added dropwise via syringe over 20 minutes, ensuring that the temperature did not rise above –70 °C. The solution was stirred cold at –78 °C for 1.5 h. Separately, ethyl-4-(dimethylamino)picolinate was added to a 300 mL, 3-neck round bottom flask equipped with a stir bar. Under nitrogen, 50 mL of THF was added and the solution was cooled to to –78 °C. After 1.5 h of stirring, the lithiated 4-(dimethylamino)pyridine was cannula transferred dropwise to the cooled solution of ethyl-4-(dimethylamino)picolinate, causing the light-yellow solution to become

a deep, golden yellow-orange. The mixture was stirred cold for 1.5 h and then quenched with 1.0 mL of anhydrous EtOH and allowed to come to rt overnight. The solvent was removed on a rotary evaporator and the solid dissolved in CH_2Cl_2 (80 mL) and washed with a 1M KOH/1% ethylene glycol solution (3x30mL). After drying on Na_2SO_4, the solvent was again removed on a rotary evaporator to yield a yellow-white solid which was dissolved in CH_2Cl_2 and washed with EtOAc (3x20 mL). The CH_2Cl_2 was dried again and evaporated to yield a white powder of bis(4-(dimethylamino)pyridin-2-yl)methanone (0.5232 g, 32% yield). The spectral data matched with previously reported literature values.[4]

Synthesis of 2,2'-(hydrazineylidenemethylene)bis(N,N-dimethylpyridin-4-amine) (L4)

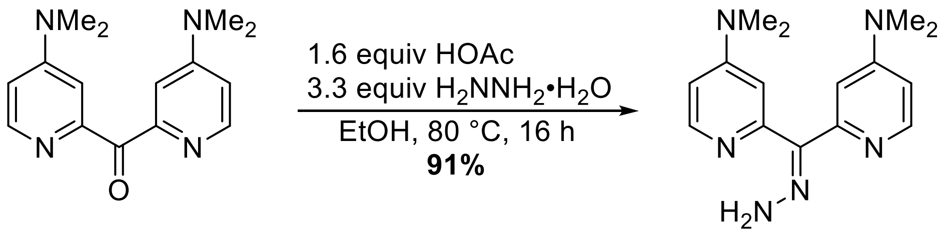

To a 25 mL, heavy walled Schlenk flask equipped with a stir bar, bis(DMAP)methanone (0.523 g, 1.9 mmol, 1 equiv) was added and dissolved in 15 mL of anhydrous ethanol with stirring. At room temperature, acetic acid (0.177 mL, 3.1 mmol, 1.6 equiv) was added via syringe with vigorous stirring, and the color of the solution turned to a pale yellow. Then, hydrazine hydrate (65%, 0.462 mL, 6.2 mmol, 3.3 equiv) was added via a syringe to the solution, which caused it to become an orange color. The reaction was stirred at 70 °C for 4 h and allowed to cool back down to rt. The solvent was then carefully removed under vacuum until a white precipitate formed. The resulting white solid was collected by filtration and washed with several portions of 2-3 mLs of ice-cold isopropanol. The mother liquor was subsequently concentrated to yield a second crop of the white solid, which was also washed with isopropanol. The combined white solids were then recrystallized in a minimal amount of ^{i}PrOH to yield dmadph (**0.511 g, 91%**) as white needles (mp 149.0-152.0 °C dec.)

^{1}H NMR (500 MHz, $CDCl_3$) δ 8.30 (d, *J* = 6.0 Hz, 1H), 8.20 (d, *J* = 5.9 Hz, 1H), 7.46 (s, 2H), 7.04 (d, *J* = 2.7 Hz, 1H), 6.54 (d, *J* = 2.7 Hz, 1H), 6.47 (dd, *J* = 6.0, 2.7 Hz, 1H), 6.44 (dd, *J* = 5.9, 2.7 Hz, 1H), 3.03 (s, 6H), 2.94 (s, 6H).

^{13}C{^{1}H} NMR (126 MHz, $CDCl_3$) δ 157.3, 154.8, 153.0, 148.7, 148.5, 145.2, 108.1, 106.1, 105.7, 105.0, 39.2, 39.1.

III. Optimization of Reaction Conditions

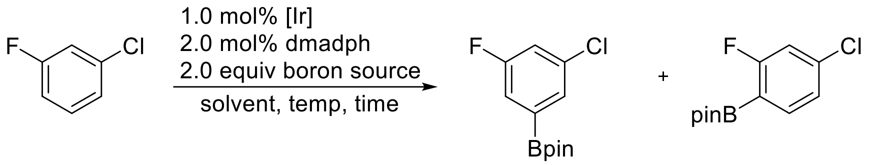

entry	[Ir]	boron source	solvent	temp (°C)	time (h)	%conversion	ratio m : o
1	$[Ir(OMe)cod]_2$	B_2pin_2	THF	25	24	>99	2.9 : 1.0
2	$[Ir(OMe)cod]_2$	HBpin	THF	25	24	>99	7.8 : 1.0
3	$[Ir(OMe)cod]_2$	HBpin	DCM	25	24	>99	4.3 : 1.0
4	$[Ir(Cl)cod]_2$	HBpin	DCM	25	24	71	4.1 : 1.0
5	$[Ir(OMe)cod]_2$	HBpin	THF	40	1	98	6.3 : 1.0
6	$[Ir(Cl)cod]_2$	HBpin	THF	40	1	24	5.9 : 1.0
7	$[Ir(OMe)cod]_2$	HBpin	THF	60	1	>99	4.4 : 1.0
8	$[Ir(OMe)cod]_2$	HBpin	THF	50	2	>99	5.7 : 1.0

IV. General Procedure for Borylation of Arenes and Hetero(arenes)

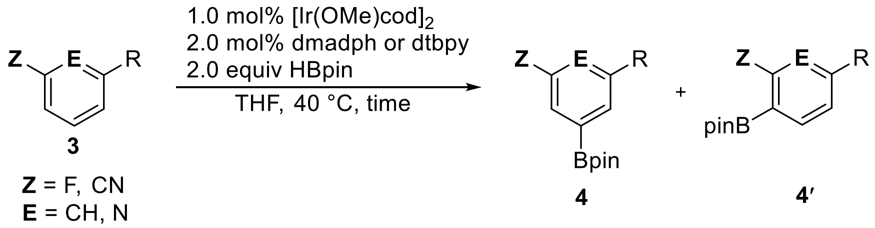

In a nitrogen-filled glovebox, a 3.0 mL Wheaton pressure vial equipped with a stir bar was charged with a 1.0 mL THF solution of $[Ir(OMe)cod]_2$ (6.6 mg, 0.01 equiv). To this solution, pinacolborane (0.290 mL, 2 mmol, 2.0 equiv) was added with a syringe while stirring, turning the light-yellow solution into a golden orange. Then, in a small test tube, a 1.0 mL THF solution of dmadph (5.6 mg, 0.02 equiv) or dtbpy (5.4 mg, 0.02 equiv) was made and added to the iridium solution with a syringe and the solution immediately turned dark red in color. Last, the substrate was added to this solution and capped. The reaction was heated at 40 °C and stirred in an aluminum heating block on top of a stir plate. Reactions were monitored by ^{19}F and ^{1}H NMR spectroscopies. When reactions were completed, the volatiles were evaporated, and the crude reaction mixtures purified by silica gel chromatography in 100% CH_2Cl_2. The borylated products were isolated as the regioisomeric mixtures.

V. Characterization of Borylated Products

Borylation of 2-chloro-6-fluoropyridine (4a)

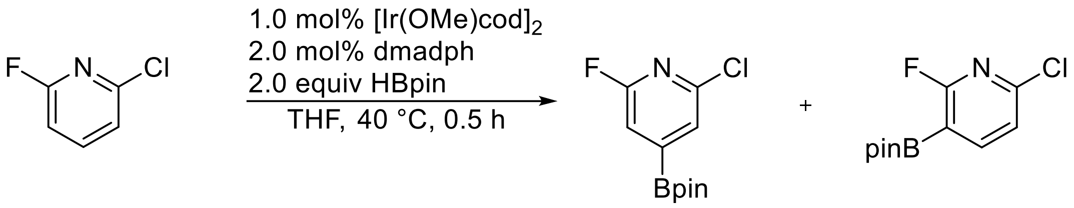

4a **4a′**

Isolated as a white, crystalline solid (**88%, >20:1.0 m:o selectivity**) after column chromatography in 100% CH_2Cl_2. Conversion was determined from ^{19}F NMR to be >99% at 30 min. The spectral data agree with previously reported literature values.[4] (mp 77.8-80.1 °C)

Crude selectivity via ^{19}F NMR was found to be >20:1.0 4a:4a′.

The selectivity found via ^{19}F NMR for dtbpy was 5.8:1.0 m:o to F.

^{1}H NMR of 4a (500 MHz, $CDCl_3$) δ 7.56 (d, *J* = 1.7 Hz, 1H), 7.20 (d, *J* = 2.6 Hz, 1H), 1.35 (s, 12H)
^{1}H NMR of 4a′ (500 MHz, $CDCl_3$) δ 8.11 (t, *J* = 8.1 Hz, 1H), 7.22 (dd, *J* = 7.5 Hz, 1.6 Hz, 1H), 1.35 (s, 12H)

^{13}C{^{1}H} NMR of 4a (126 MHz, $CDCl_3$) δ 162.3 (d, *J* = 247.6 Hz), 148.7 (d, *J* = 12.8 Hz), 126.6 (d, *J* = 4.8 Hz), 112.9 (d, *J* = 33.0 Hz), 85.3, 25.0.

^{19}F NMR of 4a (470 MHz, $CDCl_3$) δ -67.38

^{19}F NMR of 4a′ (470 MHz, $CDCl_3$) δ -55.95

^{11}B NMR (160 MHz, $CDCl_3$) δ 29.69 (br s)

Borylation of isophthalonitrile (4b)

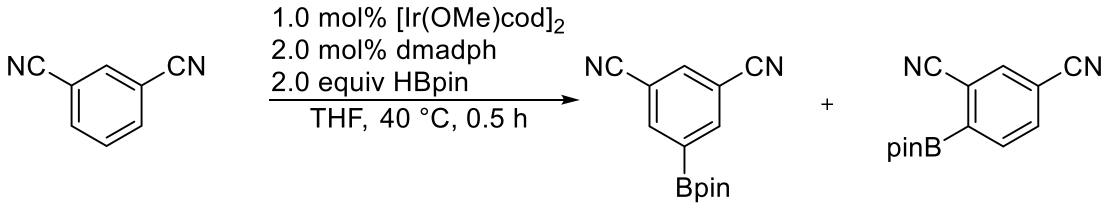

4b **4b′**

Isolated as a white, crystalline solid (**85%, 14.3:1.0 m:o selectivity**) after column chromatography in 100% CH_2Cl_2. Conversion was determined from ^{19}F NMR to be >99% at 30 min. The spectral data agree with previously reported literature values.[4]

Crude selectivity via ^{19}F NMR was found to be 12.5:1.0 4b:4b′.

The selectivity found via ^{1}H NMR analysis for dtbpy was 4.4:1.0 m:o to CN.

^{1}H NMR of 4b (500 MHz, $CDCl_3$) δ 8.26 (d, *J* = 1.6 Hz, 2H), 7.98 (t, *J* = 1.6 Hz, 1H), 1.35 (s, 12H).

^{1}H NMR of 4b′ (500 MHz, $CDCl_3$) δ 8.01 (d, *J* = 7.9 Hz, 1H), 7.96-7.95 (m, 1H), 7.83 (dd, *J* = 7.8, 1.5 Hz, 1H) 1.38 (s, 12H).

$^{13}C\{^{1}H\}$ NMR of 4b (126 MHz, $CDCl_3$) δ 141.8, 137.1, 116.7, 113.6, 85.3, 24.8.

$^{13}C\{^{1}H\}$ NMR of 4b′ (126 MHz, $CDCl_3$) δ 136.7, 136.1, 136. 0, 134.4, 118.7, 116.9, 116.7, 115.4, 85.6, 24.8.

^{11}B NMR (160 MHz, $CDCl_3$) δ 29.73 (br s)

Borylation of 3-(trifluoromethoxy)fluorobenzene (4c)

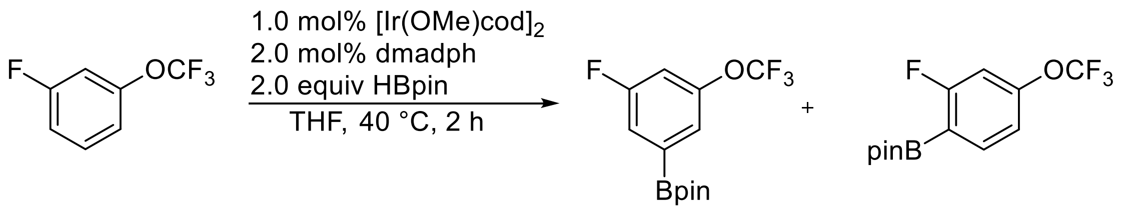

4c **4c′**

Isolated as a colorless, clear liquid (**96%, 8.0:1.0 m:o selectivity**) after column chromatography in 100% CH_2Cl_2. Conversion was determined from ^{19}F NMR to be >99% at 2 h.

Crude selectivity via ^{19}F NMR was found to be 8.0:1.0 4c:4c′.

The selectivity found via ^{19}F NMR for dtbpy was 4.1:1.0 m:o to F.

^{1}H NMR of 4c (500 MHz, $CDCl_3$) δ 7.44-7.42 (m, 2H), 7.03 (ddd, *J* = 9, 2.5, 1.5 Hz, 1H), 1.35 (s, 12H).

^{1}H NMR of 4c′ (500 MHz, CDCl$_3$) δ 7.78 (dd, *J* = 8.5, 1.5 Hz, 1H), 6.99 – 7.02* (m, 1H), 6.92 (dq, *J* = 9.5, 0.9 Hz, 1H), 1.36 (s, 12H).

^{13}C{^{1}H} NMR of 4c (126 MHz, CDCl$_3$) δ 162.4 (d, *J* = 250.1 Hz), 138.0 (d, *J* = 9.7 Hz), 122.3 (d, *J* = 3.1 Hz), 119.5 (d, *J* = 19.3 Hz), 111.6 (d, *J* = 25.0 Hz), 84.5, 24.7.

^{13}C{^{1}H} NMR of 4c′ (126 MHz, CDCl$_3$) δ 167.4 (d, *J* = 254.0 Hz), 149.4 (d, *J* = 9.9 Hz), 121.4, 115.1 (d, *J* = 3.8 Hz), 108.2 (d, *J* = 28.3 Hz), 84.1, 24.7.

^{19}F NMR of 4c (470 MHz, CDCl$_3$) δ -57.92 (3F), -110.87 (1F)

^{19}F NMR of 4c′ (470 MHz, CDCl$_3$) δ -57.81 (3F), -98.93 (1F)

^{11}B NMR (160 MHz, CDCl$_3$) δ 30.06 (br s)

*apparent doublet of quartets with matching coupling constants to the resonance at 6.92 ppm, one of the quartets is buried under the major isomer, however.

Borylation of 3-(trifluoromethyl)fluorobenzene (4d)

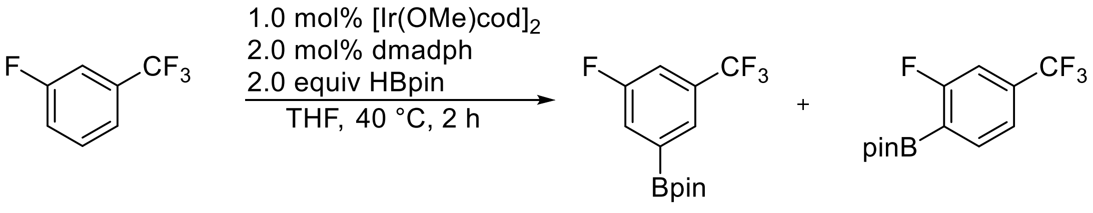

Isolated as a colorless, clear liquid (**87%, 4.4:1.0 m:o selectivity**) after column chromatography in 100% CH_2Cl_2. Conversion was determined from ^{19}F NMR to be >99% at 2 h. The spectral data agree with previously reported literature values.[5,6]

Crude selectivity via ^{19}F NMR was found to be 4.4:1.0 4d:4d′.

The selectivity found via ^{19}F NMR for dtbpy was 1.8:1.0 m:o to F.

^{1}H NMR of 4d (500 MHz, CDCl$_3$) δ 7.84 (s, 1H), 7.65 (dd, *J* = 8.5, 2.5 Hz, 1H), 7.40-7.37 (m, 1H), 1.36 (s, 12H).

^{1}H NMR of 4d′* (500 MHz, CDCl$_3$) δ 7.86 (t, *J* = 7.0 Hz, 1H), 7.40 (m, 1H), 7.29 (d, *J* = 8.9 Hz, 1H), 1.37 (s, 12H).

^{13}C{^{1}H} NMR of 4d (126 MHz, CDCl$_3$) δ 162.1 (d, *J* = 249.5 Hz), 132.0 (dd, *J* = 33.2, 7.0 Hz), 127.0 (p, *J* = 3.8 Hz), 124.6 (d, *J* = 19.5 Hz), 115.3 (dq, *J* = 24.5, 3.7 Hz), 84.6, 24.8.

$^{13}C\{^1H\}$ NMR of 4d′ (126 MHz, $CDCl_3$) δ 166.7 (d, *J* = 253.2 Hz), 137.6 (d, *J* = 8.3 Hz), 120.4 – 120.1 (m), 112.8 – 112.2 (m), 84.4, 24.8.

^{19}F NMR of 4d (470 MHz, $CDCl_3$) δ -62.69 (3F), -111.94 (1F).

^{19}F NMR of 4d′ (470 MHz, $CDCl_3$) δ -100.52

^{11}B NMR (160 MHz, $CDCl_3$) δ 30.15 (br s)

*Resonance at 7.86 buried under major isomer, assumed triplet from previously reported literature values. Resonance at 7.40 completely buried under major isomer, but visible, matching the chemical shift from previously reported values.[5]

Borylation of 3-fluorochlorobenzene (4e)

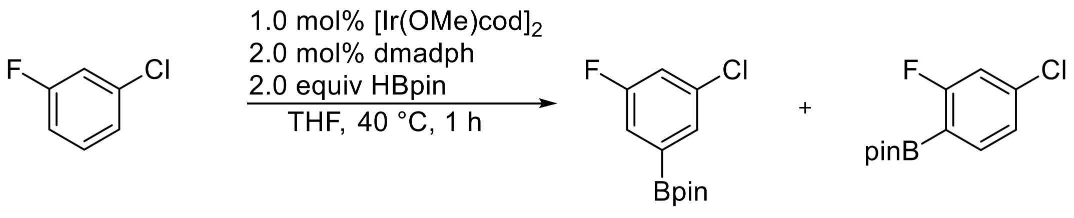

Isolated as a colorless, clear liquid (**82%, 6.1:1.0 m:o selectivity**) after column chromatography in 100% CH_2Cl_2. Conversion was determined from ^{19}F NMR to be 98% at 1 h. The spectral data agree with previously reported literature values.[4]

Crude selectivity via ^{19}F NMR was found to be 6.4:1.0 4e:4e′.

The selectivity found via ^{19}F NMR for dtbpy was 2.5:1.0 m:o to F.

^1H NMR of 4e (500 MHz, $CDCl_3$) δ 7.56 (d, *J* = 1.1 Hz, 1H), 7.37 (dd, *J* = 8.5, 2.0 Hz, 1H), 7.16 (dt, *J* = 8.6, 2.3 Hz, 1H), 1.34 (s, 12H).

^1H NMR of 4e′ (500 MHz, $CDCl_3$) δ 7.67 (dd, *J* = 7.8, 6.7 Hz, 1H), 7.14 (dd, *J* = 8.3, 2.0 Hz, 1H), 7.07 (dd, *J* = 9.0, 1.7 Hz, 1H), 1.34 (s, 12H).

$^{13}C\{^1H\}$ NMR of 4e (126 MHz, $CDCl_3$) δ 162.3 (d, *J* = 250.5 Hz), 134.7 (d, *J* = 8.82 Hz), 130.3 (d, *J* = 3.0 Hz), 119.3 (d, *J* = 19.3 Hz), 118.7 (d, *J* = 24.6 Hz), 84.4, 24.8.

$^{13}C\{^1H\}$ NMR of 4e′ (126 MHz, $CDCl_3$) δ 167.1 (d, *J* = 254.7 Hz), 138.4 (d, *J* = 10.6 Hz), 137.6 (d, *J* = 9.2 Hz), 124.1 (d, *J* = 3.4 Hz), 116.1 (d, *J* = 27.6 Hz), 84.0, 24.8.

^{19}F NMR of 4e (470 MHz, $CDCl_3$) δ -111.94

^{19}F NMR of 4e′ (470 MHz, $CDCl_3$) δ -100.52

^{11}B NMR (160 MHz, $CDCl_3$) δ 30.15 (br s)

Borylation of 3-fluorobromobenzene (4f)

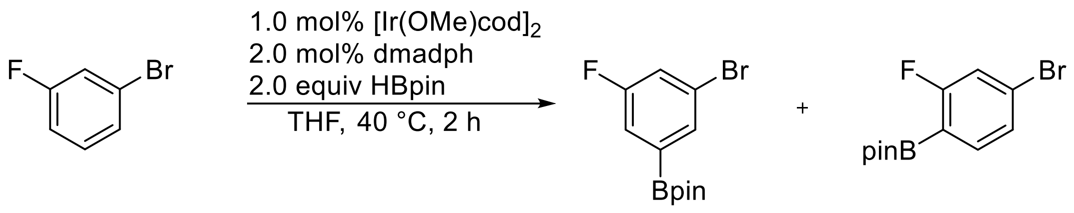

4f **4f′**

Isolated as a colorless, clear liquid (**93%, 7.2:1.0 m:o selectivity**) after column chromatography in 100% CH_2Cl_2. Conversion was determined from ^{19}F NMR to be 97% at 2 h. The spectral data agree with previously reported literature values.[7]

Crude selectivity via ^{19}F NMR was found to be 7.1:1.0 4f:4f′.

The selectivity found via ^{19}F NMR for dtbpy was 2.9:1.0 m:o to F.

^{1}H NMR of 4f (500 MHz, $CDCl_3$) δ 7.71 (dd, *J* = 1.75, 0.70 Hz, 1H), 7.41 (ddd, *J* = 8.5, 2.5, 0.6 Hz, 1H), 7.32 (ddd, *J* = 8.3, 2.5, 0.60 Hz, 1H), 1.34 (s, 12H).

^{1}H NMR of 4f′ (500 MHz, $CDCl_3$) δ 7.60 (dd, *J* = 8.0, 6.5 Hz, 1H), 7.29 (dd, *J* = 8.0, 1.7 Hz, 1H), 7.23 (dd, *J* = 8.6, 1.7 Hz, 1H), 1.35 (s, 12H).

^{13}C{^{1}H} NMR of 4f (126 MHz, $CDCl_3$) δ 162.3 (d, *J* = 251.6 Hz), 133.3 (d, *J* = 3.1 Hz), 121.6 (d, *J* = 24.4 Hz), 119.7 (d, *J* = 19.3 Hz), 84.4, 24.8.

^{13}C{^{1}H} NMR of 4f′ (126 MHz, $CDCl_3$) δ 166.9 (d, *J* = 255.7 Hz), 137.8 (d, *J* = 8.8 Hz), 127.1 (d, *J* = 3.4 Hz), 126.4 (d, *J* = 10.0 Hz), 119.0 (d, *J* = 27.4 Hz), 84.1, 24.8.

^{19}F NMR of 4f (470 MHz, $CDCl_3$) δ -111.56

^{19}F NMR of 4f′ (470 MHz, $CDCl_3$) δ -100.30

^{11}B NMR (160 MHz, $CDCl_3$) δ 30.11 (br s)

Borylation of 1,3-difluorobenzene (4g)

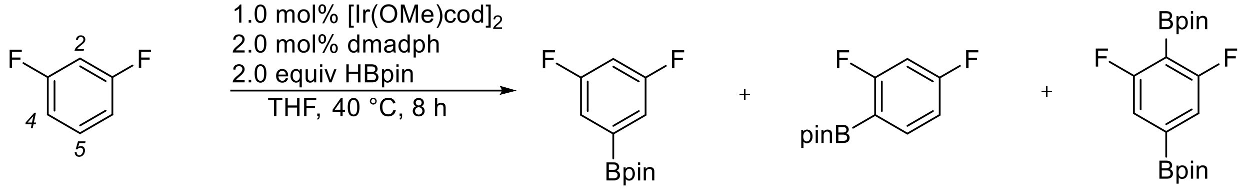

4g **4g′** **4g′′**

Isolated as a white solid (**88%, 4.3:1.0:0.9 5:4:2,5-diborylated selectivity**) after column chromatography in 100% CH_2Cl_2. Conversion was determined from ^{19}F NMR to be >99% at 8 h. The spectral data agree with previously reported literature values for the 4- and 5-borylated isomers.[8] The resonances at δ -94.1 (corresponding to the 4,6-diborylated isomer) and -100.6 (corresponding to the 2-borylated isomer) represent 2% of the regioisomeric mixture.

Crude selectivity via ^{19}F NMR was found to be 4.6:1.0:0.9 4g:4g′:4g′′.

The selectivity found via ^{19}F NMR for dtbpy was 3.3:2.7:1.0 of the 5:4:2,5-diborylated isomers.

^{1}H NMR of 4g (500 MHz, $CDCl_3$) δ 7.27 (dt, *J* = 6.1, 2.2 Hz, 2H), 6.86 (tt, *J* = 8.9, 2.4 Hz, 1H), 1.33 (s, 12H).

^{1}H NMR of 4g′ (500 MHz, $CDCl_3$) δ 7.74 – 7.70 (m, 1H), 6.88 – 6.83* (m, 1H), 6.75 (td, *J* = 9.5, 2.3 Hz, 1H), 1.37 (s, 12H).

^{1}H NMR of 4g′′ (500 MHz, $CDCl_3$) δ 7.23 – 7.25 (m, 2H), 1.34 (s, 12H).

^{13}C{^{1}H} NMR of 4g (126 MHz, $CDCl_3$) δ 162.7 (dd, *J* = 249.7, 11.0 Hz), 116.8 (dd, *J* = 17.7, 5.0 Hz), 106.4 (t, *J* = 25.1 Hz), 84.4, 24.8.

^{13}C{^{1}H} NMR of 4g′ (126 MHz, $CDCl_3$) δ 165.7 (dd, *J* = 289.7, 12.2 Hz), 116.7 – 115.9 (m), 111.1 (dd, *J* = 20.2, 3.6 Hz), 103.6 (dd, *J* = 27.9, 24.3 Hz), 83.9, 24.7.

^{19}F NMR of 4g (470 MHz, $CDCl_3$) δ -110.79

^{19}F NMR of 4g′ (470 MHz, $CDCl_3$) δ -98.63, -105.13

^{19}F NMR of 4g′′ (470 MHz, $CDCl_3$) δ -101.80

^{11}B NMR (160 MHz, $CDCl_3$) δ 30.02 (br s)

*Resonance is completely buried underneath the major isomer. An irregular peak shape for the major isomer provides evidence of the resonance and is in agreement with previous reported values.

Borylation of 3-fluorobenzonitrile (4h)

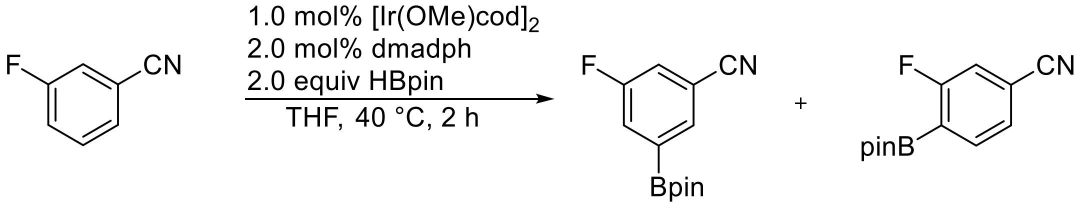

4h **4h′**

Isolated as a colorless, clear liquid (**93%, 3.5:1.0 m:o selectivity**) after column chromatography in 100% CH_2Cl_2. Conversion was determined from ^{19}F NMR to be >99% at 2 h.

Crude selectivity via ^{19}F NMR was found to be 3.4:1.0 4h:4h′.

The selectivity found via ^{19}F NMR for dtbpy was 1.1:1.0 m:o to F.

^{1}H NMR of 4h (500 MHz, $CDCl_3$) δ 7.88 (s, 1H), 7.70 (dd, *J* = 8.5, 0.6 Hz, 1H), 7.41 (ddd, *J* = 8.1, 2.6, 1.4 Hz, 1H), 1.35 (s, 12H).

^{1}H NMR of 4h′ (500 MHz, $CDCl_3$) δ 7.62 (t, *J* = 6.7 Hz, 1H), 6.95* (m, 1H), 6.86 (d, *J* = 10.4 Hz, 1H), 1.36 (s, 12H).

^{13}C{^{1}H} NMR of 4h (126 MHz, $CDCl_3$) δ 161.7 (d, *J* = 251.0 Hz), 137.8 (d, *J* = 8.7 Hz), 134.2 (d, *J* = 3.3 Hz), 125.8 (d, *J* = 19.4 Hz), 121.1 (d, *J* = 24.7 Hz), 84.8, 24.7.

^{13}C{^{1}H} NMR of 4h′ (126 MHz, $CDCl_3$) δ 166.2 (d, *J* = 254.7 Hz), 127.2 (d, *J* = 3.7 Hz), 118.7 (d, *J* = 27.6 Hz), 117.4 (d, *J* = 3.0 Hz), 113.5 (d, *J* = 8.5 Hz), 84.6, 24.7.

^{19}F NMR of 4h (470 MHz, $CDCl_3$) δ -111.07

^{19}F NMR of 4h′ (470 MHz, $CDCl_3$) δ -99.87

^{11}B NMR (160 MHz, $CDCl_3$) δ 29.96 (br s)

*Resonance at 6.95 is completely buried under major isomer.

Borylation of 3-fluorotoluene (4i)

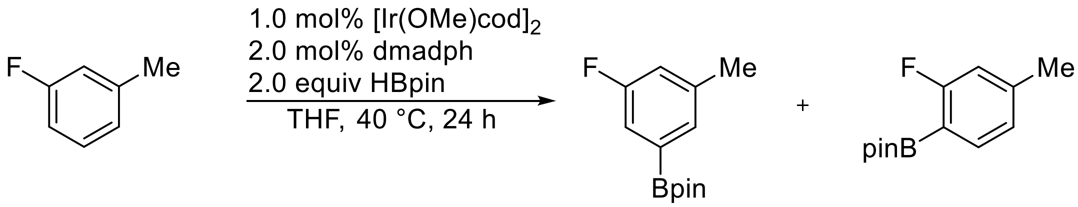

4i **4i′**

Isolated as a colorless, clear liquid (**72%, 4.2:1.0 m:o selectivity**) after column chromatography in 100% CH_2Cl_2. Conversion was determined from ^{19}F NMR to be >99% at 24 h. The spectral data agree with previously reported literature values.[5]

Crude selectivity via ^{19}F NMR was found to be 4.0:1.0 4i:4i′.

The selectivity found via ^{19}F NMR for dtbpy was 1.4:1.0 m:o to F.

^{1}H NMR of 4i (500 MHz, CDCl₃) δ 7.40 (s, 1H), 7.28 (dd, *J* = 8.9, 2.0 Hz, 1H), 6.98-6.95 (m, 1H), 2.36 (s, 3H), 1.35 (s, 12H).

^{1}H NMR of 4i′ (500 MHz, $CDCl_3$) δ 7.40 (s, 1H), 7.28 (dd, *J* = 8.9, 2.0 Hz, 1H), 6.98-6.95 (m, 1H), 2.36 (s, 3H), 1.35 (s, 12H).

^{13}C{^{1}H} NMR of 4i (126 MHz, $CDCl_3$) δ 162.5 (d, *J* = 246.4 Hz), 139.8 (d, *J* = 7.0 Hz), 136.6 (d, *J* = 8.7 Hz), 131.0 (d, *J* = 2.5 Hz), 118.8 (d, *J* = 20.9 Hz), 117.9 (d, *J* = 19.3 Hz), 84.0, 24.8, 21.1.

^{13}C{^{1}H} NMR of 4i′ (126 MHz, $CDCl_3$) δ 144.3 (d, *J* = 8.7 Hz), 136.6 (d, *J* = 8.7 Hz), 124.5 (d, *J* = 2.8 Hz), 115.8 (d, *J* = 23.9 Hz), 83.7, 24.8, 21.0.

^{19}F NMR of 4i (470 MHz, $CDCl_3$) δ -115.35

^{19}F NMR of 4i′ (470 MHz, $CDCl_3$) δ -103.84

^{11}B NMR (160 MHz, $CDCl_3$) δ 30.53 (br s)

Borylation of 3-fluoroanisole (4j)

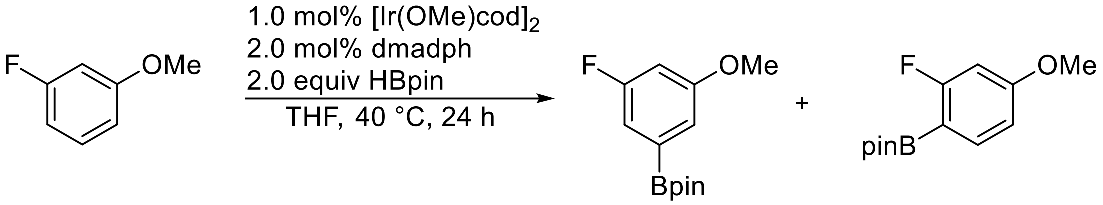

4j **4j′**

Isolated as a colorless, clear liquid (**80%, 5.8:1.0 m:o selectivity**) after column chromatography in 100% CH_2Cl_2. Conversion was determined from ^{19}F NMR to be 88% at 24 h. The spectral data agree with previously reported literature values.[5]

Crude selectivity via ^{19}F NMR was found to be 6.0:1.0 4j:4j′.

The selectivity found via ^{19}F NMR for dtbpy was 2.2:1.0 m:o to F.

^{1}H NMR 4j (500 MHz, CDCl$_3$) δ 7.10 (d, *J* = 5 Hz, 1H), 7.09 (dd, *J* = 8.5, 2.4 Hz, 1H), 6.70 (dt, *J* = 10, 5 Hz, 1H), 3.82 (s, 3H), 1.34 (s, 12H).

^{1}H NMR 4j′ (500 MHz, CDCl$_3$) δ 7.65 (dd, *J* = 8.3, 7.2 Hz, 1H), 6.68* (dd, *J* = 2.5 Hz, 1H), 6.57 (dd, *J* = 11.3, 2.3 Hz, 1H), 3.81 (s, 3H), 1.35 (s, 12H).

^{13}C{^{1}H} NMR of 4j (126 MHz, CDCl$_3$) δ 163.2 (d, *J* = 246.3 Hz), 160.5 (d, *J* = 10.3 Hz), 114.7 (d, *J* = 2.5 Hz), 113.3 (d, *J* = 19.7 Hz), 105.1 (d, *J* = 24.7 Hz), 84.1, 55.6, 24.8.

^{13}C{^{1}H} NMR of 4j′ (126 MHz, CDCl$_3$) δ 168.5 (d, *J* = 250.9 Hz), 137.7 (d, *J* = 10.5 Hz), 109.9 (d, *J* = 2.7 Hz), 101.1 (d, *J* = 28.0 Hz), 83.6, 55.5, 24.8.

^{19}F NMR of 4j (470 MHz, CDCl$_3$) δ -57.92 (3F), -110.87 (1F)

^{19}F NMR of 4j′ (470 MHz, CDCl$_3$) δ -57.81 (3F), -98.93 (1F)

^{11}B NMR (160 MHz, CDCl$_3$) δ 30.45 (br s)

*Agrees with previous reported literature values[5], the only coupling constant visible is given, and the other half of the resonance is buried under the major isomer.

Borylation of 3-fluoro-*N,N*-dimethylaniline (4k)

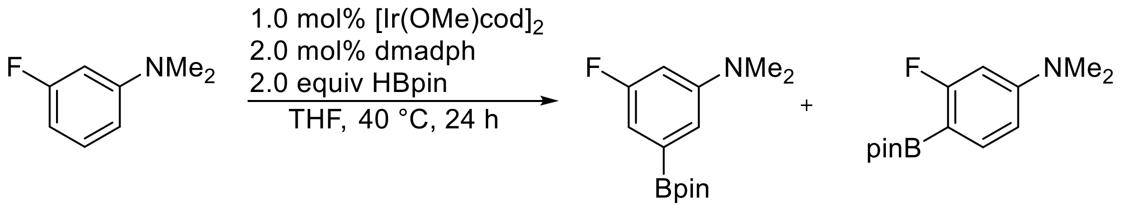

4k **4k′**

Isolated as a white solid (**93%, 3.1:1.0 m:o selectivity**) after column chromatography in 100% CH_2Cl_2. Conversion was determined from ^{19}F NMR to be 71% at 24 h. The spectral data agree with previously reported literature values.[5]

Crude selectivity via ^{19}F NMR was found to be 3.3:1.0 4k:4k′.

The selectivity found via ^{19}F NMR for dtbpy was 1.9:1.0 m:o to F.

^{1}H NMR 4k (500 MHz, CDCl$_3$) δ 6.94 (d, *J* = 2.3 Hz, 1H), 6.84 (dd, *J* = 8.5, 2.3 Hz, 1H), 6.49 (dt, *J* = 12.6, 2.3 Hz, 1H), 2.97 (s, 6H), 1.35 (s, 12H).

^{1}H NMR 4k′ (500 MHz, CDCl$_3$) δ 7.59 (t, *J* = 7.9 Hz, 1H), 6.44 (dd, *J* = 8.5, 2.2 Hz, 1H), 6.31 (dd, *J* = 13.4, 2.2 Hz, 1H), 2.98 (s, 6H), 1.35 (s, 12H).

^{19}F NMR of 4k (470 MHz, CDCl$_3$) δ -113.79.

^{19}F NMR of 4k′ (470 MHz, CDCl$_3$) δ -101.65.

$^{13}C\{^1H\}$ NMR of 4k (126 MHz, $CDCl_3$) δ 163.7 (d, *J* = 242.9 Hz), 151.9 (d, *J* = 9.8 Hz), 114.1 (d, *J* = 1.9 Hz), 108.4 (d, *J* = 19.8 Hz), 102.1 (d, *J* = 25.9 Hz), 83.9, 40.5, 24.8.

$^{13}C\{^1H\}$ NMR of 4k′ (126 MHz, $CDCl_3$) δ 163.7 (d, *J* = 242.9 Hz), 151.9 (d, *J* = 9.8 Hz), 114.1 (d, *J* = 1.9 Hz), 108.4 (d, *J* = 19.8 Hz), 102.1 (d, *J* = 25.9 Hz), 83.8 (m), 40.5, 24.8.

^{11}B NMR (160 MHz, $CDCl_3$) δ 30.59 (br s).

Borylation of fluorobenzene (4l)

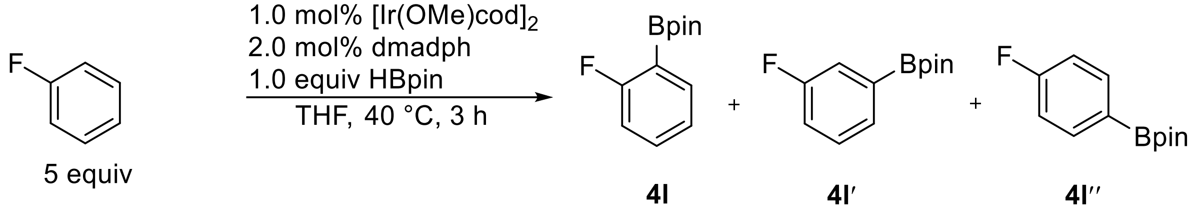

Reaction ran with 5-fold excess of substrate. Isolated as a colorless, clear liquid (**99%, 1.4 : 5.5 : 1.0 o:m:p, <5% diborylated isomers**) after column chromatography in 100% CH_2Cl_2. Conversion was determined from ^{19}F NMR to be 98% at 3 h. The NMR spectral data agree with previously reported literature.[6,9] The ^1H NMR resonances of the 2-monoborylated isomer and 4-monoborylated isomer were not assigned due to significant overlap with each other and the major isomer.

Crude selectivity via ^{19}F NMR was found to be 1.3:5.4:1.0 o:m:p to F.

The selectivity found via ^{19}F NMR for dtbpy was 3.4:5.2:1.0 o:m:p to F.

^1H NMR of 4l′ (500 MHz, $CDCl_3$) δ 7.56 (dd, *J* = 7.2, 0.5 Hz, 1H), 7.47 (dd, *J* = 9.2, 2.6 Hz, 1H), 7.33 (ddd, *J* = 13.3, 5.5, 2.6 Hz, 1H), 7.13 (m, 1H), 1.34 (s, 12H).

$^{13}C\{^1H\}$ NMR of 4l (126 MHz, $CDCl_3$) δ 167.2 (d, *J* = 250.9 Hz), 136.8 (d, *J* = 7.9 Hz), 133.3 (d, *J* = 8.8 Hz), 123.6 (d, *J* = 3.2 Hz), 115.2 (d, *J* = 23.9 Hz), 83.9, 24.8.

$^{13}C\{^1H\}$ NMR of 4l′ (126 MHz, $CDCl_3$) δ 162.5 (d, *J* = 246.4 Hz), 130.3 (d, *J* = 3.0 Hz), 129.5 (d, *J* = 7.1 Hz), 120.9 (d, *J* = 19.3 Hz), 118.2 (d, *J* = 21.0 Hz), 84.1, 24.8.

$^{13}C\{^1H\}$ NMR of 4l″ (126 MHz, $CDCl_3$) δ 165.1 (d, *J* = 250.2 Hz), 137.0 (d, *J* = 8.2 Hz), 114.8 (d, *J* = 20.2 Hz), 83.9, 24.8.

^{19}F NMR of 4l (470 MHz, $CDCl_3$) δ -102.66

^{19}F NMR of 4l′ (470 MHz, $CDCl_3$) δ -114.22

^{19}F NMR of 4l″ (470 MHz, $CDCl_3$) δ -108.45

^{11}B NMR (160 MHz, $CDCl_3$) δ 30.54 (br s)

Borylation of dimethyl resorcinol (5)

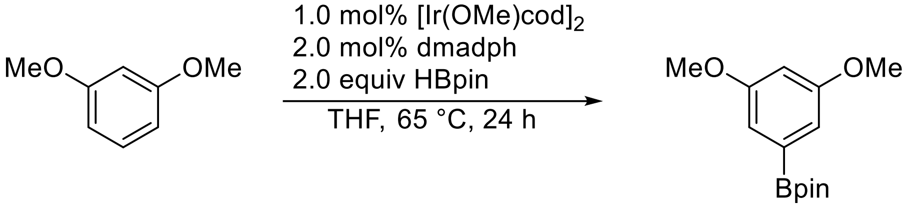

Reaction ran at 65 °C, and the conversion at 24 h was determined to be 72% by ^{1}H NMR.

Product is known[10] and was not isolated but assigned spectroscopically as the known product.

VI. Preparation of Ir^{I} hydrazido from dmadph and 6

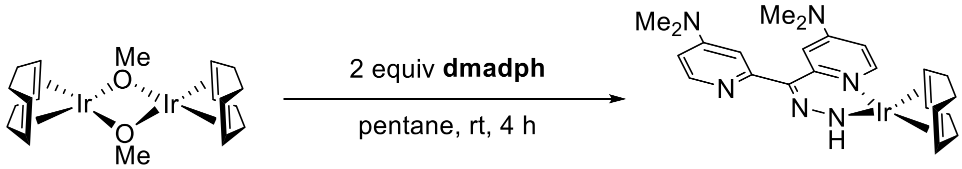

In a N_2 filled glovebox, a 20 mL scintillation vial was charged with $[Ir(OMe)cod]_2$ (0.033 g, 0.05 mmol) and a stir bar. In a small test tube, dmadph (0.028 g, 0.1 mmol) was weighed and suspended in 3.0 mL of pentane. Then, 2.0 mL of pentane was added to the scintillation vial with strong stirring yielding a light-yellow solution. The suspension of dmadph was then added with a pipette, changing the color immediately to a dark red-brown with significant precipitate. The mixture was allowed to stir for 4 hours at room temperature and placed in a freezer at -35 °C overnight. The resulting suspension was filtered over a glass frit funnel (F porosity) and washed with 3x5 mL portions of pentane and dried, yielding a dark maroon solid (0.030 g, **99%** yield) of the Ir^{I} hydrazido **7**.

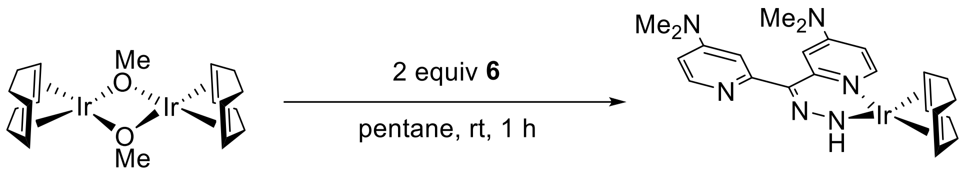

In a N_2 filled glovebox, a 20 mL scintillation vial was charged with $[Ir(OMe)cod]_2$ (0.033 g, 0.05 mmol) and a stir bar. In a small test tube, **6** (0.040 g, 0.1 mmol) was weighed and suspended in 3.0 mL of pentane. Then, 2.0 mL of pentane was added to the scintillation vial with strong stirring yielding a light-yellow solution. The suspension of **6** was then added with a pipette, changing the color immediately to a dark red-brown. The mixture was allowed to stir for 1 hour at room temperature and placed in a freezer at -35 °C overnight. The resulting suspension was filtered over a glass frit funnel (F porosity) and washed with 3x5 mL portions of pentane and dried, yielding a dark maroon solid (0.028 g, **93%** yield) of the Ir^{I} hydrazido **7**.

^{1}H NMR (500 MHz, THF-*d*$_8$) δ 9.23 (s, 1H), 8.12 (d, J = 5.7 Hz, 1H), 7.82 (d, J = 7.3 Hz, 1H), 7.49 (d, J = 3.1 Hz, 1H), 6.94 (d, J = 2.6 Hz, 1H), 6.45 (dd, J = 5.8, 2.6 Hz, 1H), 6.40 (dd, J = 7.3, 3.1 Hz, 1H), 3.69 – 3.60 (m, 2H), 3.51 – 3.44 (m, 2H), 3.02 (s, 6H), 2.96 (s, 6H), 2.44 – 2.29 (m, 4H), 2.32 – 2.16 (m, 4H).

^{13}C{^{1}H} NMR (126 MHz, THF-*d*$_8$) δ 171.3, 161.5, 154.9, 153.2, 147.6, 147.4, 107.7, 105.7, 104.2, 103.7, 66.9, 62.8, 58.4, 38.2, 37.9, 31.7, 30.5, 24.8.

VII. Borylation of fluorochlorobenzene with 6 and 7

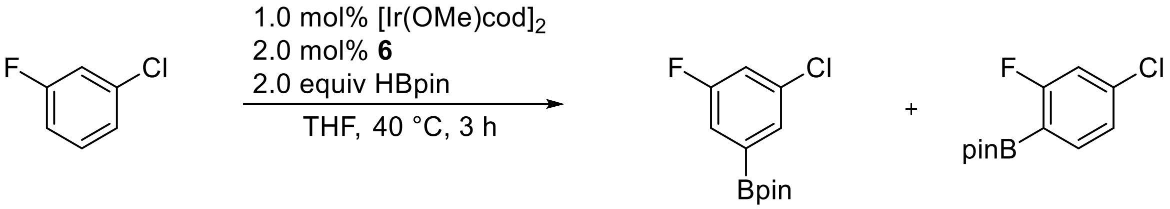

Reaction performed according to the general procedure with the following minor changes: **6** (0.008 g, 0.02 equiv) was weighed into a test tube in place of **L4**/dtbpy, and the reaction was stirred for 3 h. Conversion was found to be 80% by ^{19}F NMR analysis, with a regioisomeric ratio of 6.5:1.0 meta:ortho to F.

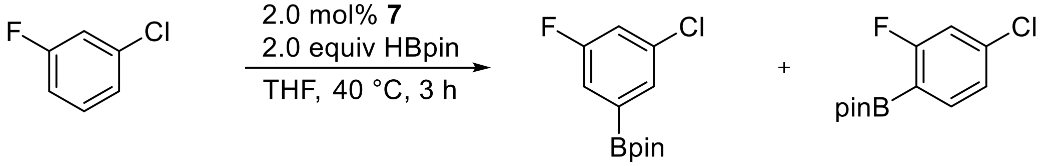

Reaction performed according to the general procedure with the following minor changes: **7** (0.012 g, 0.02 equiv) was weighed into a test tube in place of **L4**/dtbpy and [Ir(OMe)cod]$_2$, and the reaction was stirred at 40 °C for 3 h. Conversion was found to be 75% by ^{19}F NMR analysis, with a regioisomeric ratio of 6.5:1.0 meta:ortho to F.

VIII. Preparation of dmadph-borane adduct for X-ray crystallography

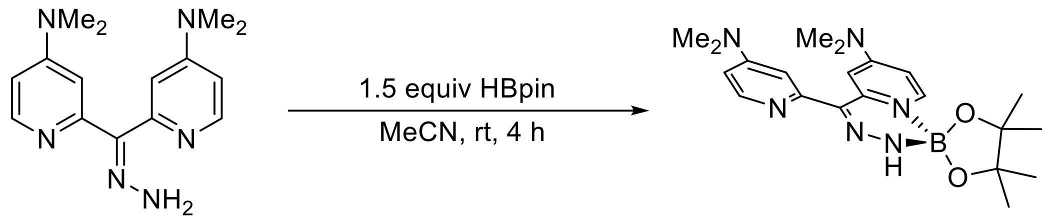

In a N_2 filled glovebox, a 20 mL scintillation vial was charged with a stir bar and dmadph (0.046 g, 0.16 mmol, 1 equiv). Then, 3.0 mL of MeCN was added to the vial and stirred. Afterwards, pinacolborane (0.40 mL, 0.28 mmol, 1.75 equiv) was added with a syringe and stirred for 4 h. The addition of pinacolborane turned the clear, colorless slurry to a light-yellow and caused the solution to warm and bubble. An additional 4.0 mL of MeCN was added to fully dissolve everything and was then stirred for another 20 h. After stirring the scintillation vial was placed in a -34 °C freezer for 2 days and upon returning, yellow needles precipitated (**0.051 g, 78%**) that were suitable for X-ray crystallography.

^{1}H NMR (500 MHz, CDCl$_3$) δ 8.42 (d, J = 7.2 Hz, 1H), 8.20 (d, J = 5.8 Hz, 1H), 7.52 (d, J = 2.4 Hz, 1H), 7.33 (br s, 1 H), 7.04 (d, J = 2.2 Hz, 1H), 6.63 (dd, J = 7.1, 2.4 Hz, 1H), 6.48 (dd, J = 5.6, 2.2 Hz, 1H), 3.06 (s, 6 H), 3.04 (s, 6H), 1.25 (s, 6H), 1.20 (s, 6H).

^{13}C{^{1}H} NMR (126 MHz, CDCl$_3$) δ 157.1, 155.3, 154.9, 147.9, 142.9, 139.6, 105.8, 105.2, 105.1, 102.6 79.5, 39.4, 39.3, 26.8, 26.0.

^{11}B NMR (160 MHz, CDCl$_3$) δ 2.92 (s, $\omega_{1/2}$ = 49 Hz)

HRMS (ESI+) *m/z* calculated for $C_{21}H_{32}N_6O_2B^+$ ([MH]$^+$) 411.2680, found *m/z* 411.2698.

IX. NMR Tube Borylation of Pentafluorobenzene

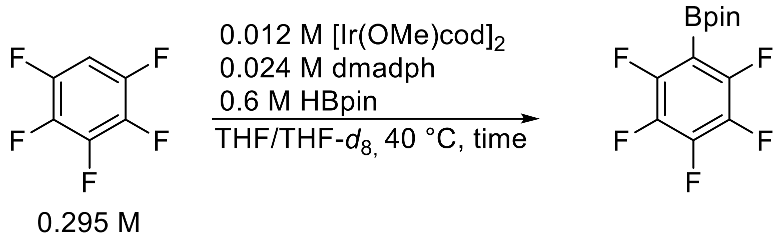

In a N_2 filled glovebox, $[Ir(OMe)cod]_2$ (0.0070 g, 0.01 mmol), dmadph (0.0060 g, 0.02 mmol), were weighed into separate test tubes. Then, 1.0 mL of THF was added to both test tubes. To the solution of Ir, HBpin (300 µL, 2 mmol) was added and shaken briefly, turning the light-yellow solution into a golden-yellow. The solution of Ir and pinacolborane was then pipetted into the solution of dmadph and transferred into a scintillation vial with a stir bar. The combined solution was stirred vigorously for 5 minutes, turning the solution dark red. Using a microsyringe, 400 µL of the combined solution was added to a J-young tube, followed by pentafluorobenzene (20 µL, 0.18 mmol) and 0.3 mL of THF-d_8. The J-young was capped, inverted and thoroughly mixed. The tube was then transferred into a preheated oil bath at 40 °C and monitored by ^{1}H, ^{19}F, and ^{11}B NMR.

See **Figure S58** for the ^{11}B NMR of the reaction at t = 3.5 h. The resonance at δ 25.36 is typical for a N–B bond where boron is 3-coordinate[11].

X. Crystallographic Data for 6

Slow crystallization of **6** from MeCN in a glovebox at –34 °C provided crystals suitable for X-ray diffraction analysis. Single yellow needle crystals of **6** used as received. A suitable crystal with dimensions 0.38 × 0.10 × 0.04 mm^3 was selected and mounted on a nylon loop with paratone oil on a Bruker APEX-II CCD diffractometer. The crystal was kept at a steady T = 173(1) K during data collection. The structure was solved with the **ShelXT**[12] solution program using dual methods and by using **Olex2**[13] as the graphical interface. The model was refined with **ShelXL**[14] using full matrix least squares minimisation on $\boldsymbol{F^2}$.

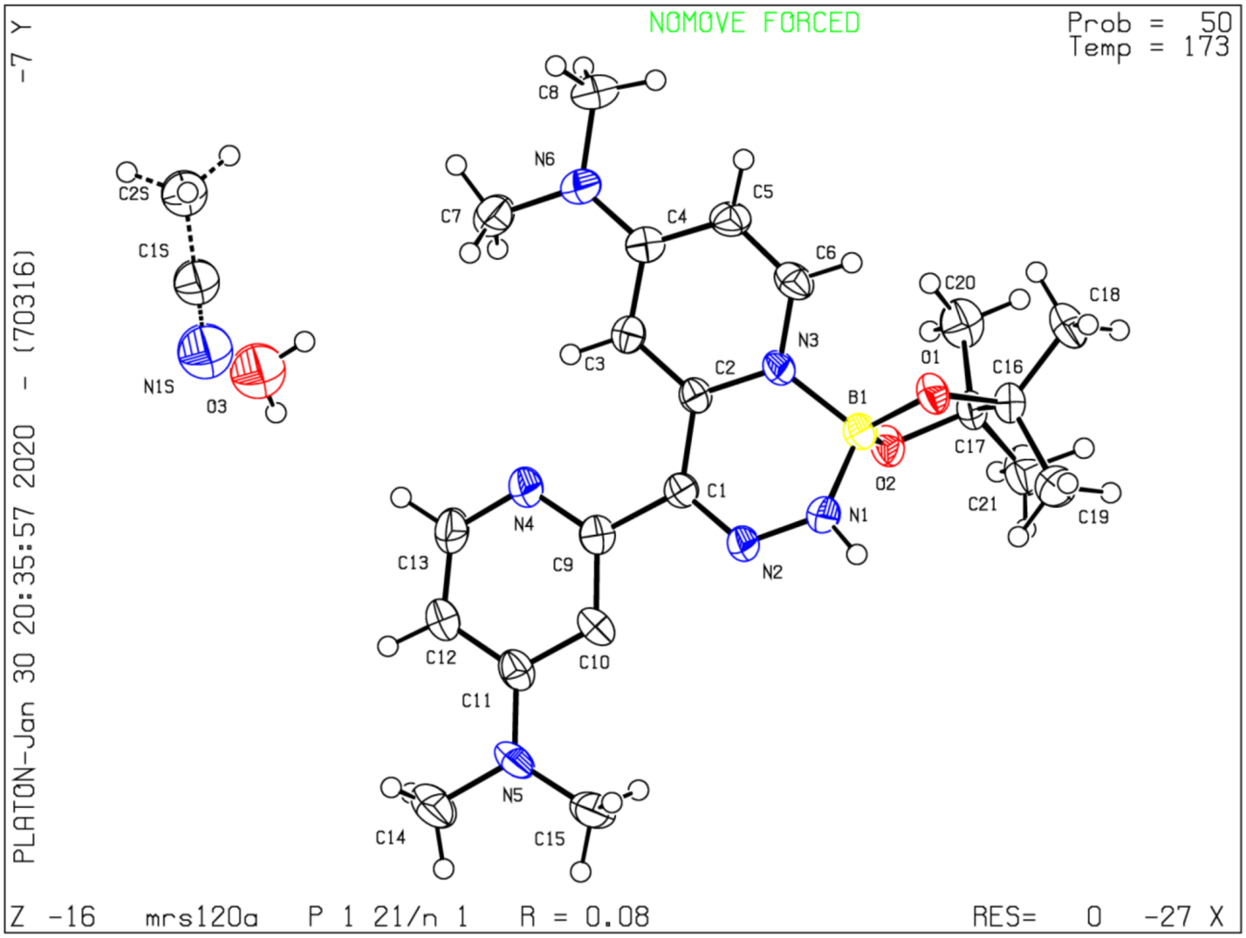

Figure S1. Representation of the solid-state structure of **6** at 50% probability ellipsoids. Co-crystallized H_2O and CH_3CN are shown.

Compound	6
CCDC	1981016
Formula	$C_{21.4}H_{31.8}BN_{6.2}O_{2.6}$
$D_{calc.}$/ g cm^{-3}	1.236
μ/mm^{-1}	0.669
Formula Weight	428.34
Colour	yellow
Shape	needle
Size/mm^3	0.38×0.10×0.04
T/K	173(1)
Crystal System	monoclinic
Space Group	$P2_1/n$
a/Å	13.9473(5)
b/Å	6.8875(3)
c/Å	24.6074(11)
α/°	90
β/°	103.062(3)
γ/°	90
V/Å^3	2302.67(17)
Z	4
Z'	1
Wavelength/Å	1.54178
Radiation type	CuK_α
Θ_{min}/°	3.688
Θ_{max}/°	68.228
Measured Refl's.	18482
Ind't Refl's	4193
Refl's with I > 2(I)	2066
R_{int}	0.1398
Parameters	295
Restraints	5
Largest Peak	0.557
Deepest Hole	-0.263
GooF	1.015
wR_2 (all data)	0.2476
wR_2	0.1965
R_1 (all data)	0.1665
R_1	0.0809

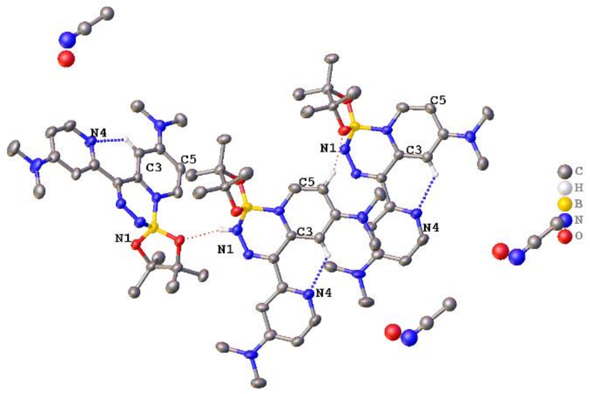

Figure S2: The following hydrogen bonding interactions with a maximum D-D distance of 3.2 Å and a minimum angle of 110 ° are present in **6**: N1–O1_*1: 3.158 Å, C3–N4: 2.843 Å, C5–O2_*2: 3.179 Å.

XI. References

(1) Uson, R.; Oro, L. A.; Cabeza, J. A.; Bryndza, H. E.; Stepro, M. P. Dinuclear Methoxy, Cyclooctadiene, and Barrelene Complexes of Rhodium(I) and Iridium(I): Kirschner/Inorganic. In *Inorganic Syntheses*; Kirschner, S., Ed.; Inorganic Syntheses; John Wiley & Sons, Inc.: Hoboken, NJ, USA, 1985; Vol. 231, pp 126–130.

(2) Pangborn, A. B.; Giardello, M. A.; Grubbs, R. H.; Rosen, R. K.; Timmers, F. J. Safe and Convenient Procedure for Solvent Purification. *Organometallics* **1996**, *15* (5), 1518–1520.

(3) Yeoh, K. K.; Chan, M. C.; Thalhammer, A.; Demetriades, M.; Chowdhury, R.; Tian, Y.-M.; Stolze, I.; McNeill, L. A.; Lee, M. K.; Woon, E. C. Y.; Mackeen, M. M.; Kawamura, A.; Ratcliffe, P. J.; Mecinović, J.; Schofield, C. J. Dual-Action Inhibitors of HIF Prolyl Hydroxylases That Induce Binding of a Second Iron Ion. *Org. Biomol. Chem.* **2013**, *11* (5), 732–745.

(4) Miller, S. L.; Chotana, G. A.; Fritz, J. A.; Chattopadhyay, B.; Maleczka, R. E., Jr; Smith, M. R., III. C-H Borylation Catalysts That Distinguish Between Similarly Sized Substituents Like Fluorine and Hydrogen. *Org. Lett.* **2019**, *21* (16), 6388–6392.

(5) Obligacion, J. V.; Bezdek, M. J.; Chirik, P. J. C(Sp2)-H Borylation of Fluorinated Arenes Using an Air-Stable Cobalt Precatalyst: Electronically Enhanced Site Selectivity Enables Synthetic Opportunities. *J. Am. Chem. Soc.* **2017**, *139* (7), 2825–2832.

(6) Ren, H.; Zhou, Y.-P.; Bai, Y.; Cui, C.; Driess, M. Cobalt-Catalyzed Regioselective Borylation of Arenes: N-Heterocyclic Silylene as an Electron Donor in the Metal-Mediated Activation of C−H Bonds. *Chem. Eur. J.* **2017**, *23* (24), 5663–5667.

(7) Kuleshova, O.; Asako, S.; Ilies, L. Ligand-Enabled, Iridium-Catalyzed Ortho-Borylation of Fluoroarenes. *ACS Catal.* **2021**, *11* (10), 5968–5973.

(8) Chotana, G. A.; Rak, M. A.; Smith, M. R., III. Sterically Directed Functionalization of Aromatic C-H Bonds: Selective Borylation Ortho to Cyano Groups in Arenes and Heterocycles. *J. Am. Chem. Soc.* **2005**, *127* (30), 10539–10544.

(9) Obligacion, J. V.; Semproni, S. P.; Chirik, P. J. Cobalt-Catalyzed C–H Borylation. *J. Am. Chem. Soc.* **2014**, *136* (11), 4133–4136.

(10) Wang, G.; Xu, L.; Li, P. Double N,B-Type Bidentate Boryl Ligands Enabling a Highly Active Iridium Catalyst for C–H Borylation. *J. Am. Chem. Soc.* **2015**, *137* (25), 8058–8061.

(11) Montero Bastidas, J. R.; Chhabra, A.; Feng, Y.; Oleskey, T. J.; Smith, M. R., III; Maleczka, R. E. Steric Shielding Effects Induced by Intramolecular C–H···O Hydrogen Bonding: Remote Borylation Directed by Bpin Groups. *ACS Catal.* **2022**, *12* (4), 2694–2705.

(12) Sheldrick, G. M. SHELXT - Integrated Space-Group and Crystal-Structure Determination. *Acta Crystallogr A Found Adv* **2015**, *71* (Pt 1), 3–8.

(13) Dolomanov, O. V.; Bourhis, L. J.; Gildea, R. J.; Howard, J. A. K.; Puschmann, H. OLEX2: A Complete Structure Solution, Refinement and Analysis Program. *J. Appl. Crystallogr.* **2009**, *42* (2), 339–341.

(14) Sheldrick, G. M. A Short History of SHELX. *Acta Crystallogr. A* **2008**, *64* (Pt 1), 112–122.

XII. NMR Spectra

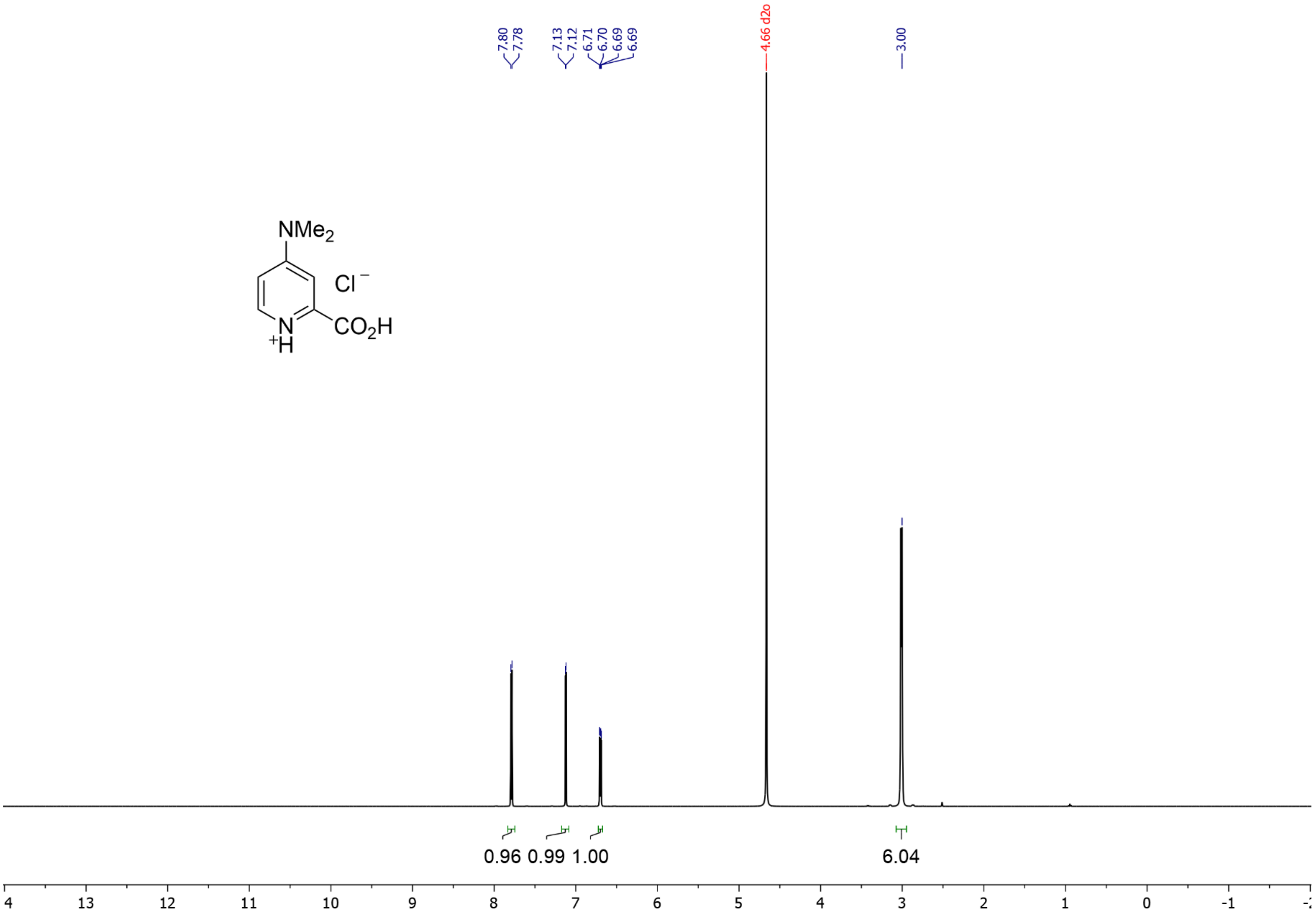

Figure S3. ^{1}H NMR of 4-(dimethylamino)picolinic acid hydrogen chloride (500 MHz, D_2O)

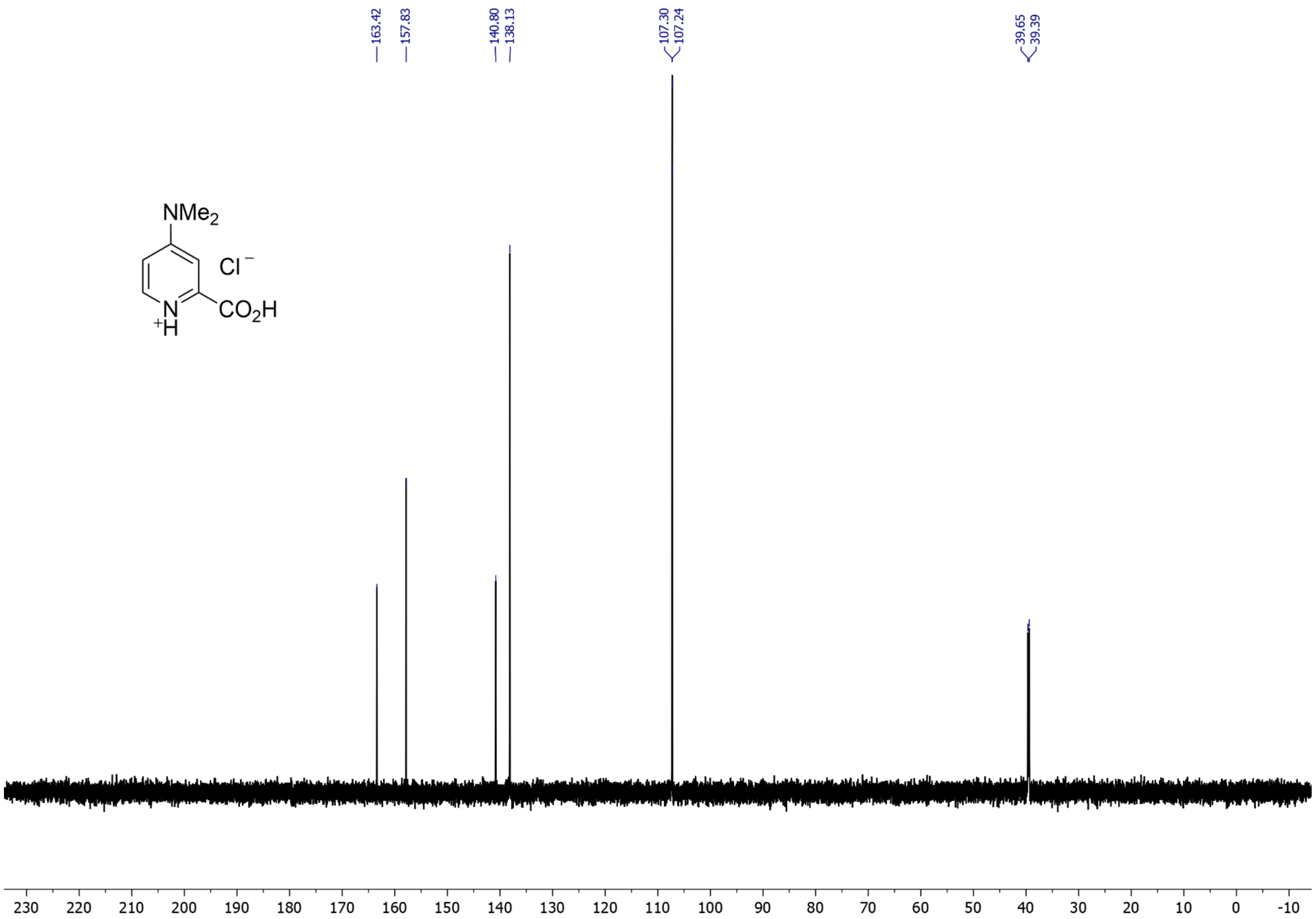

Figure S4. ^{13}C NMR of 4-(dimethylamino)picolinic acid hydrogen chloride (125 MHz, D_2O)

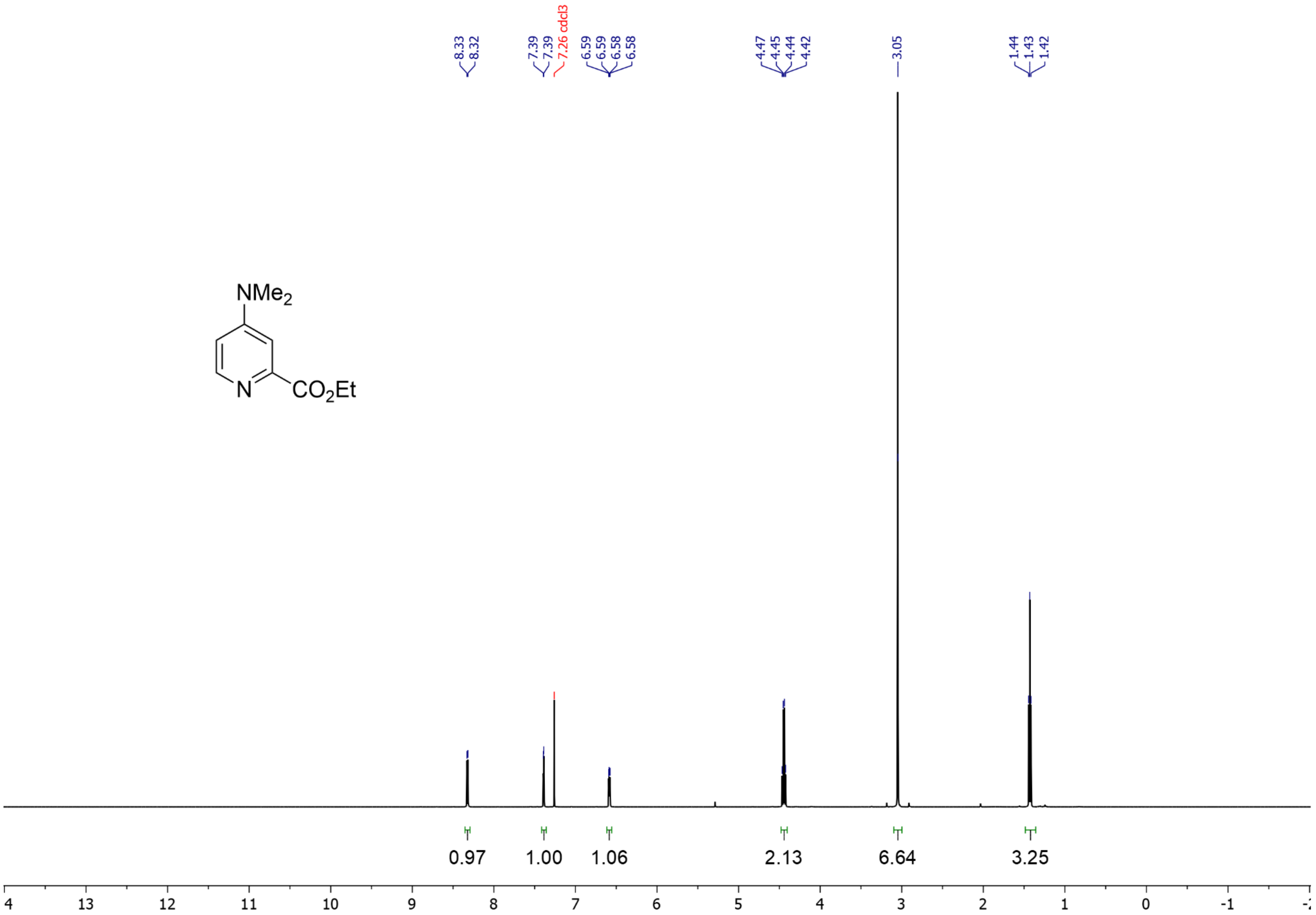

Figure S5 . ^{1}H NMR of ethyl-4-(dimethylamino)picolinate (500 MHz, $CDCl_3$)

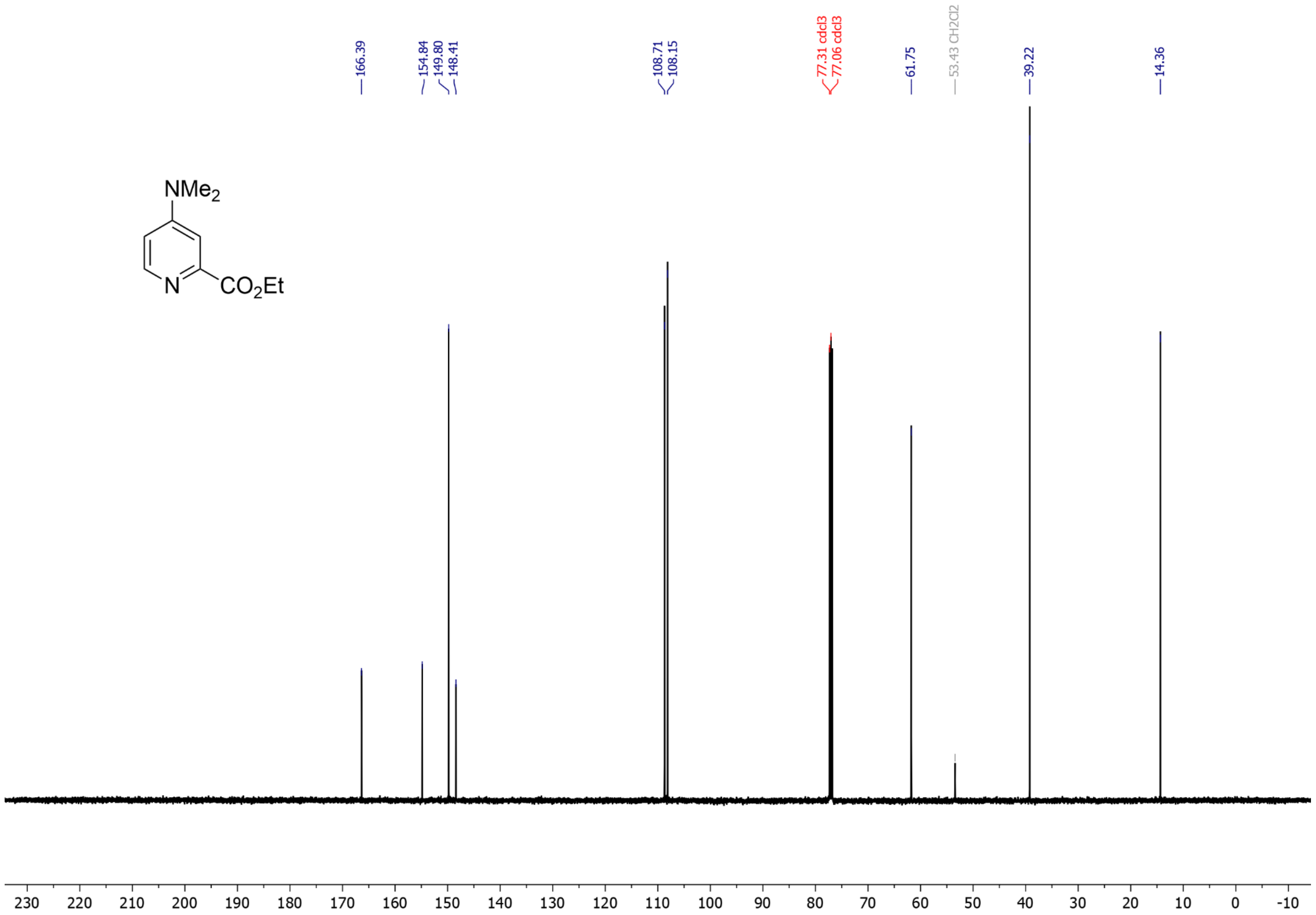

Figure S6. ^{13}C NMR of ethyl-4-(dimethylamino)picolinate (125 MHz, $CDCl_3$)

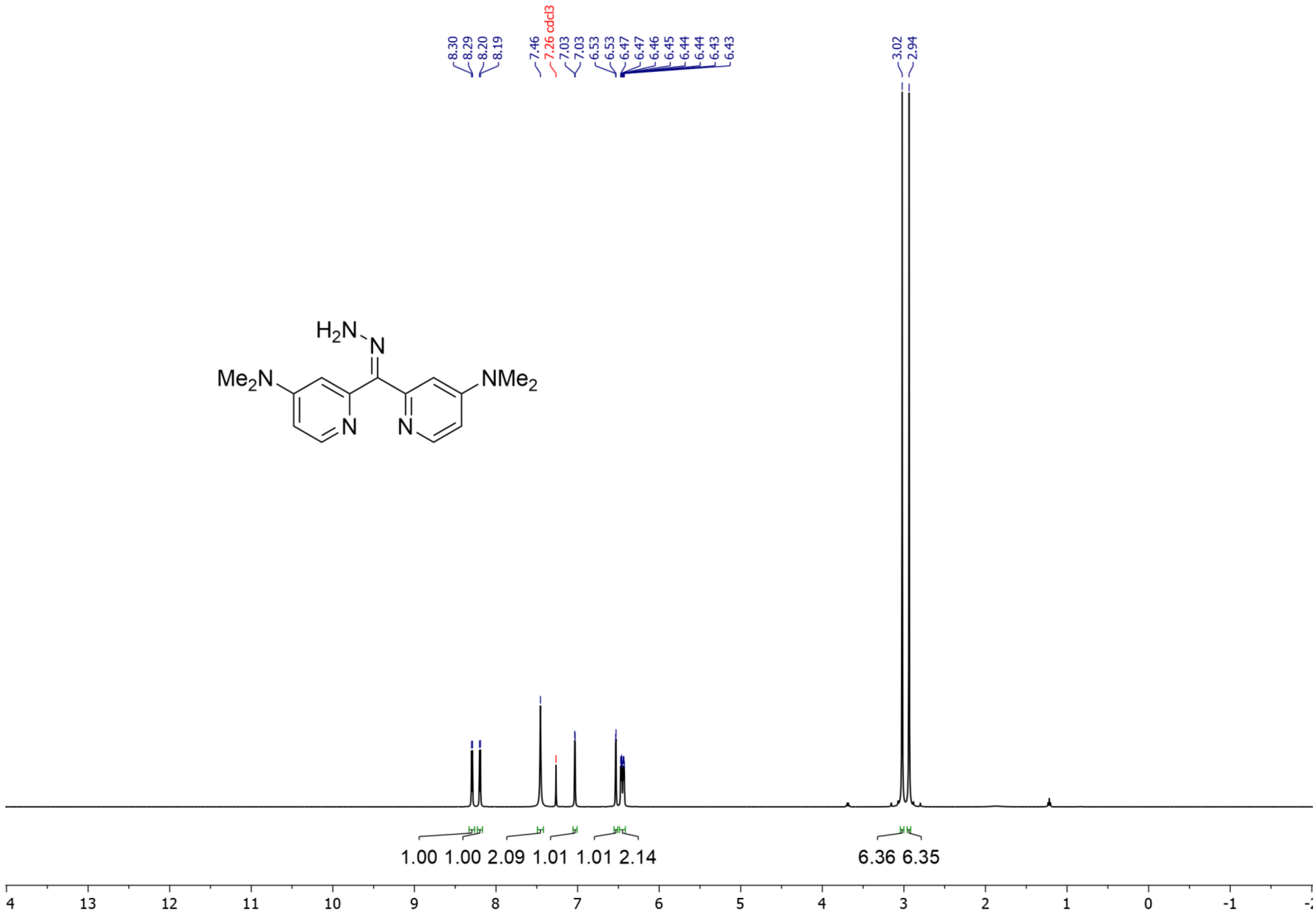

Figure S7. ^{1}H NMR of bis((4-dimethylamino)pyridine)methylene hydrazine (dmadph) (500 MHz, $CDCl_3$)

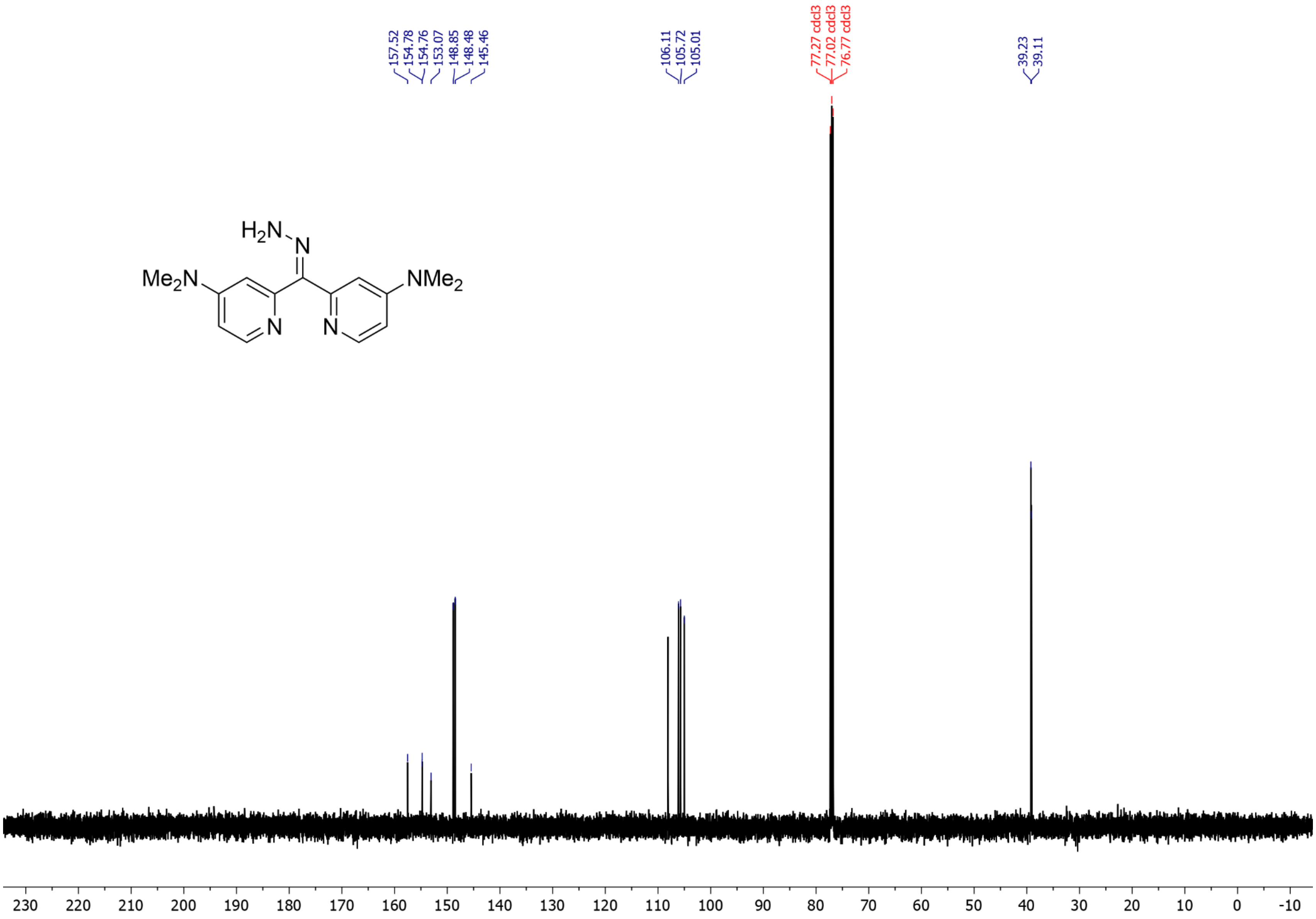

Figure S8. ^{13}C NMR of bis((4-dimethylamino)pyridine)methylene hydrazine (dmadph) 125 MHz, $CDCl_3$)

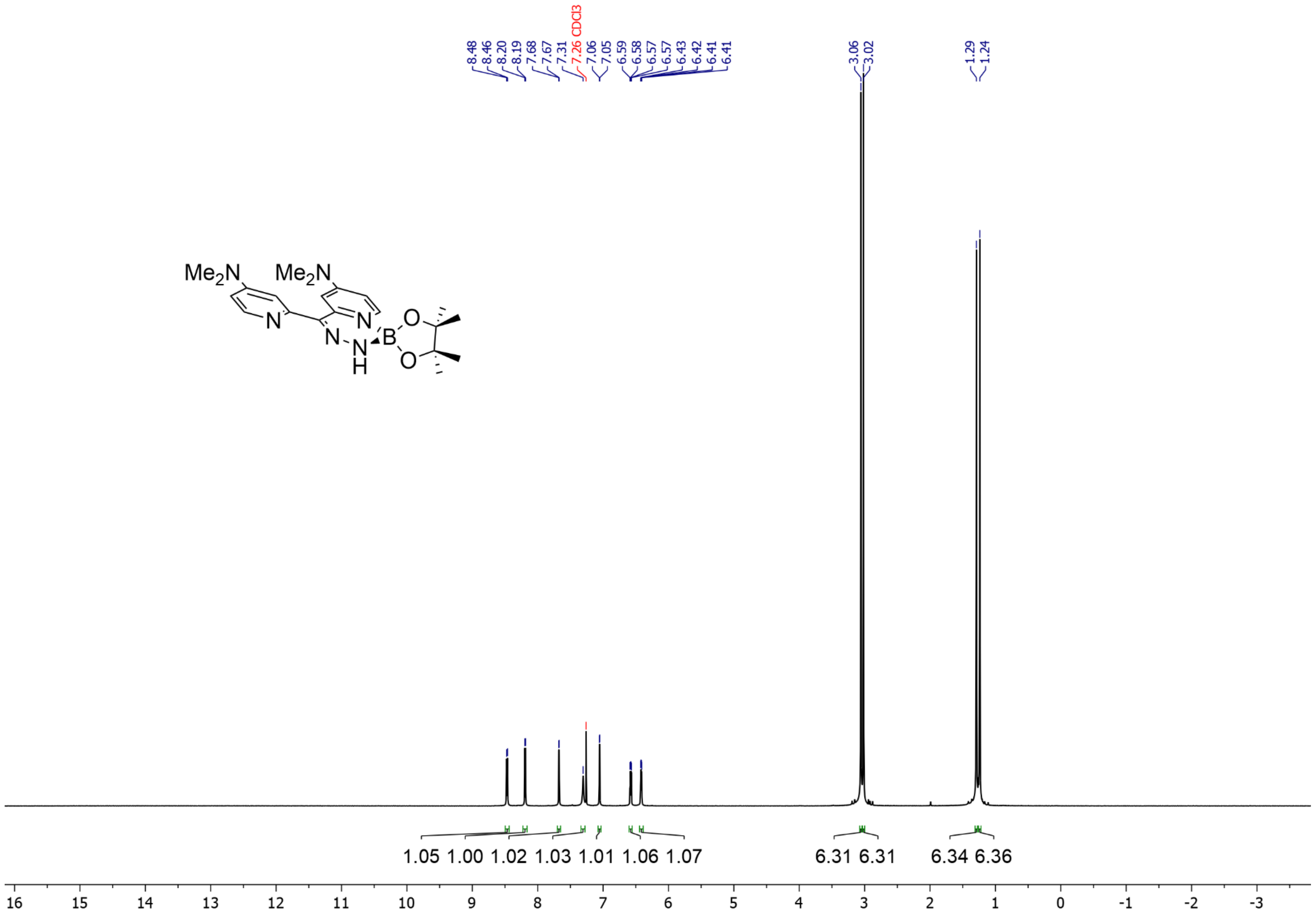

Figure S9. ^{1}H NMR of dmadph-HBpin adduct (500 MHz, $CDCl_3$)

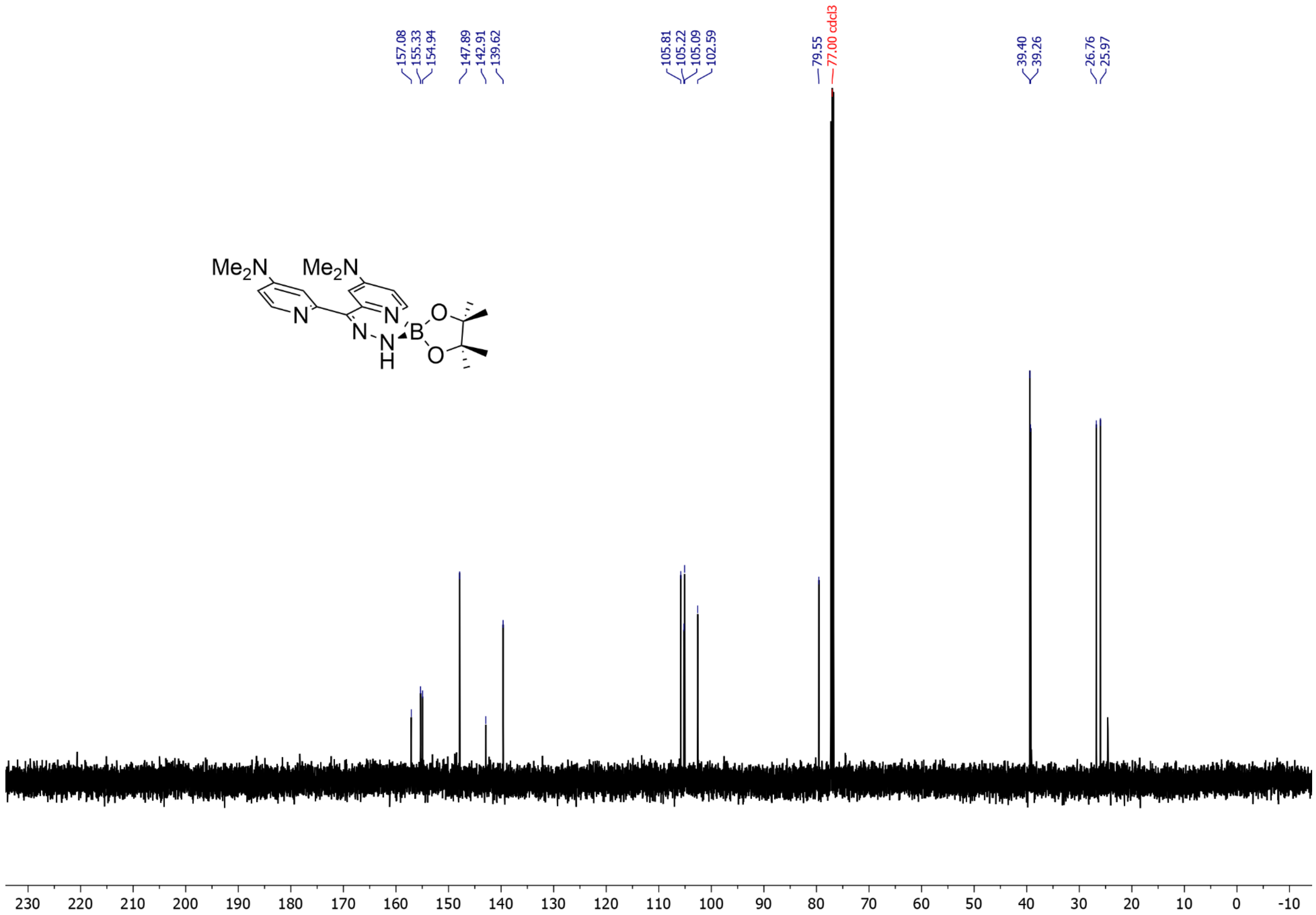

Figure S10. ^{13}C NMR of dmadph-HBpin adduct (126 MHz, $CDCl_3$)

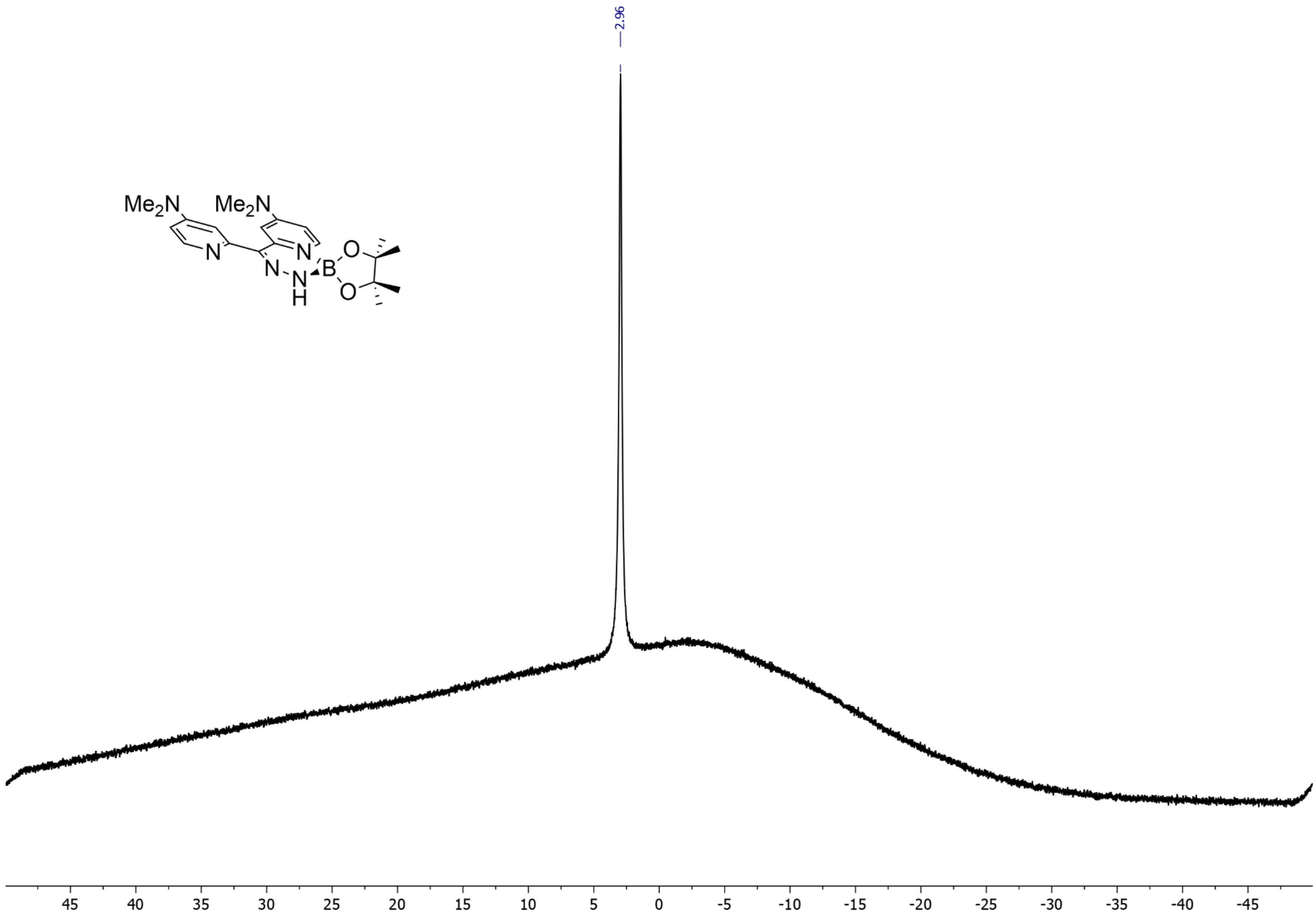

Figure S11. ^{11}B NMR of dmadph-HBpin adduct (160 MHz, $CDCl_3$)

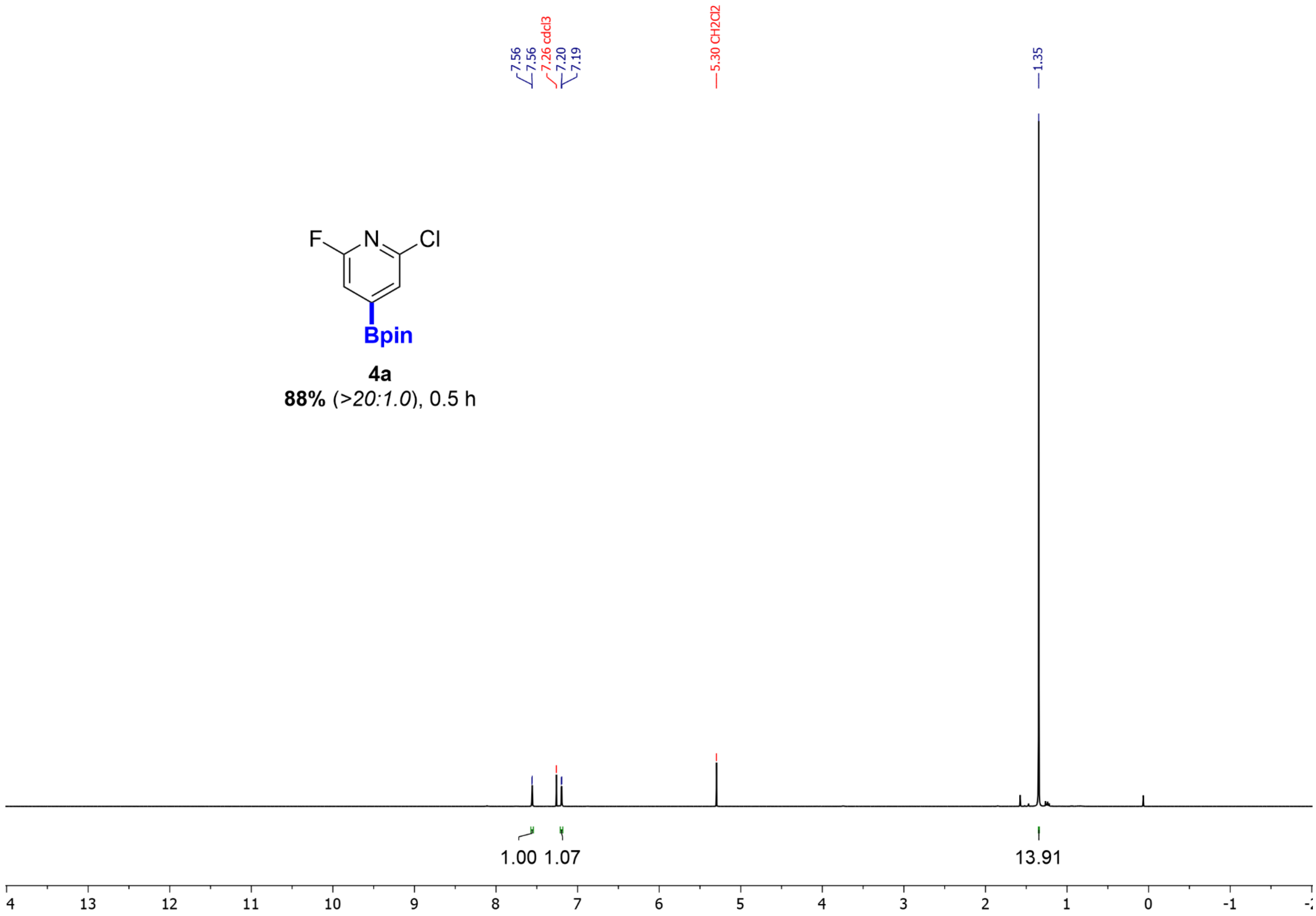

Figure S12. ^{1}H NMR of **4a** (500 MHz, $CDCl_3$)

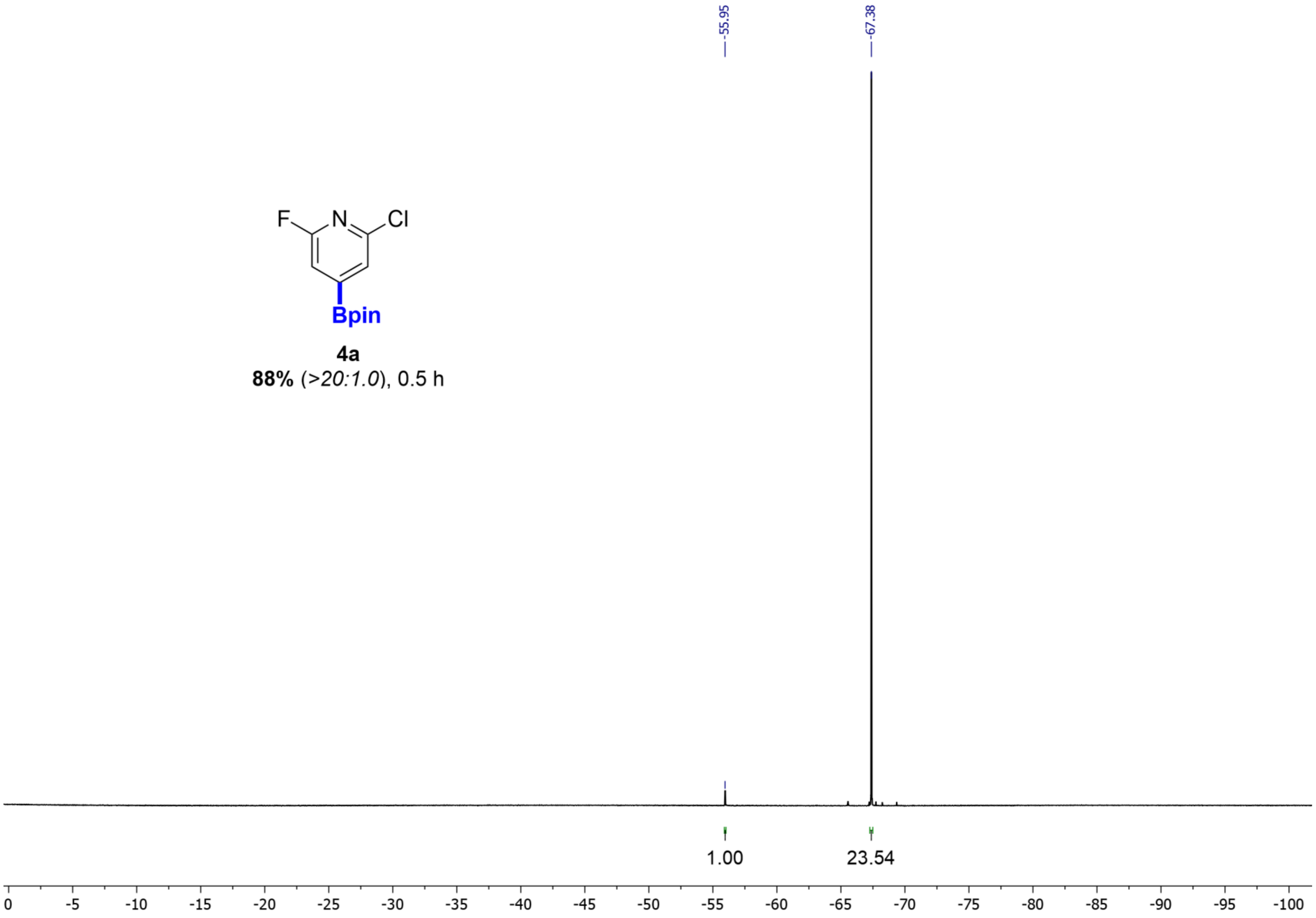

4a

88% (>*20:1.0*), 0.5 h

Figure S13. ^{19}F NMR of **4a** (470 MHz, $CDCl_3$)

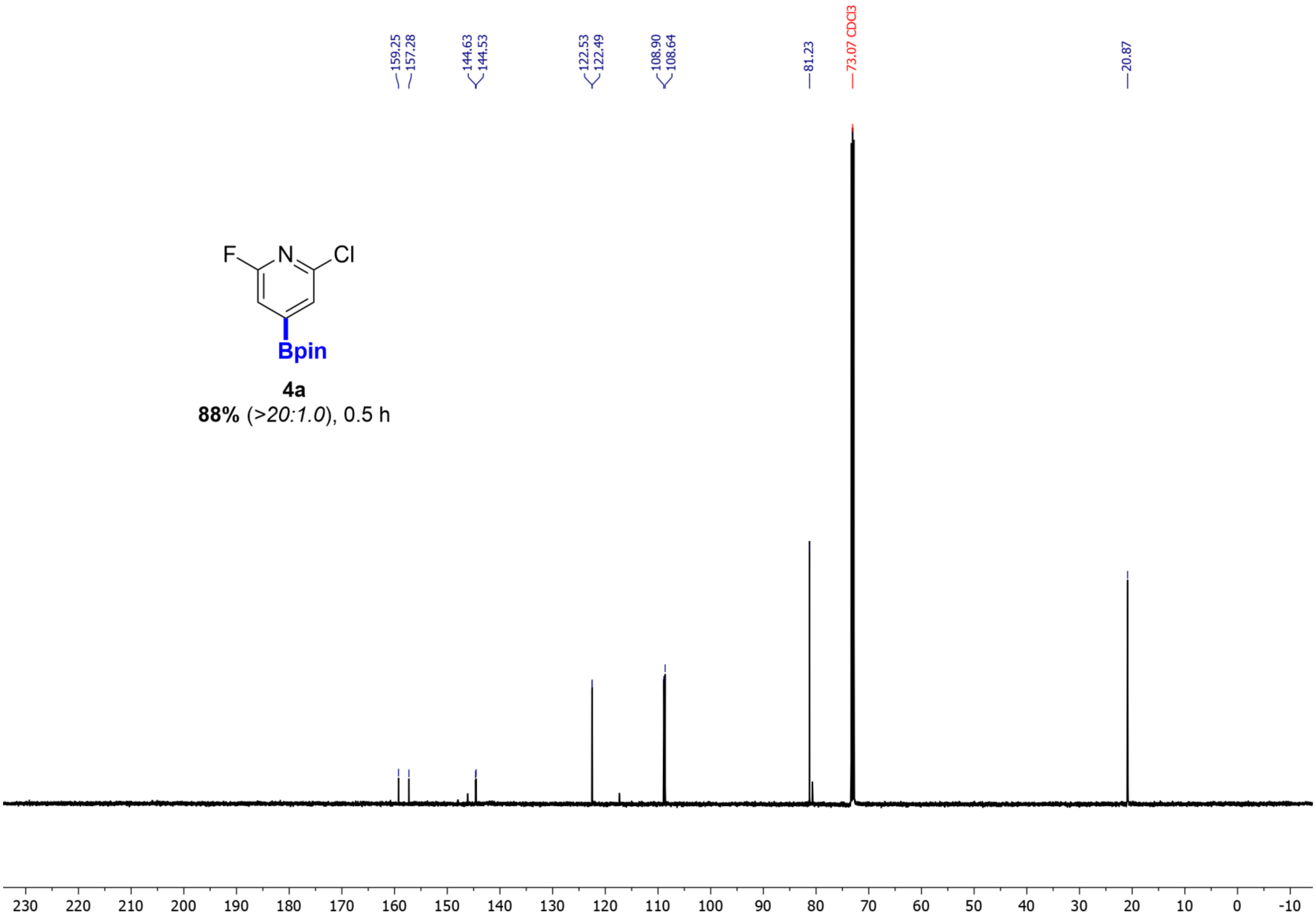

Figure S14. ^{13}C NMR of **4a** (126 MHz, $CDCl_3$)

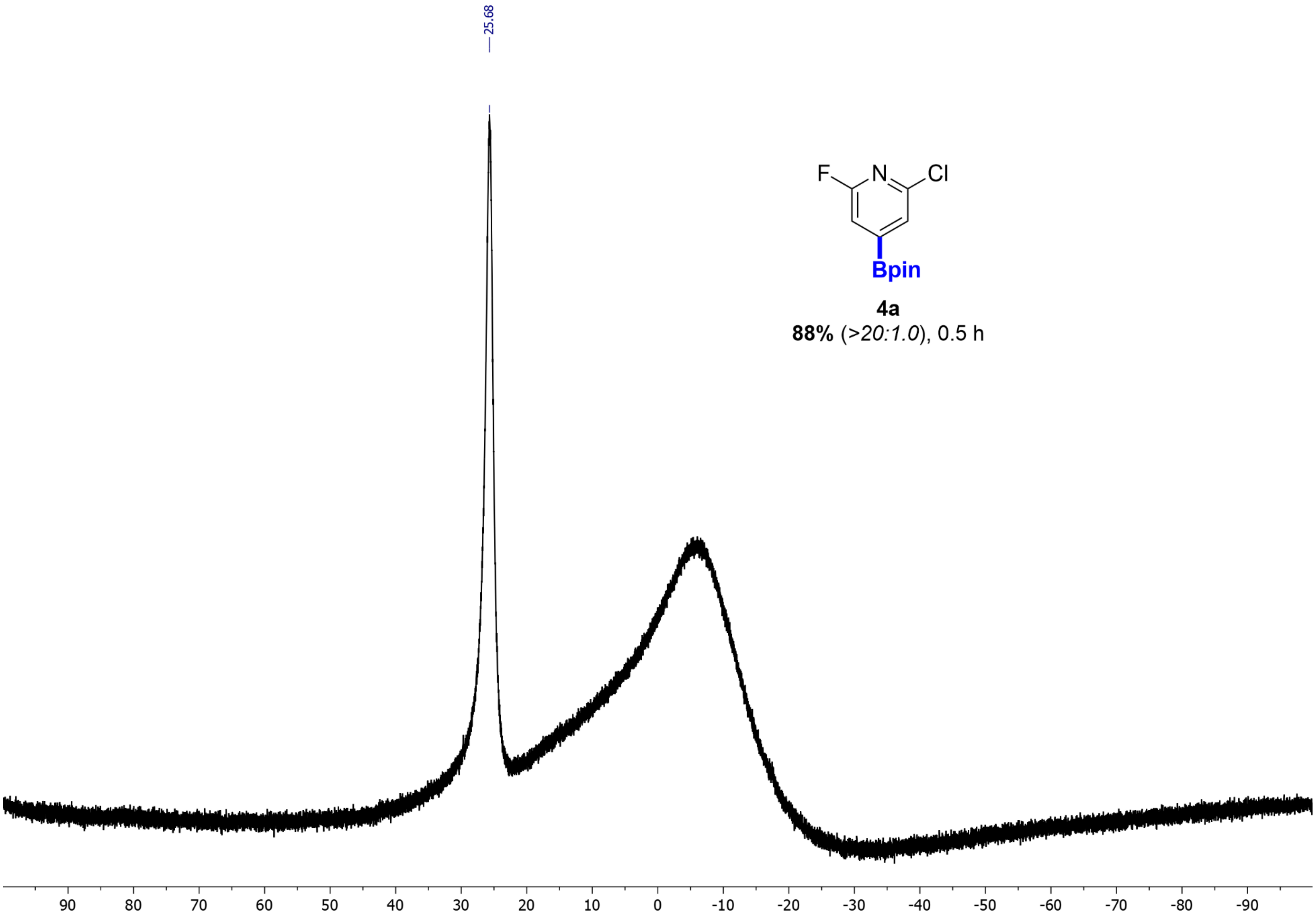

Figure S14. ^{11}B NMR of **4a** (160 MHz, $CDCl_3$)

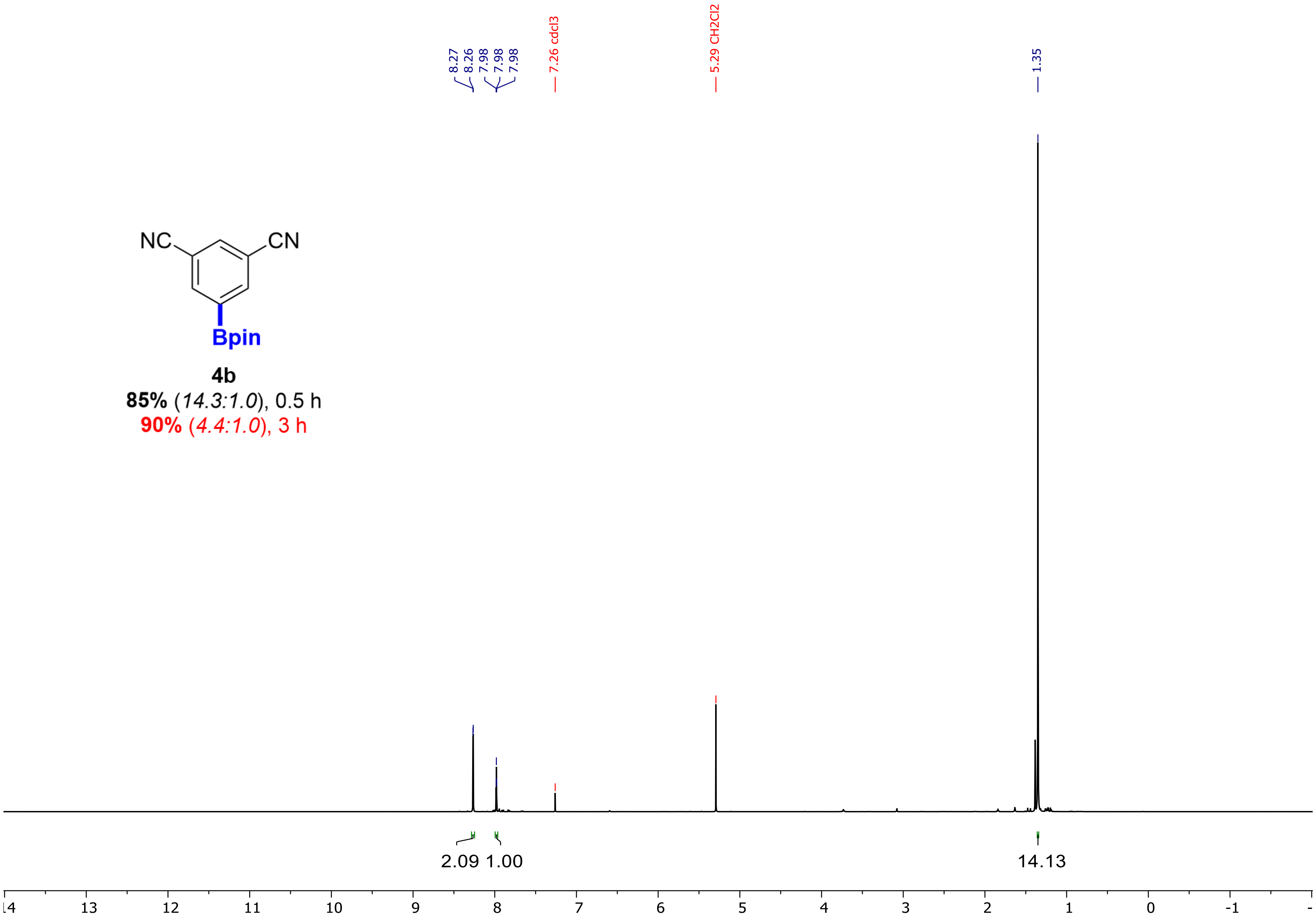

Figure S15. ^{1}H NMR of **4b** (160 MHz, $CDCl_3$)

4b
85% (*14.3:1.0*), 0.5 h
90% (*4.4:1.0*), 3 h

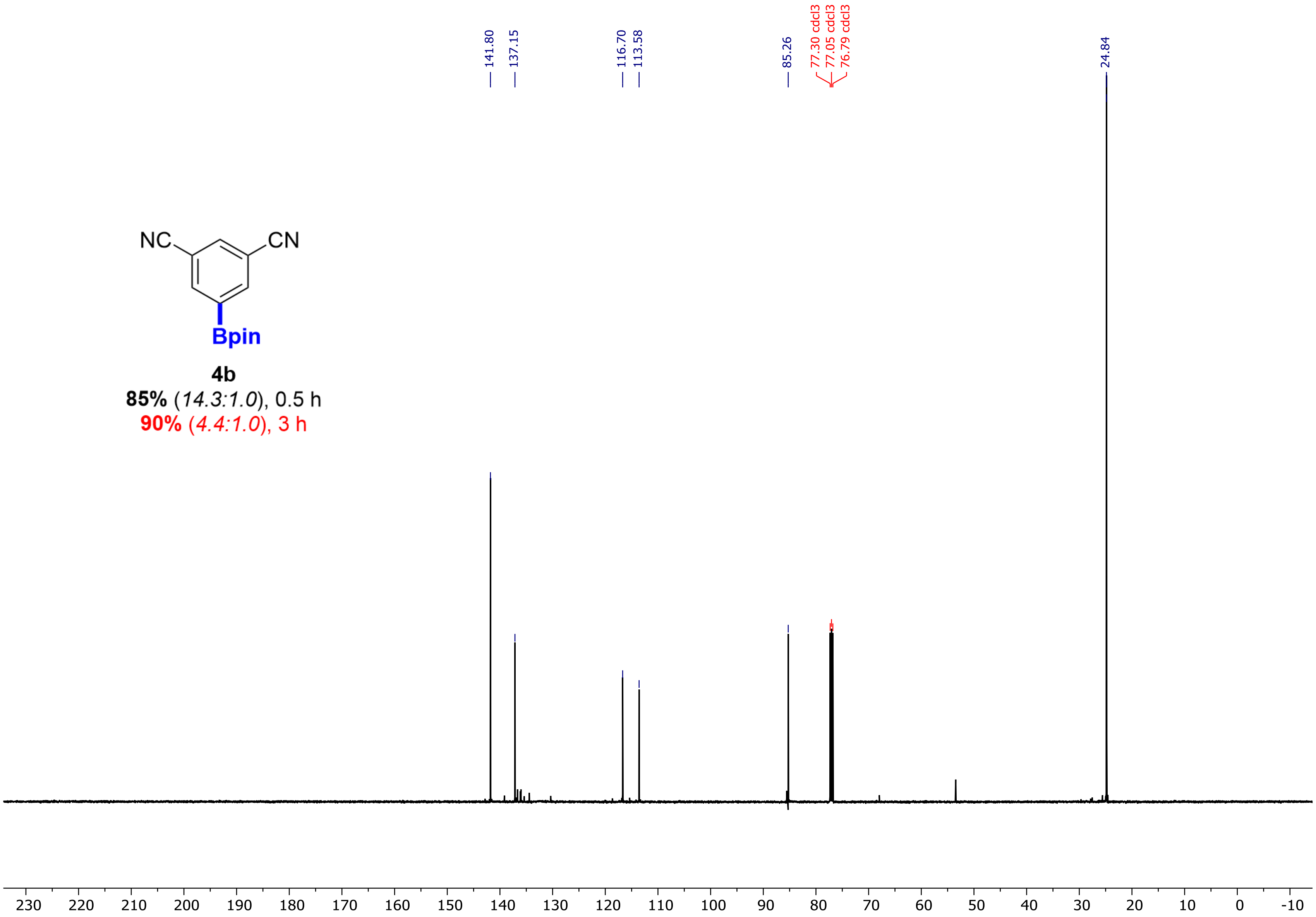

Figure S16. ^{13}C NMR of **4b** (126 MHz, $CDCl_3$)

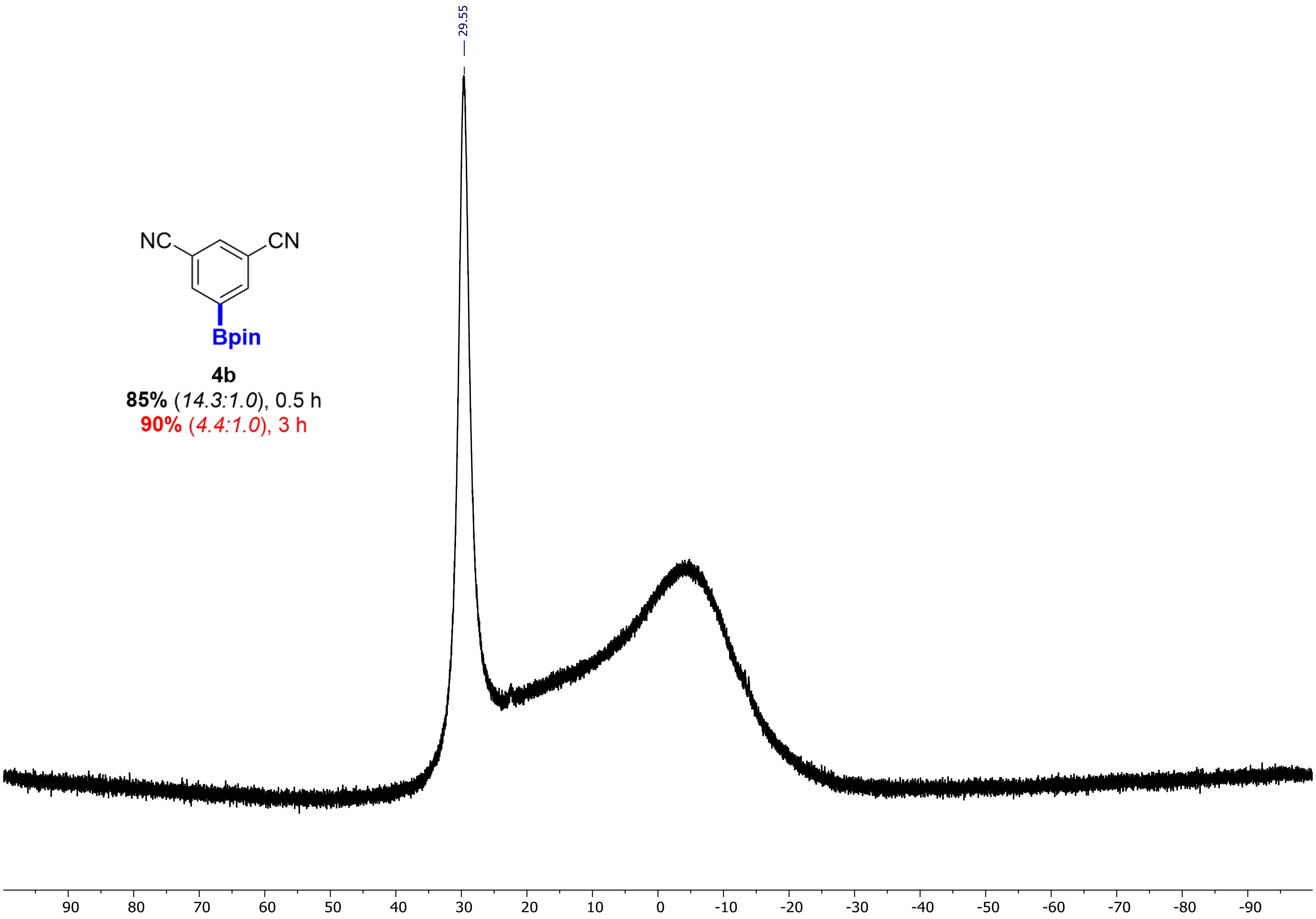

Figure S17. ^{11}B NMR of **4b** (160 MHz, $CDCl_3$)

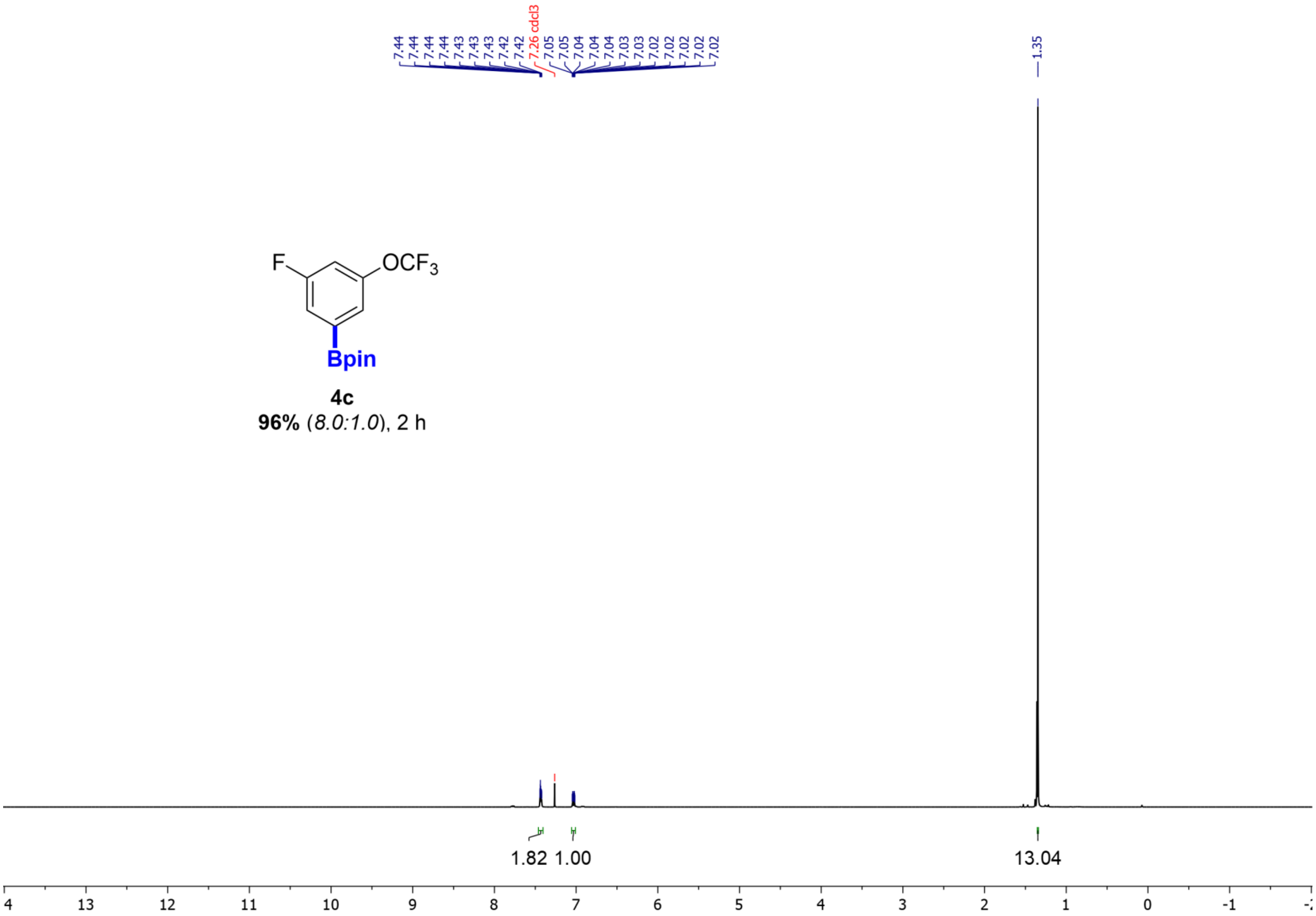

4c
96% (*8.0:1.0*), 2 h

Figure S18. ^{1}H NMR of **4c** (500 MHz, $CDCl_3$)

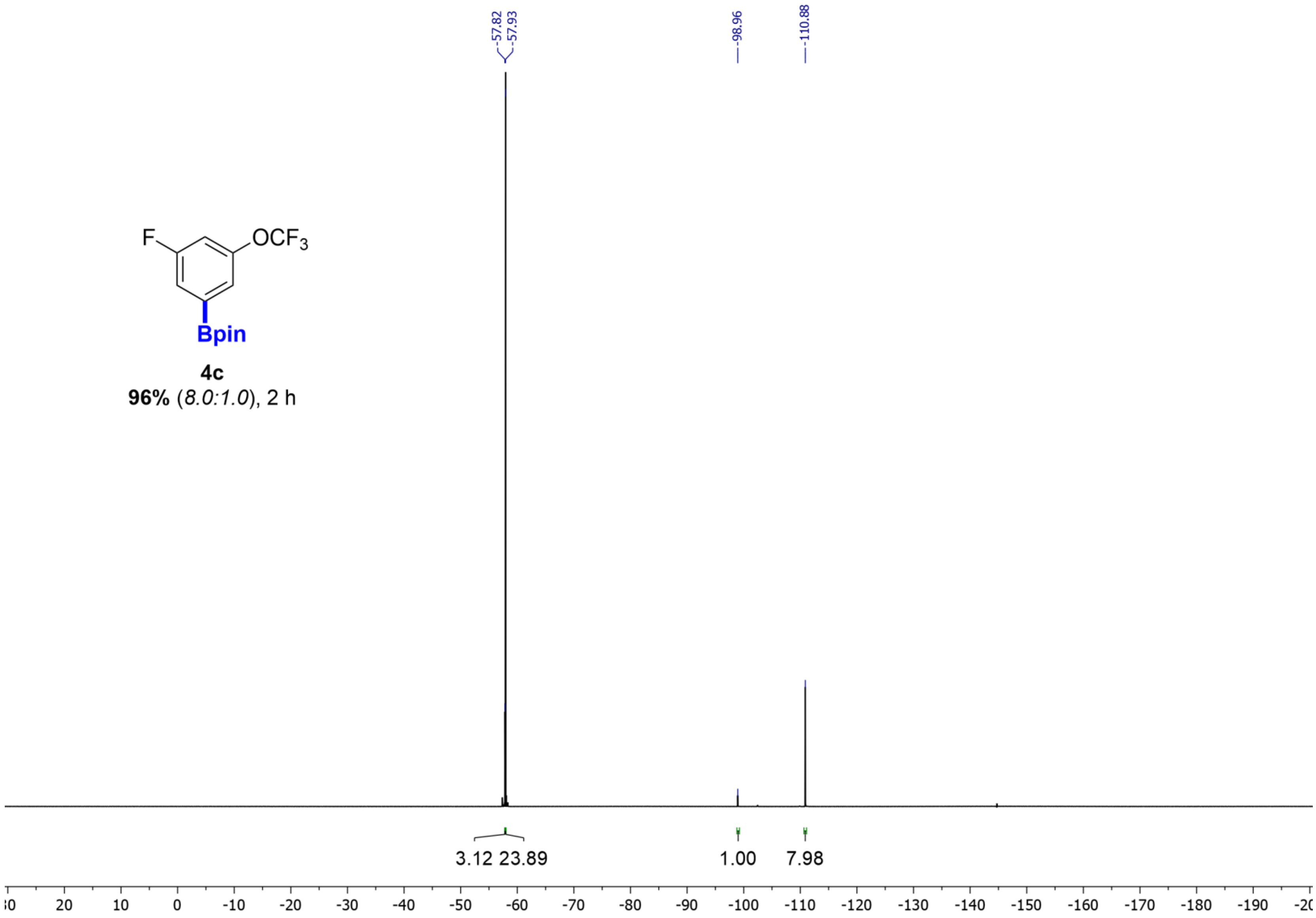

Figure S19. ^{19}F NMR of **4c** (470 MHz, $CDCl_3$)

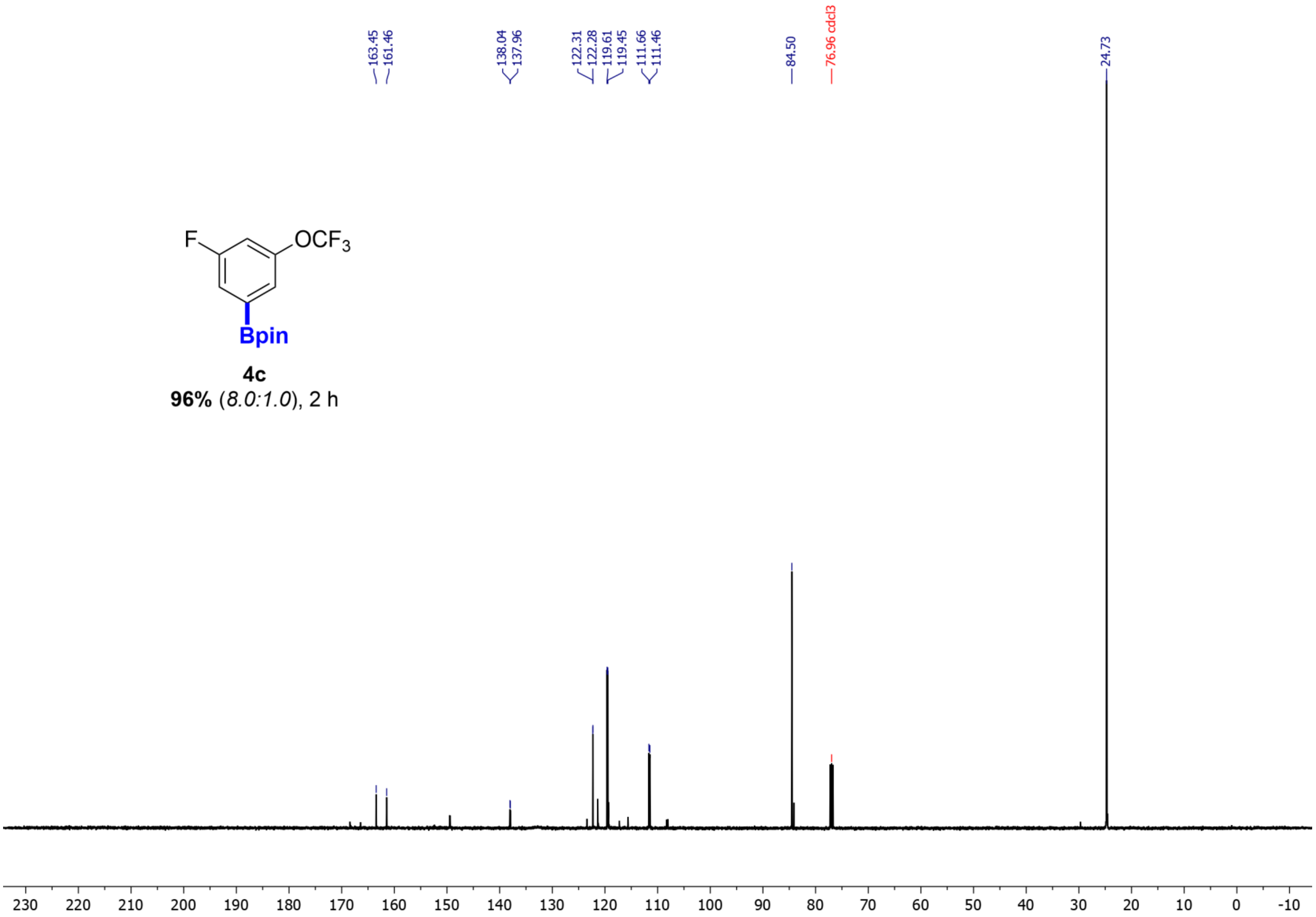

Figure S20. ^{13}C NMR of **4c** (126 MHz, $CDCl_3$)

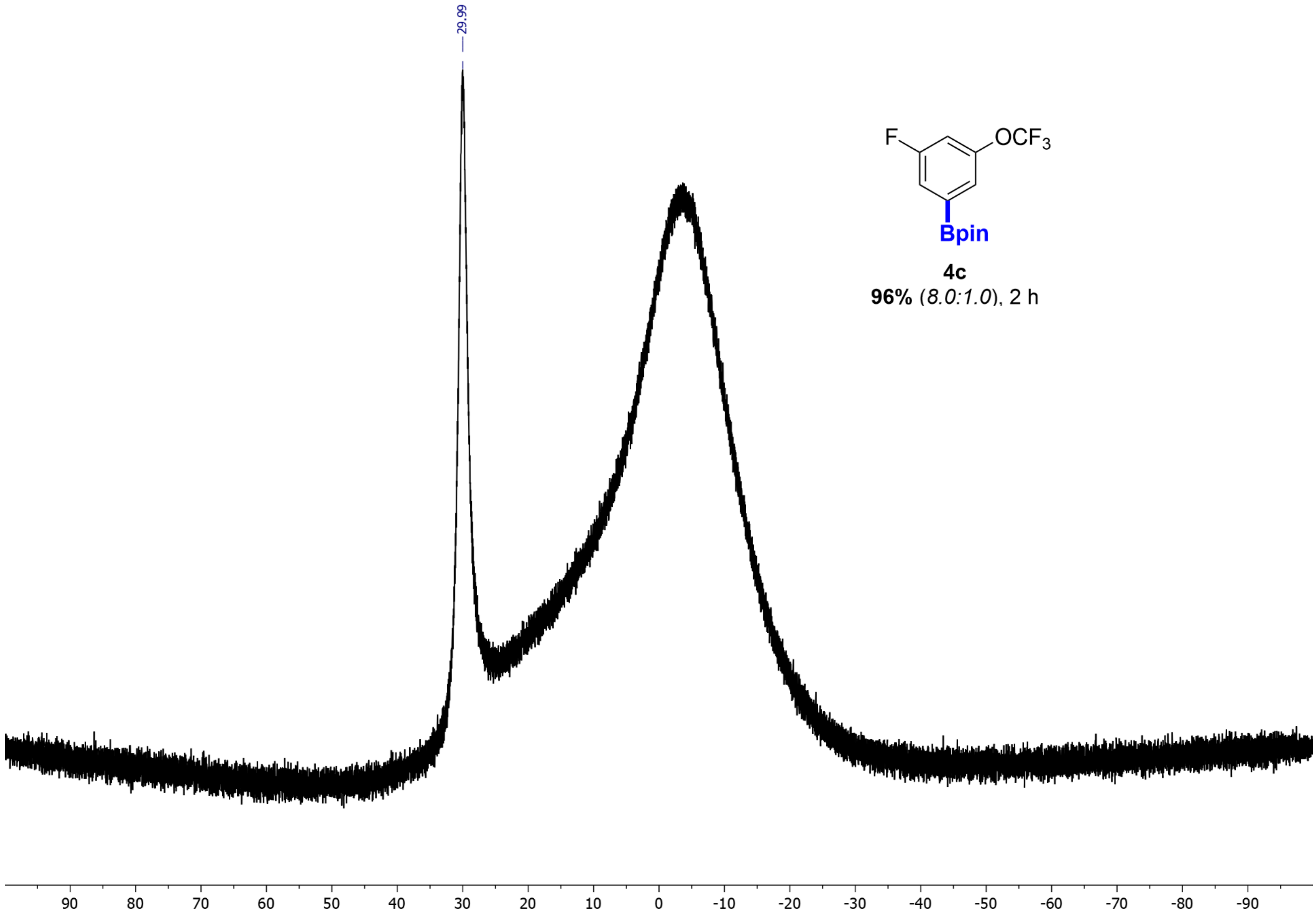

Figure S21. ^{11}B NMR of **4c** (160 MHz, $CDCl_3$)

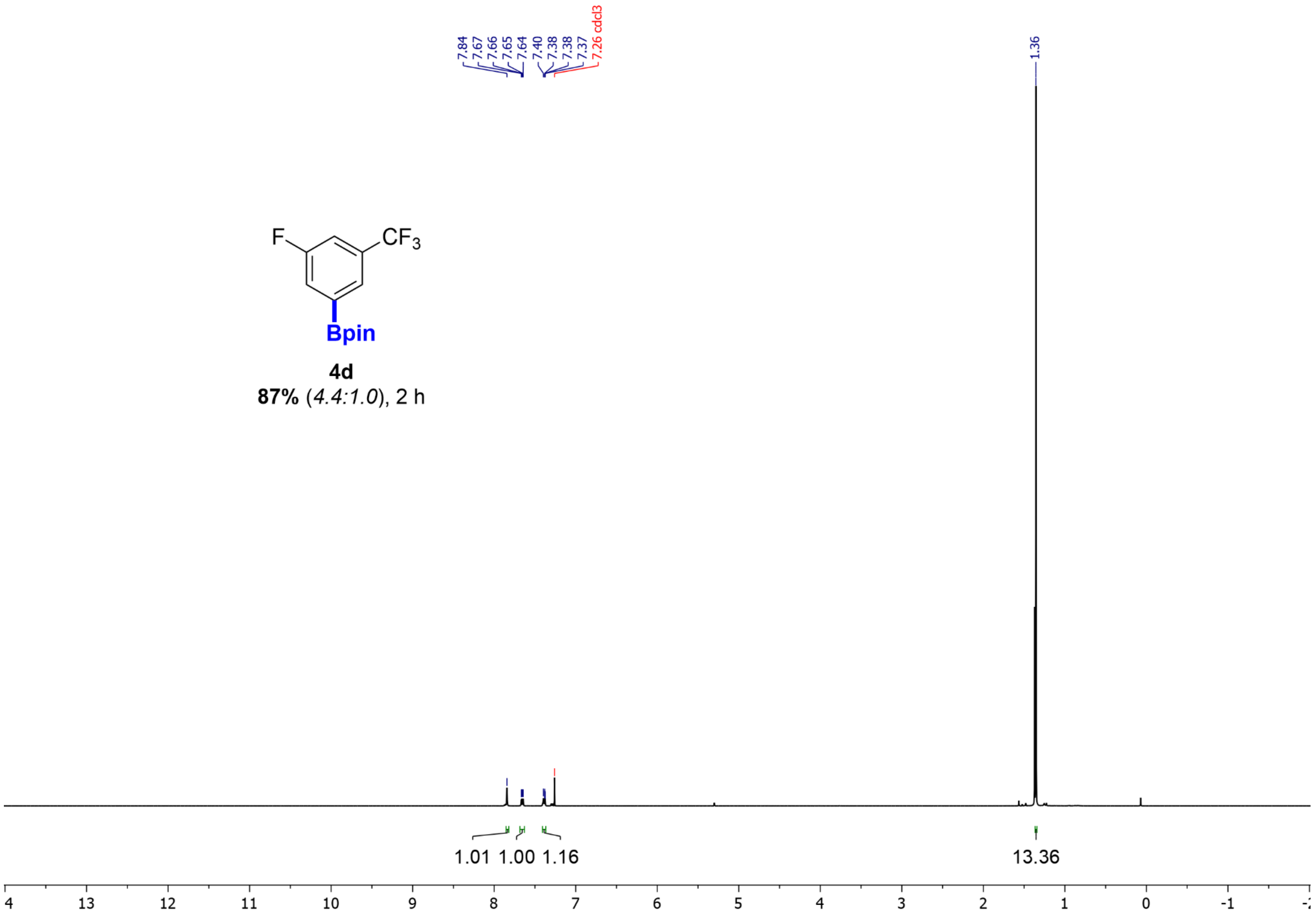

Figure S22. ^{1}H NMR of **4d** (500 MHz, $CDCl_3$)

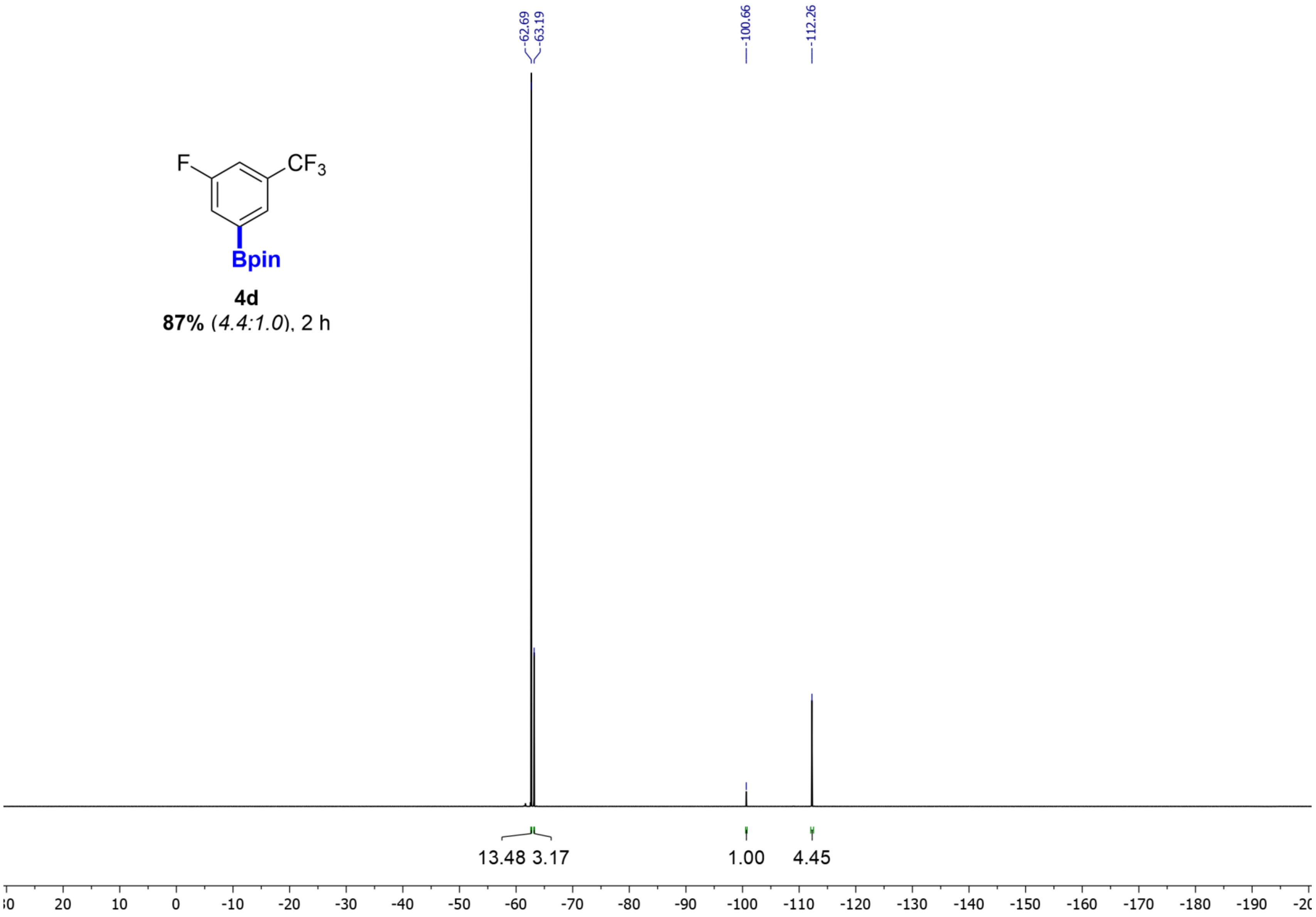

Figure S23. ^{19}F NMR of **4d** (470 MHz, $CDCl_3$)

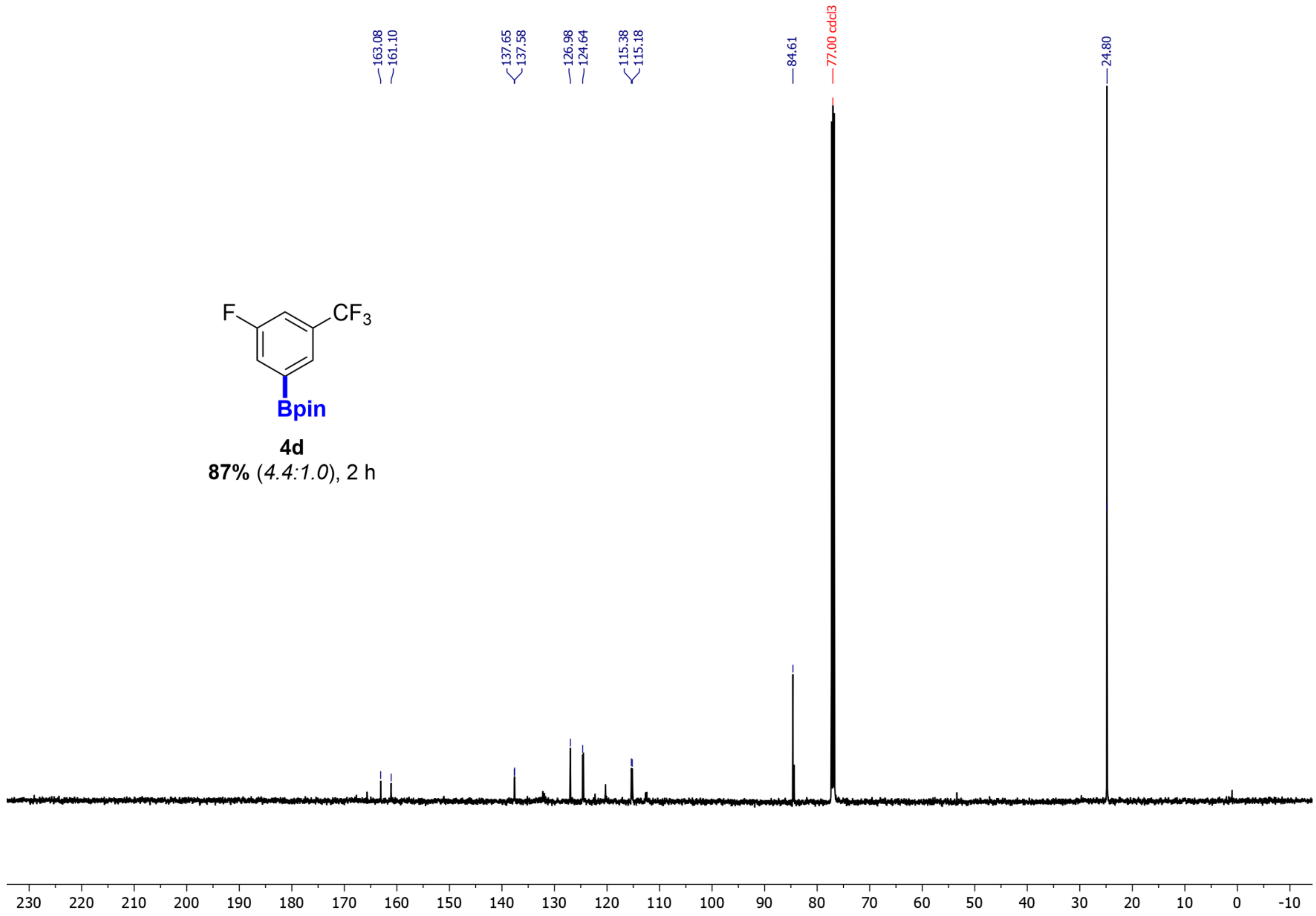

Figure S24. ^{13}C NMR of **4d** (126 MHz, $CDCl_3$)

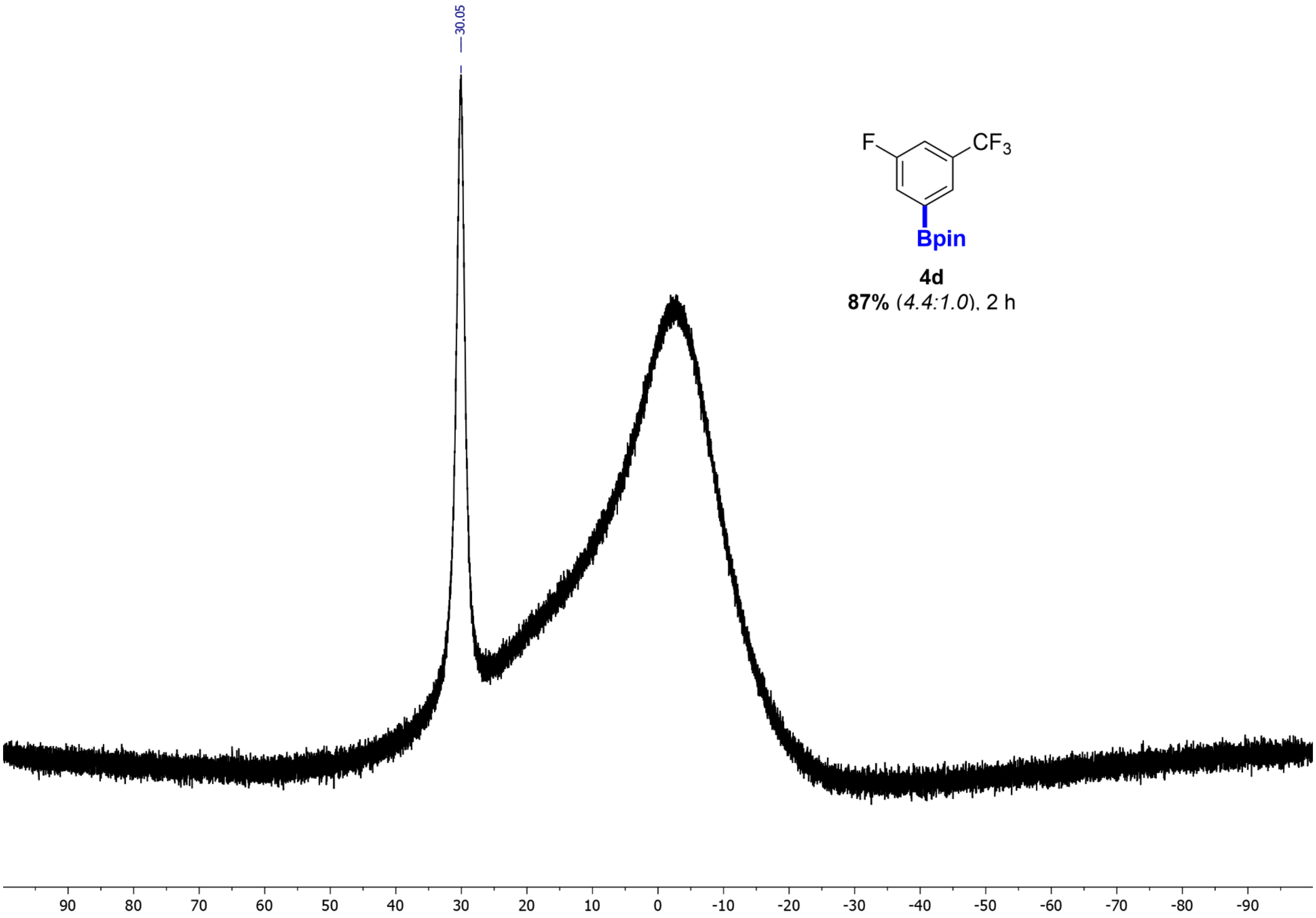

Figure S25. ^{11}B NMR of **4d** (160 MHz, $CDCl_3$)

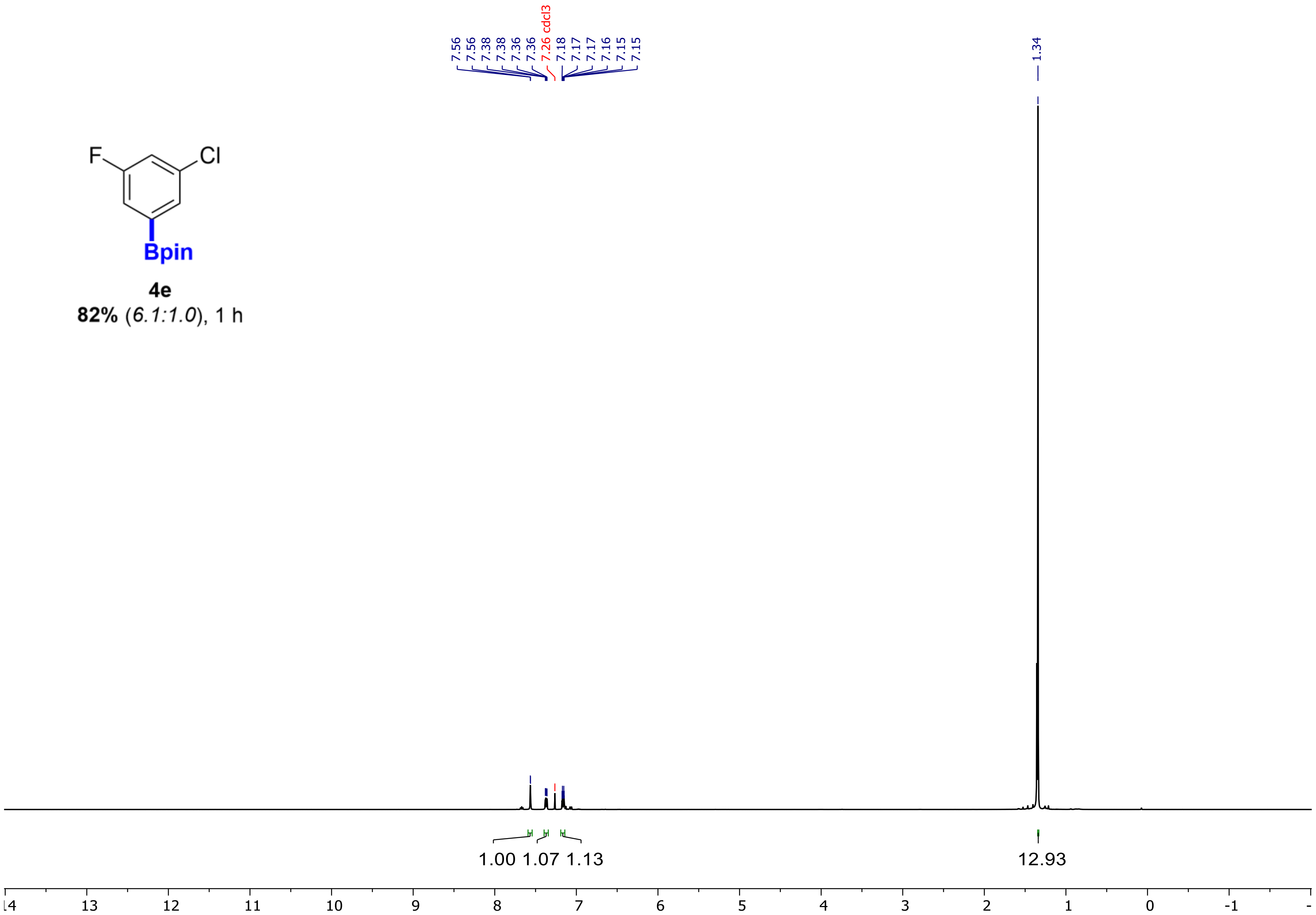

Figure S26. ^{1}H NMR of **4e** (500 MHz, $CDCl_3$)

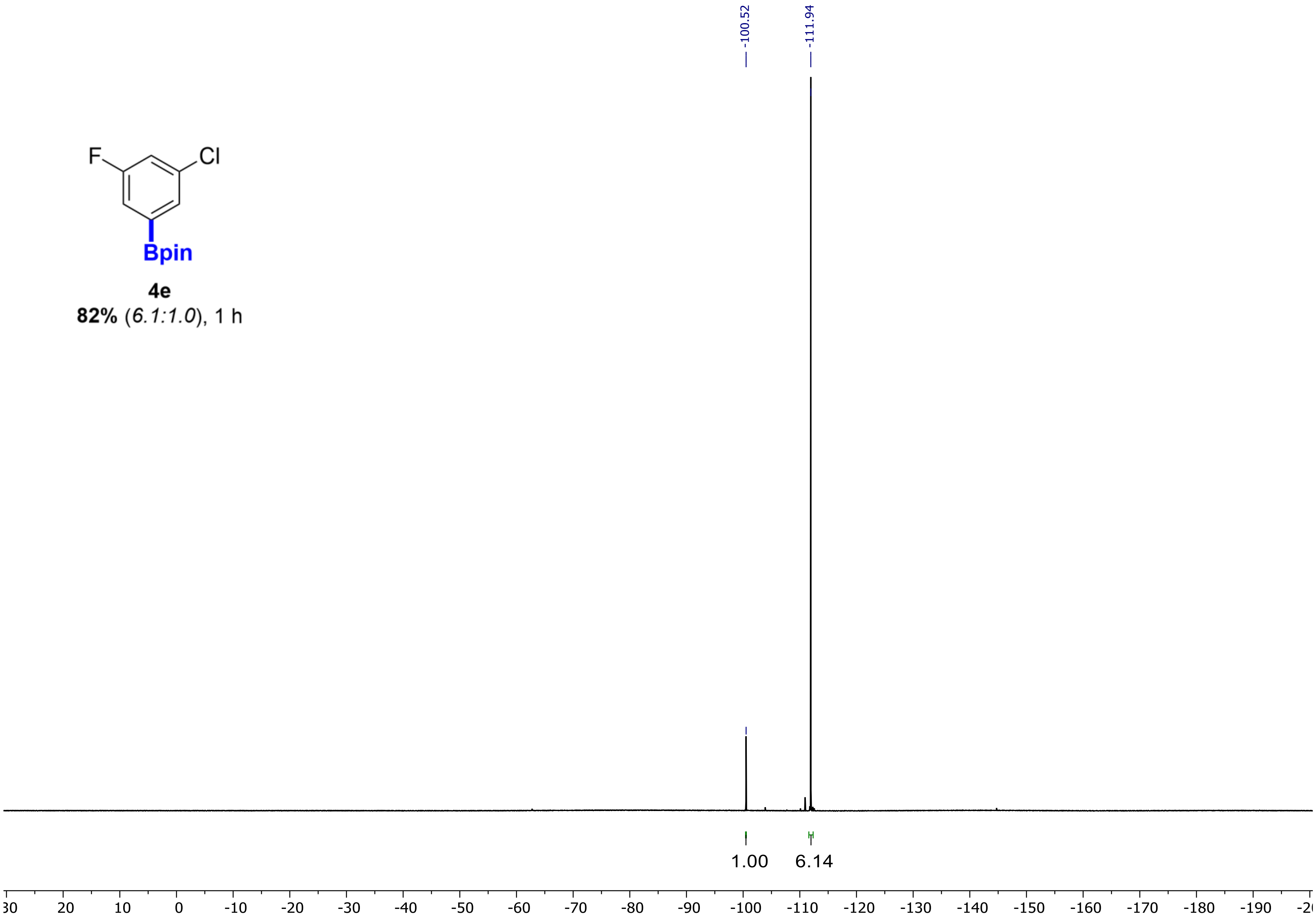

Figure S27. ^{19}F NMR of **4e** (470 MHz, $CDCl_3$)

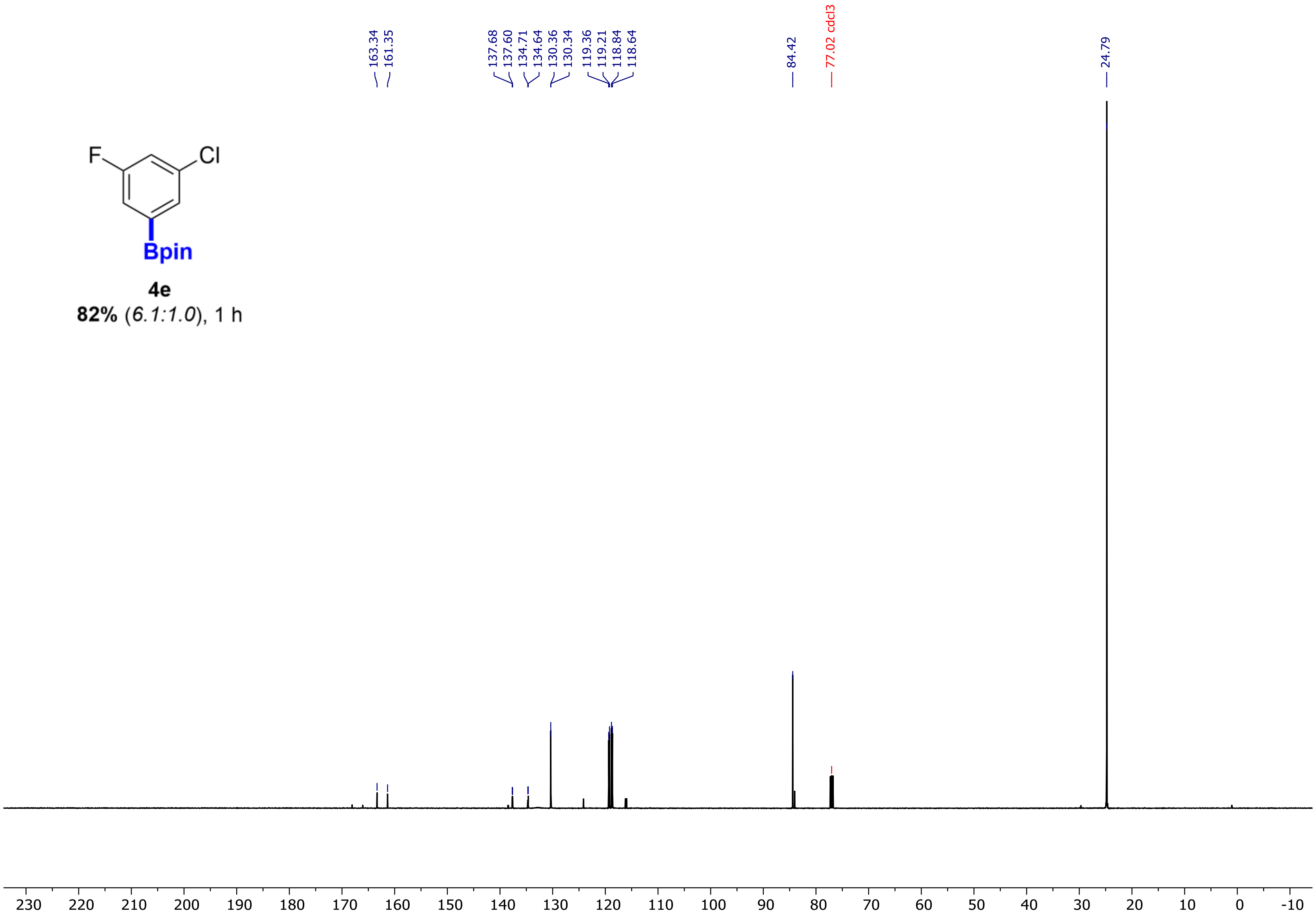

Figure S28. ^{13}C NMR of **4e** (126 MHz, $CDCl_3$)

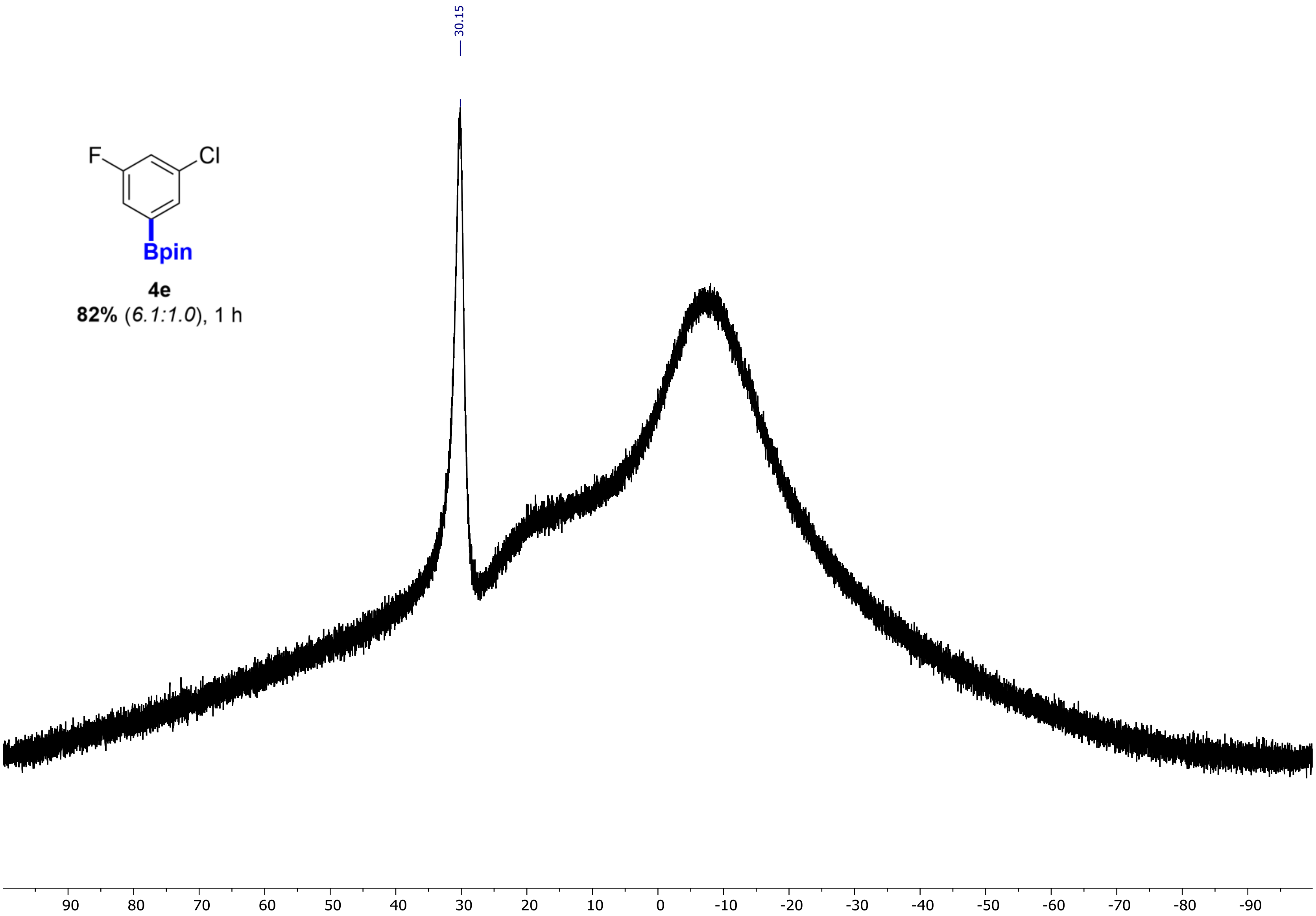

Figure S29. ^{11}B NMR of **4e** (160 MHz, $CDCl_3$)

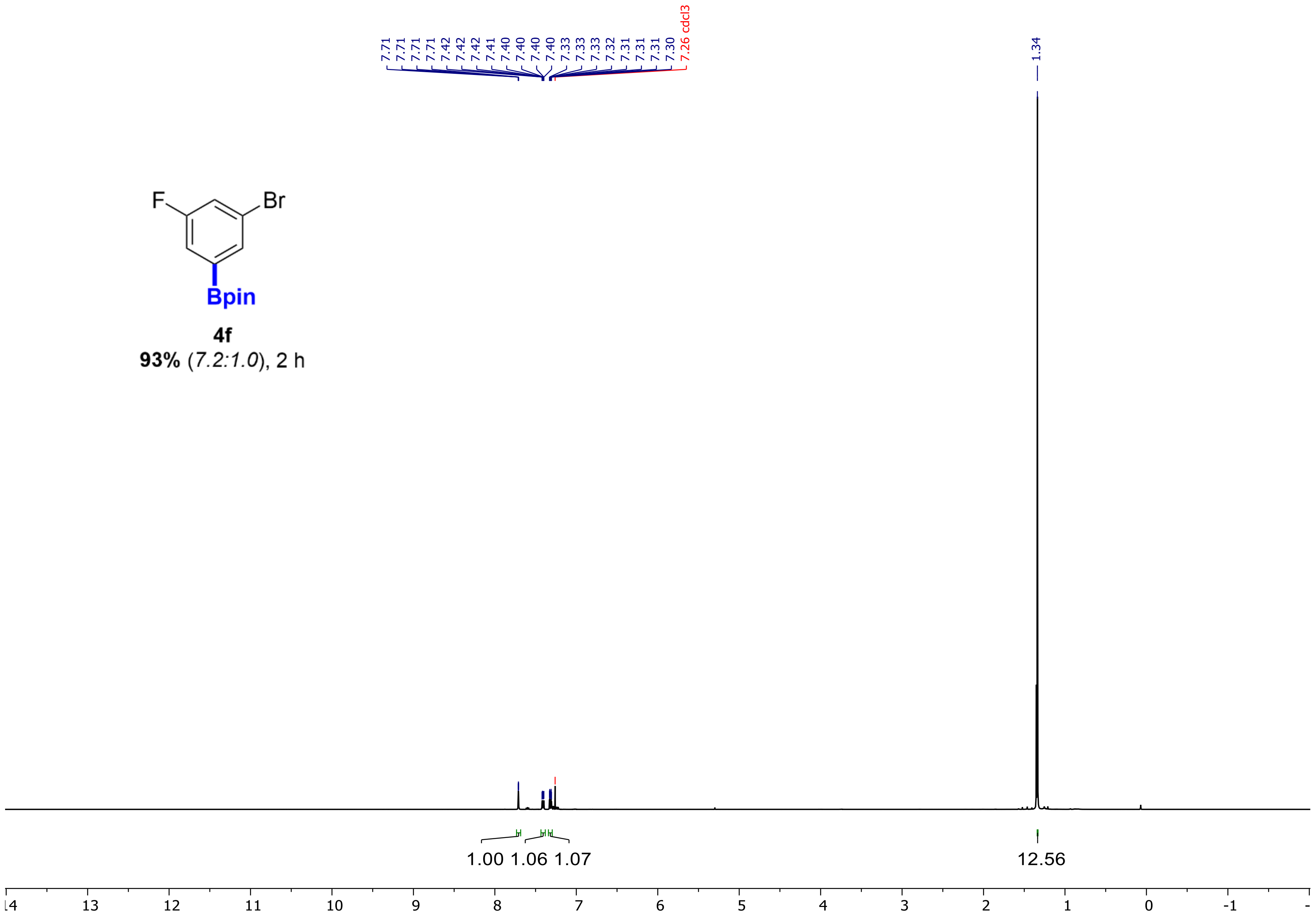

Figure S30. ^{1}H NMR of **4f** (500 MHz, $CDCl_3$)

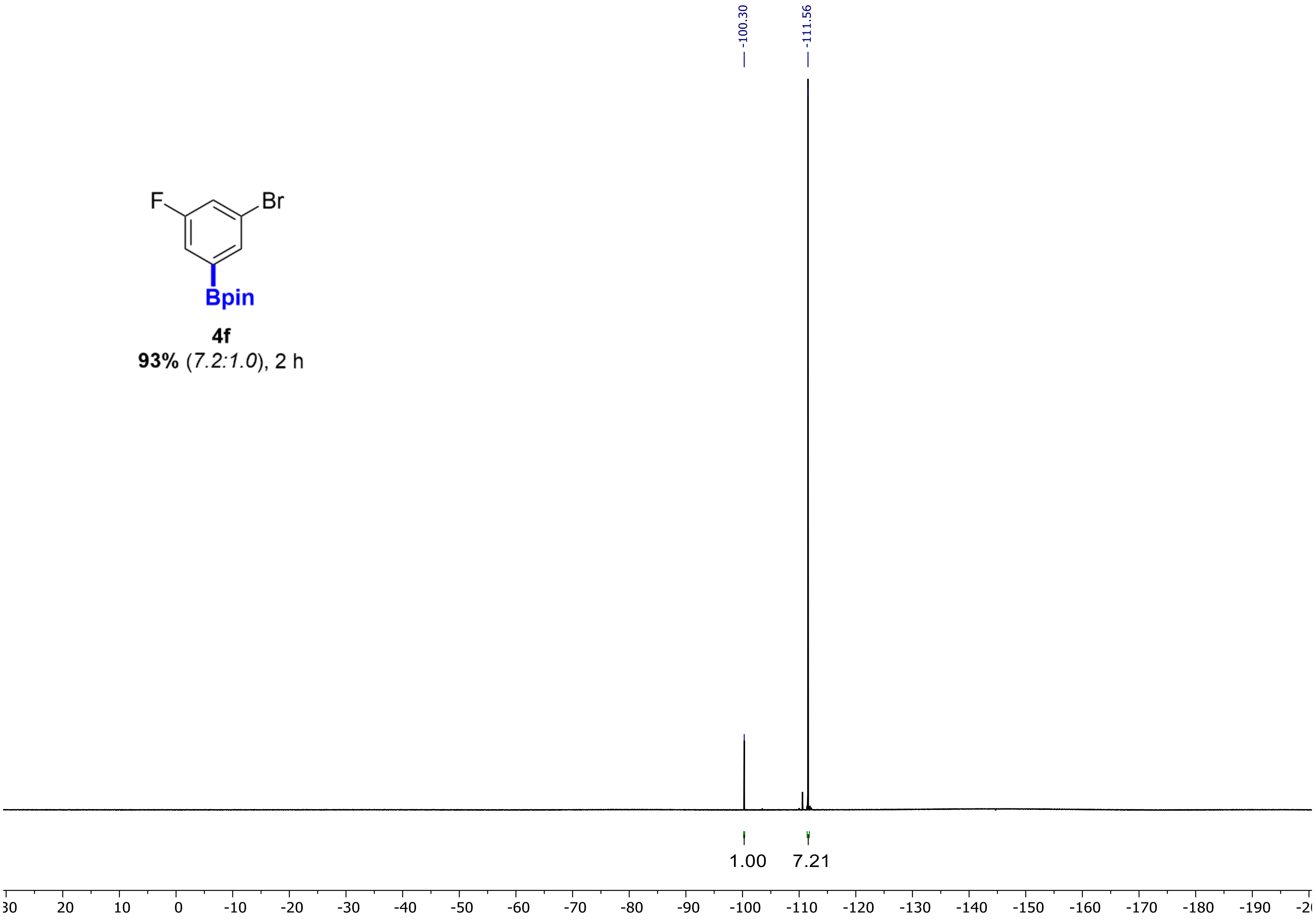

Figure S31. ^{19}F NMR of **4f** (470 MHz, $CDCl_3$)

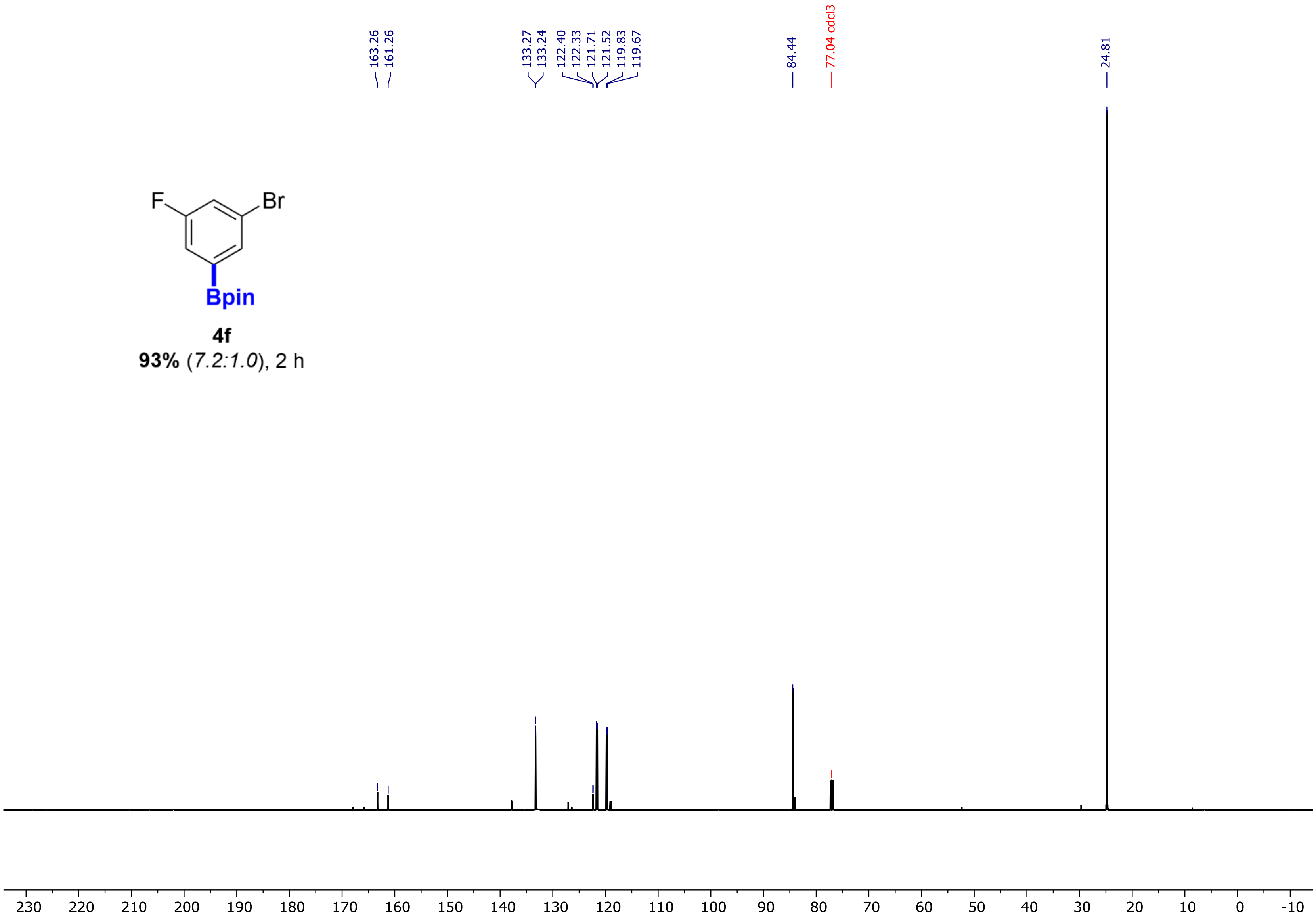

Figure S32. ^{13}C NMR of **4f** (126 MHz, $CDCl_3$)

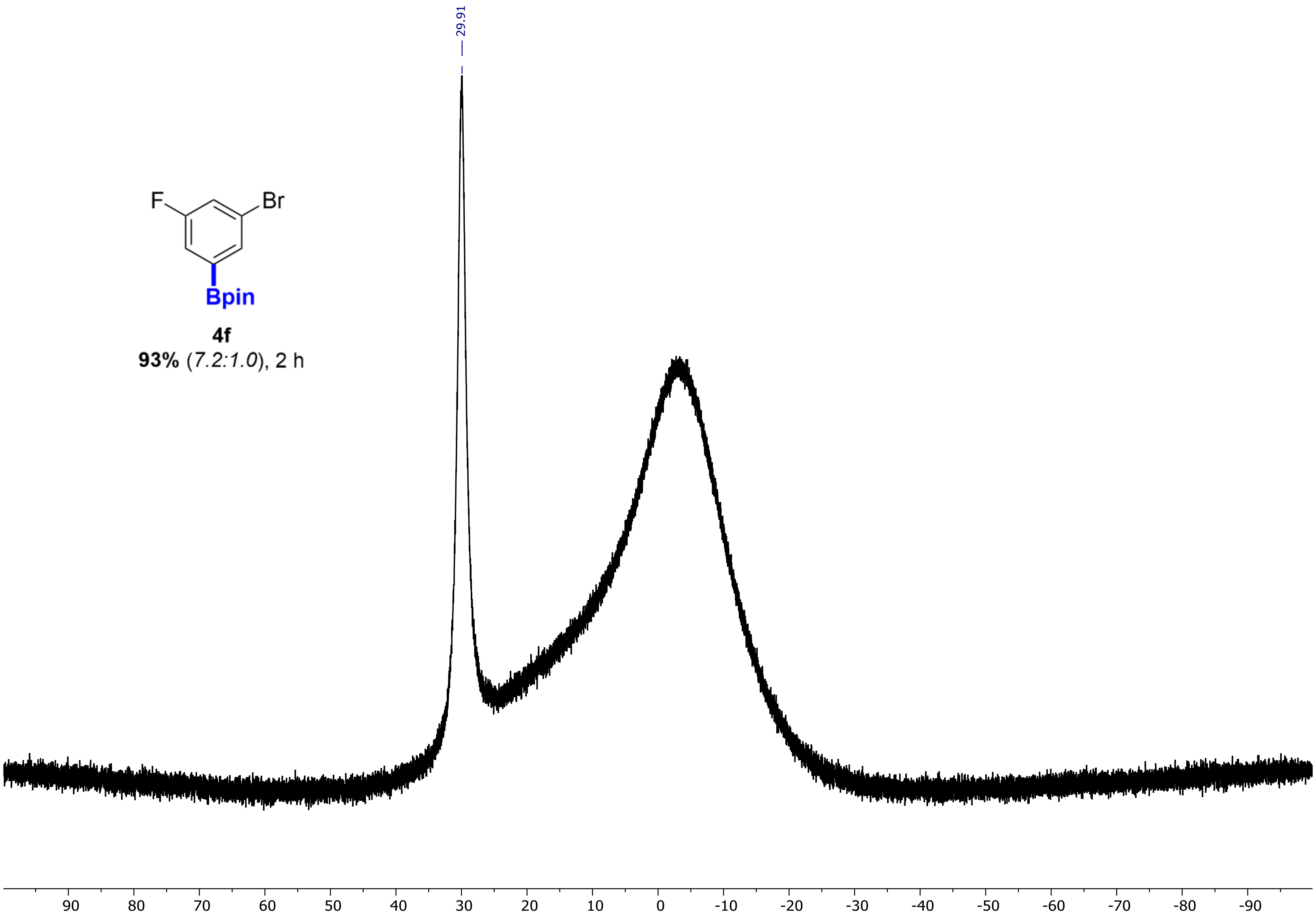

Figure S33. ^{11}B NMR of **4f** (160 MHz, $CDCl_3$)

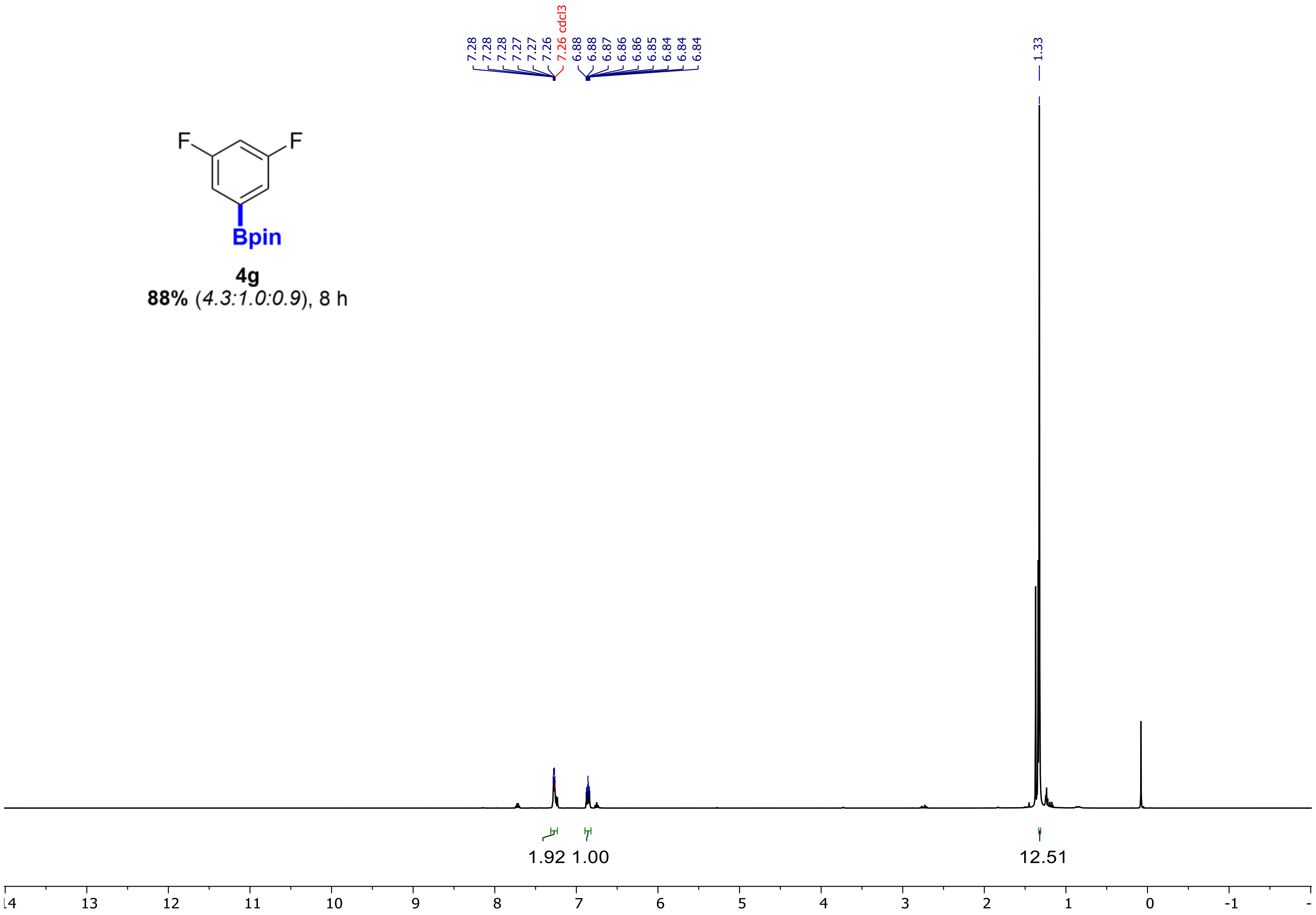

4g
88% (*4.3:1.0:0.9*), 8 h

Figure S34. ^{1}H NMR of **4g** (500 MHz, $CDCl_3$)

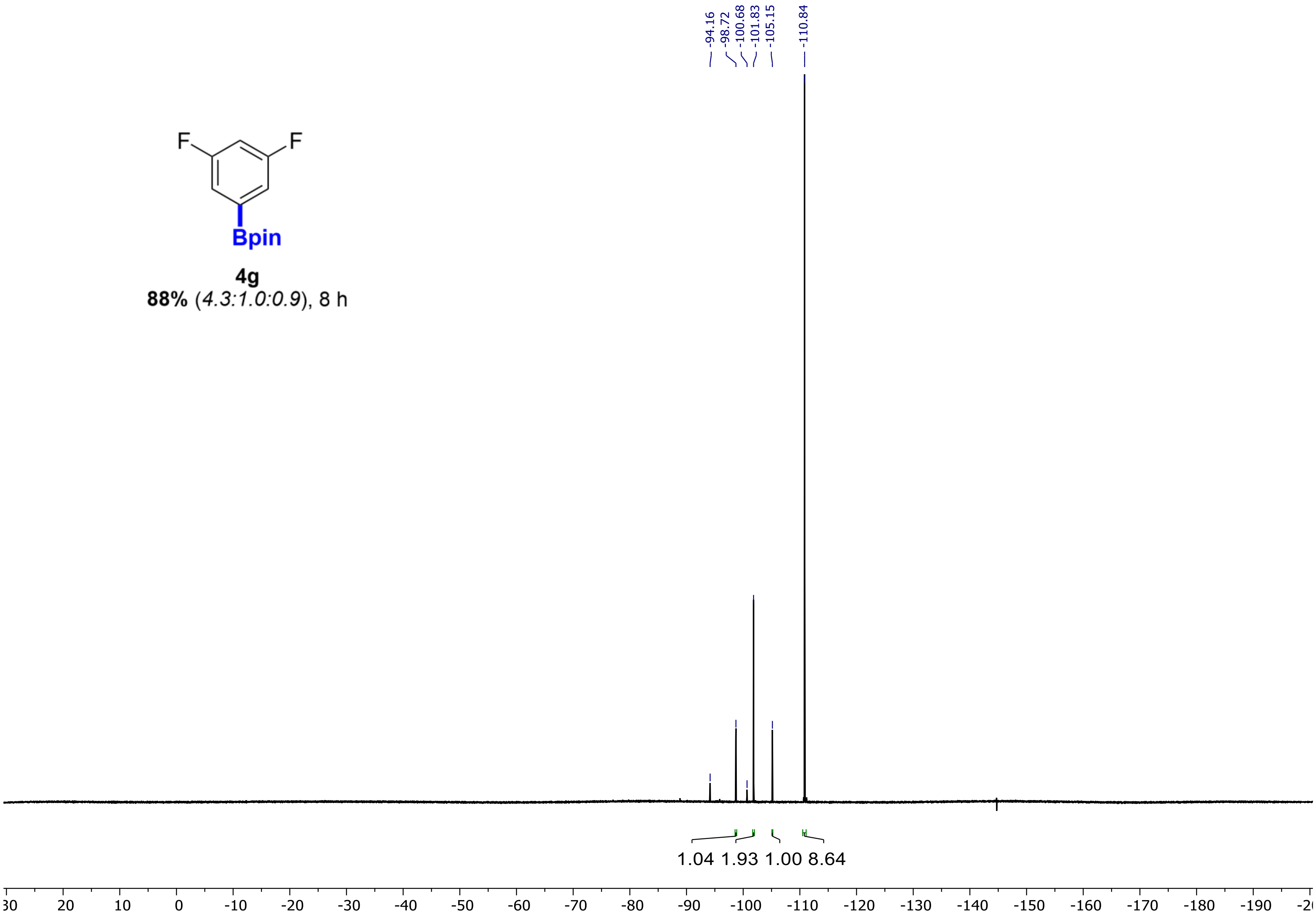

Figure S35. ^{19}F NMR of **4g** (470 MHz, $CDCl_3$)

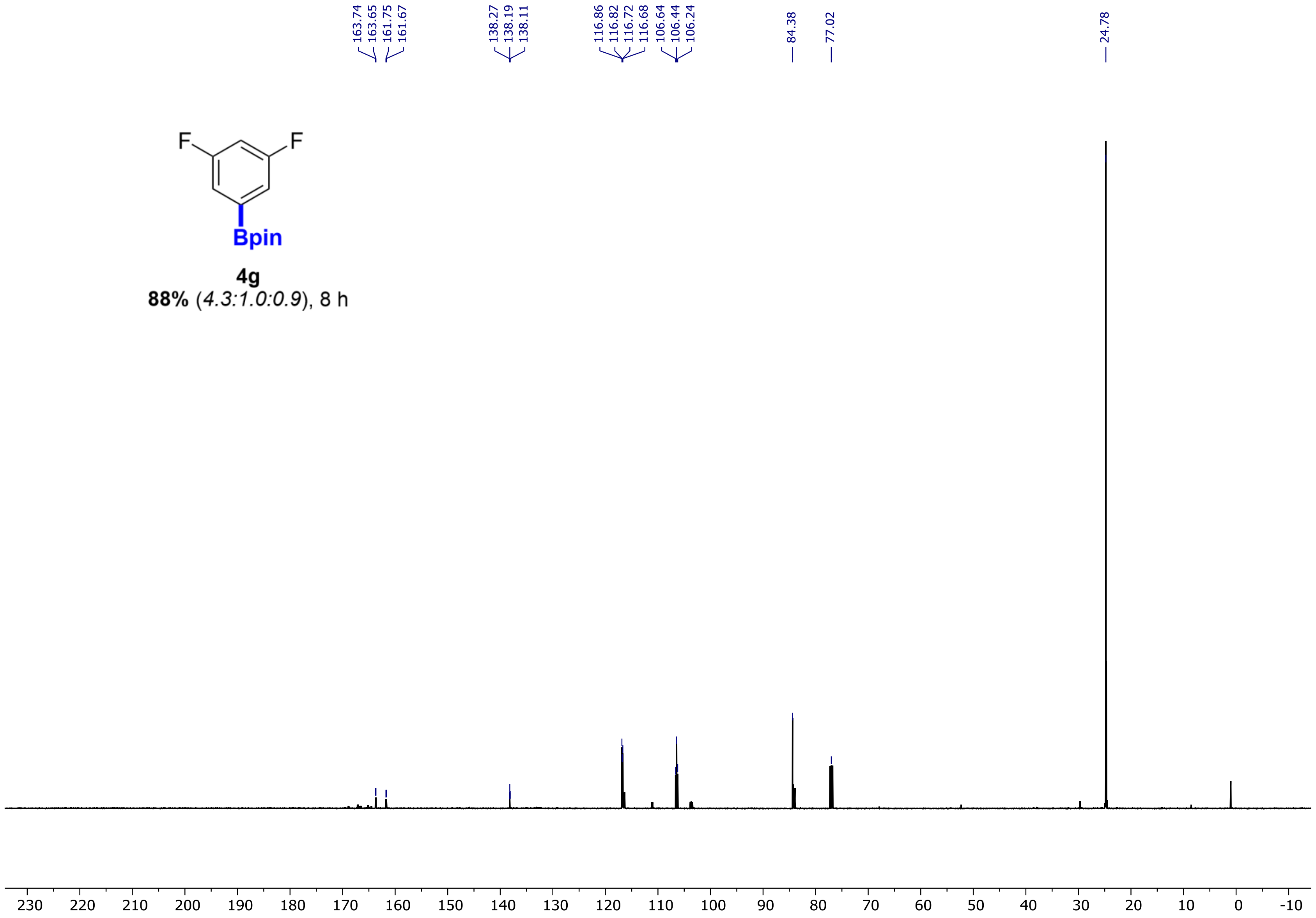

Figure S36. ^{13}C NMR of **4g** (126 MHz, $CDCl_3$)

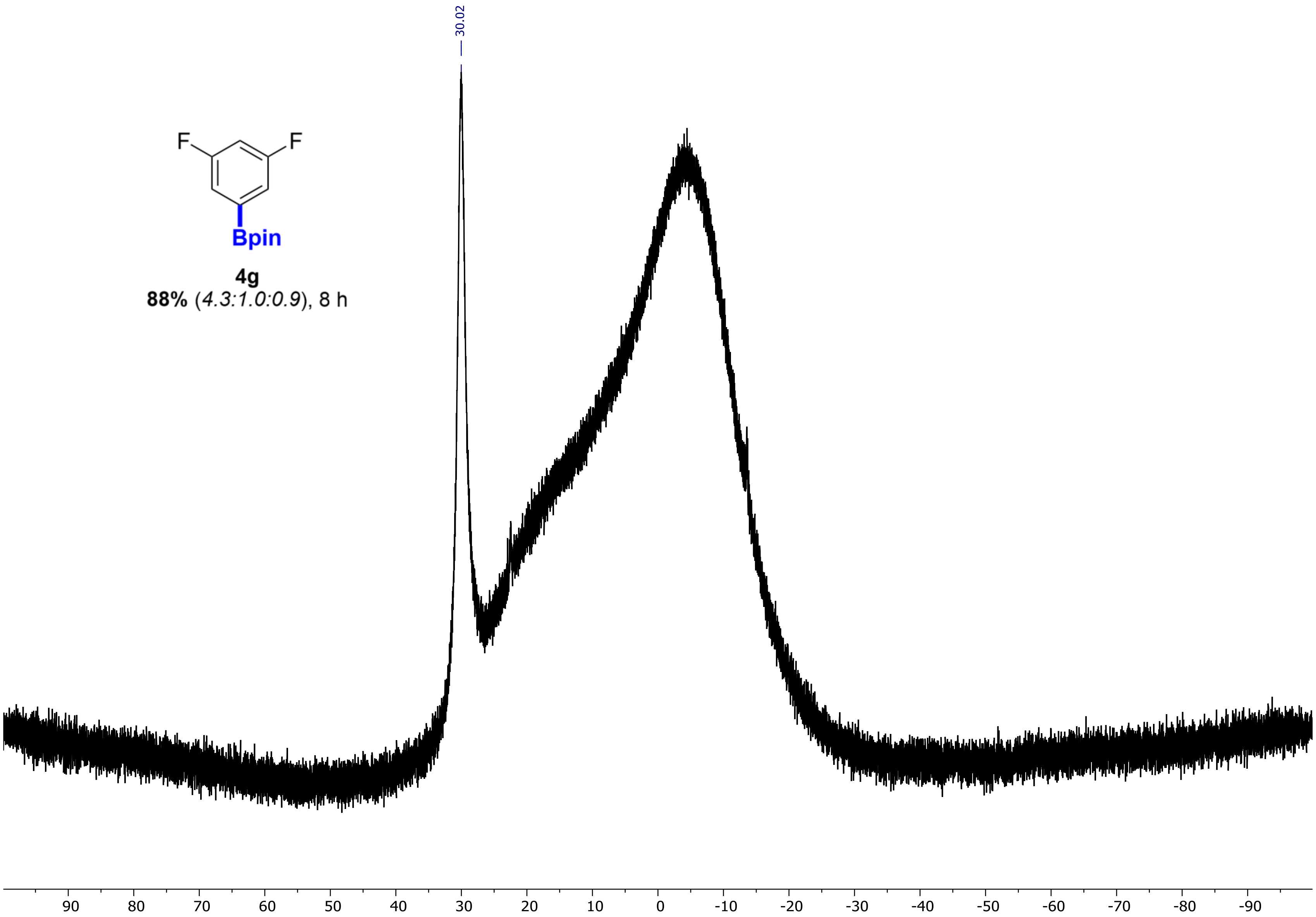

Figure S37. ^{11}B NMR of **4g** (160 MHz, $CDCl_3$)

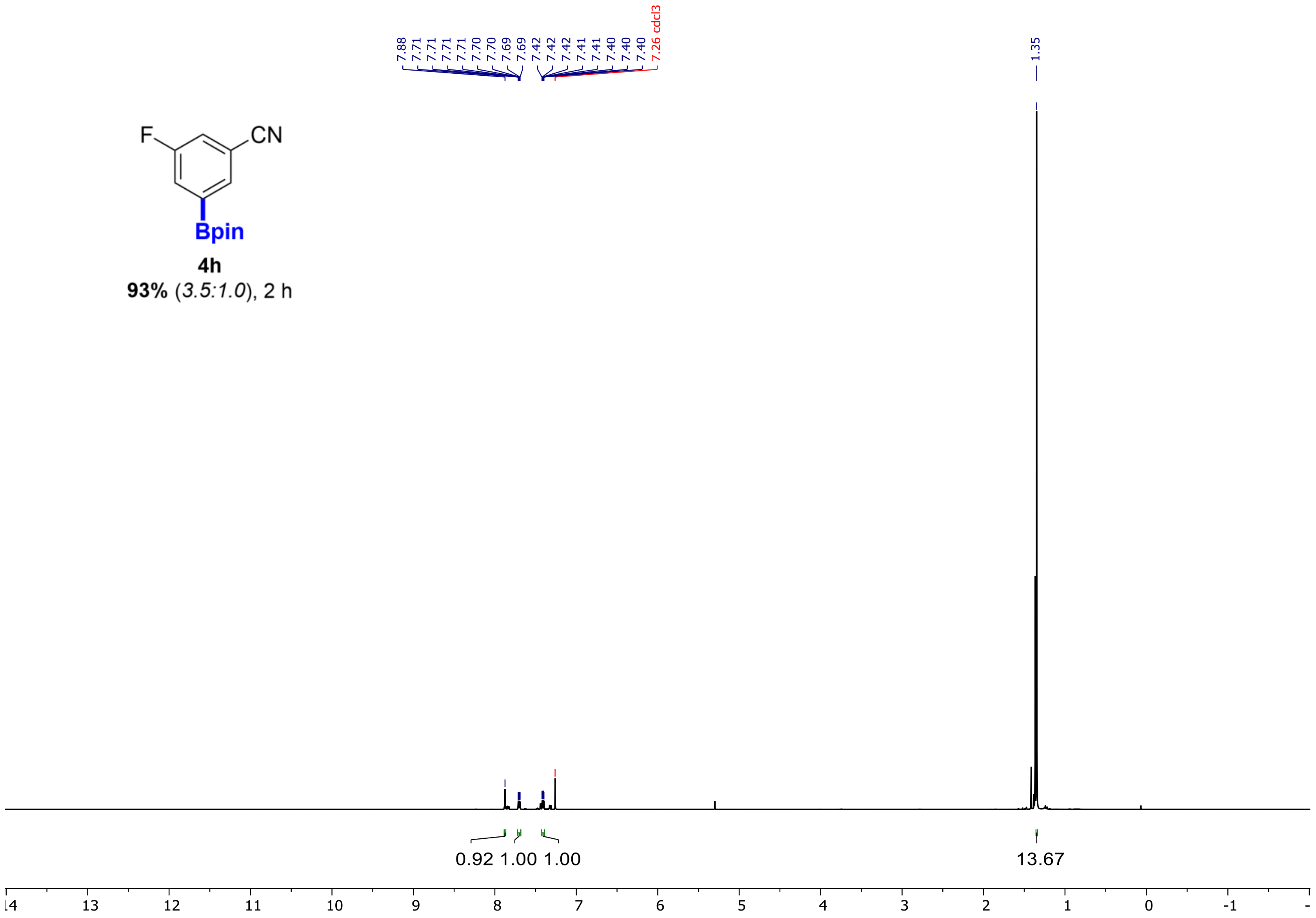

Figure S38. ^{1}H NMR of **4h** (500 MHz, $CDCl_3$)

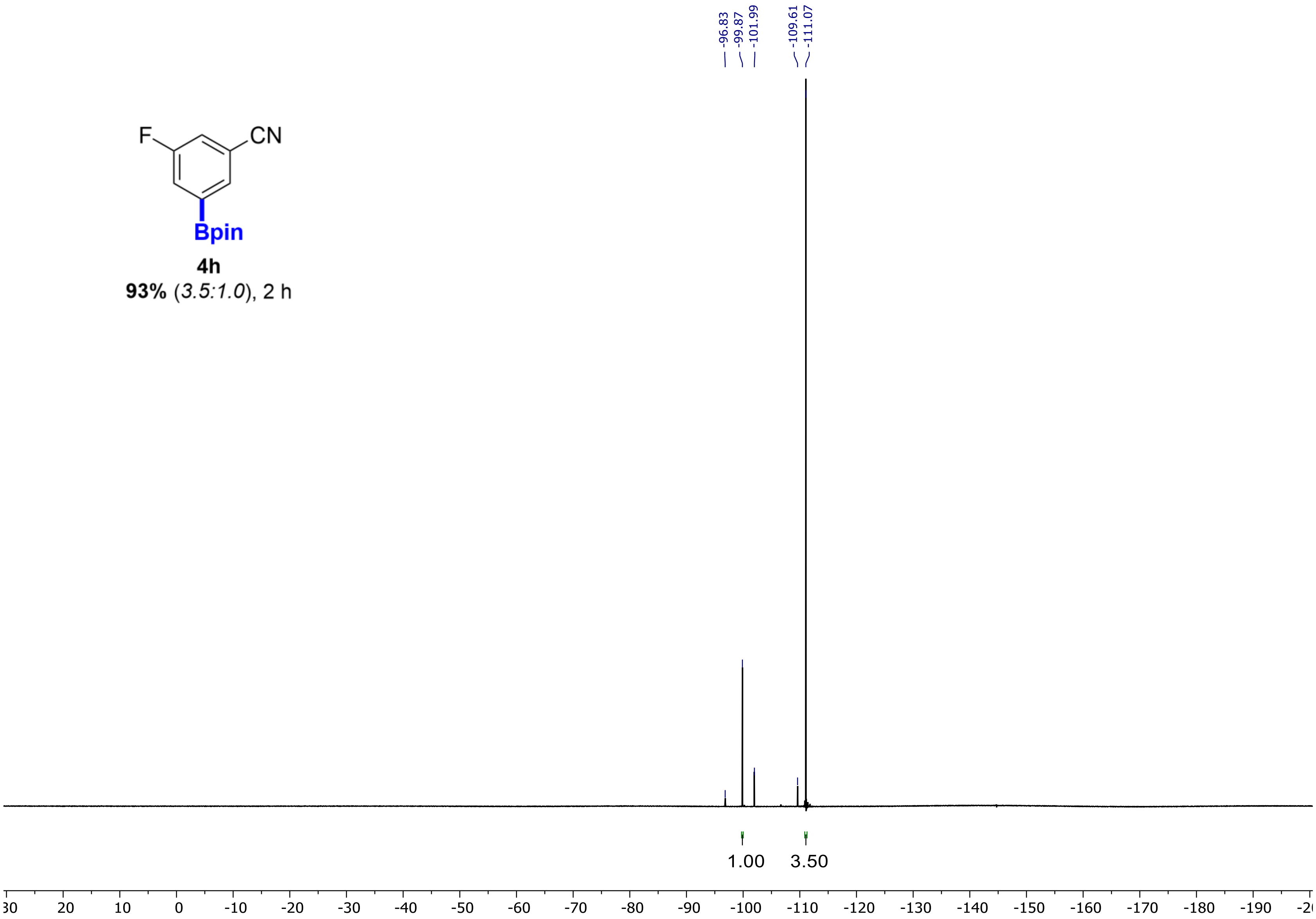

Figure S39. ^{19}F NMR of **4h** (470 MHz, $CDCl_3$)

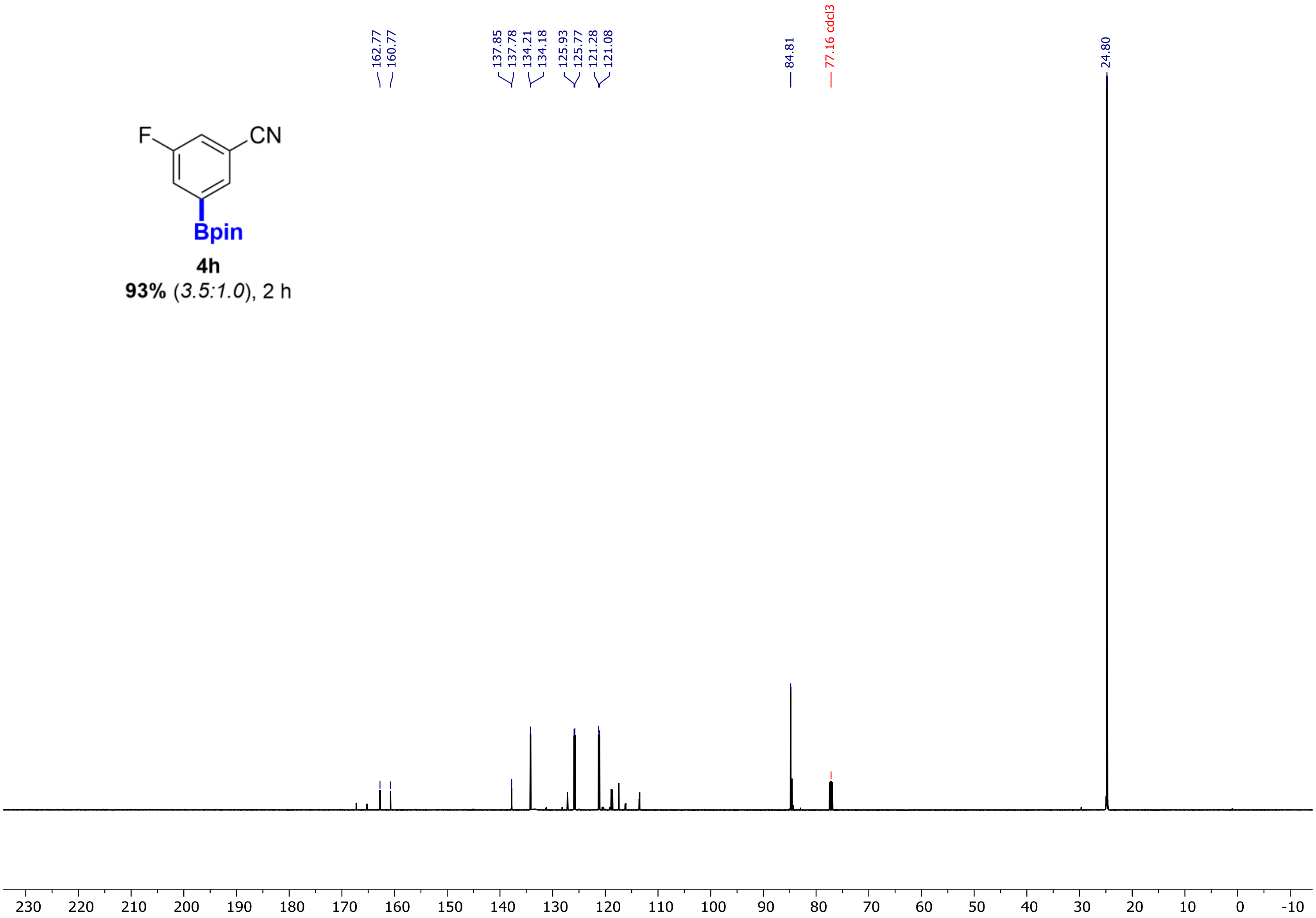

4h
93% (*3.5:1.0*), 2 h

Figure S39. ^{13}C NMR of **4h** (126 MHz, $CDCl_3$)

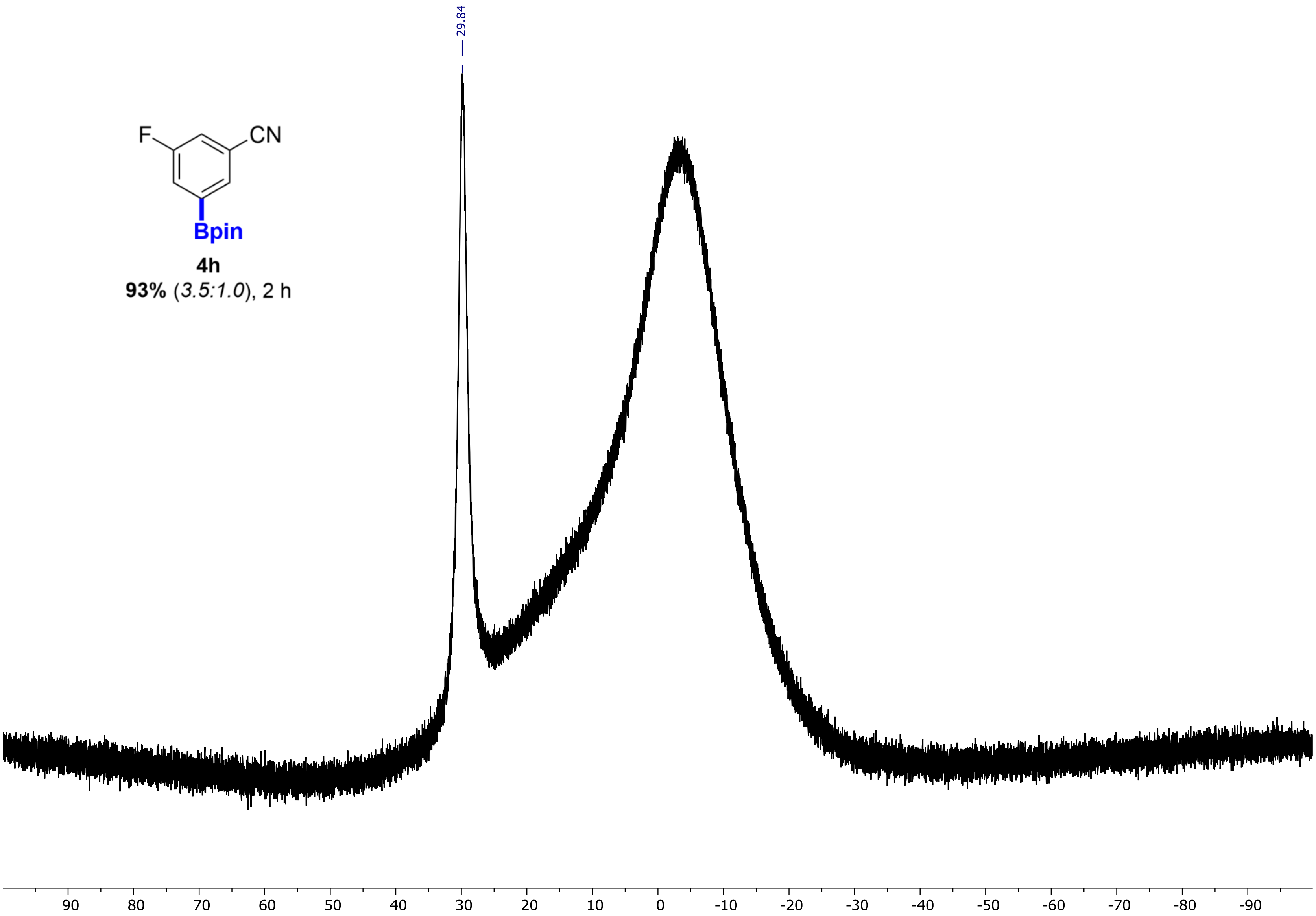

Figure S40. ^{11}B NMR of **4h** (160 MHz, $CDCl_3$)

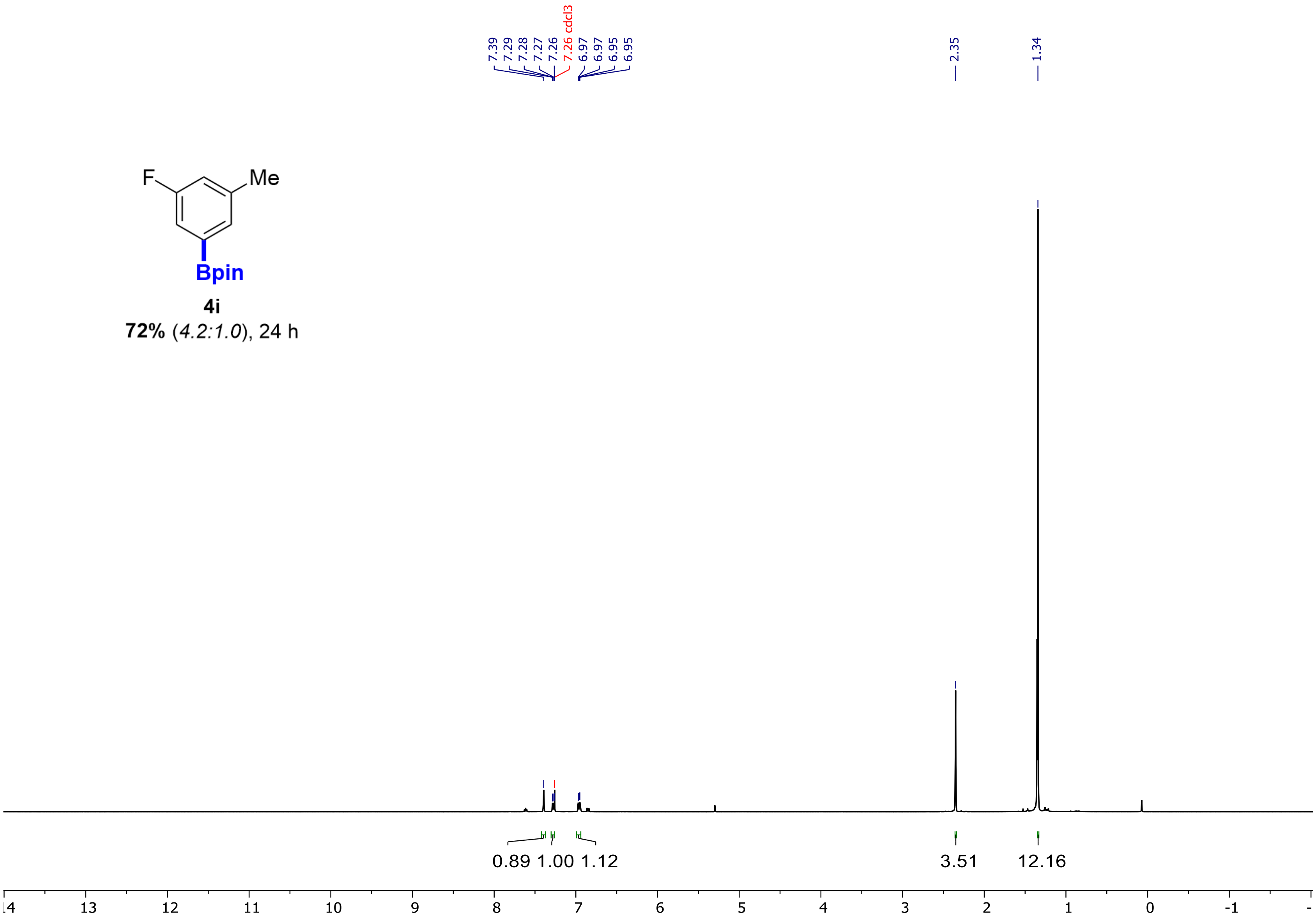

Figure S41. ^{1}H NMR of **4i** (500 MHz, $CDCl_3$)

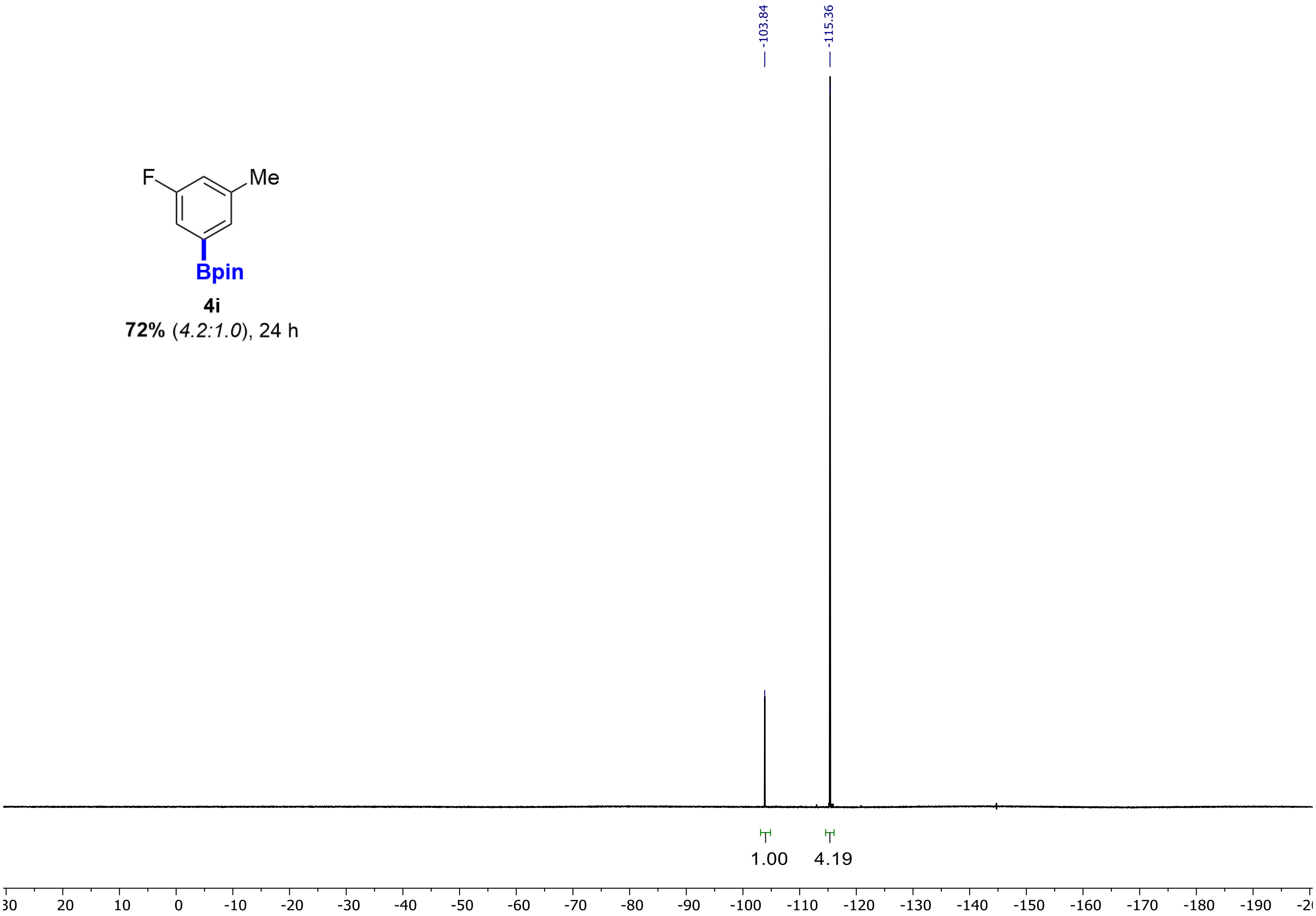

Figure S42. ^{19}F NMR of **4i** (470 MHz, $CDCl_3$)

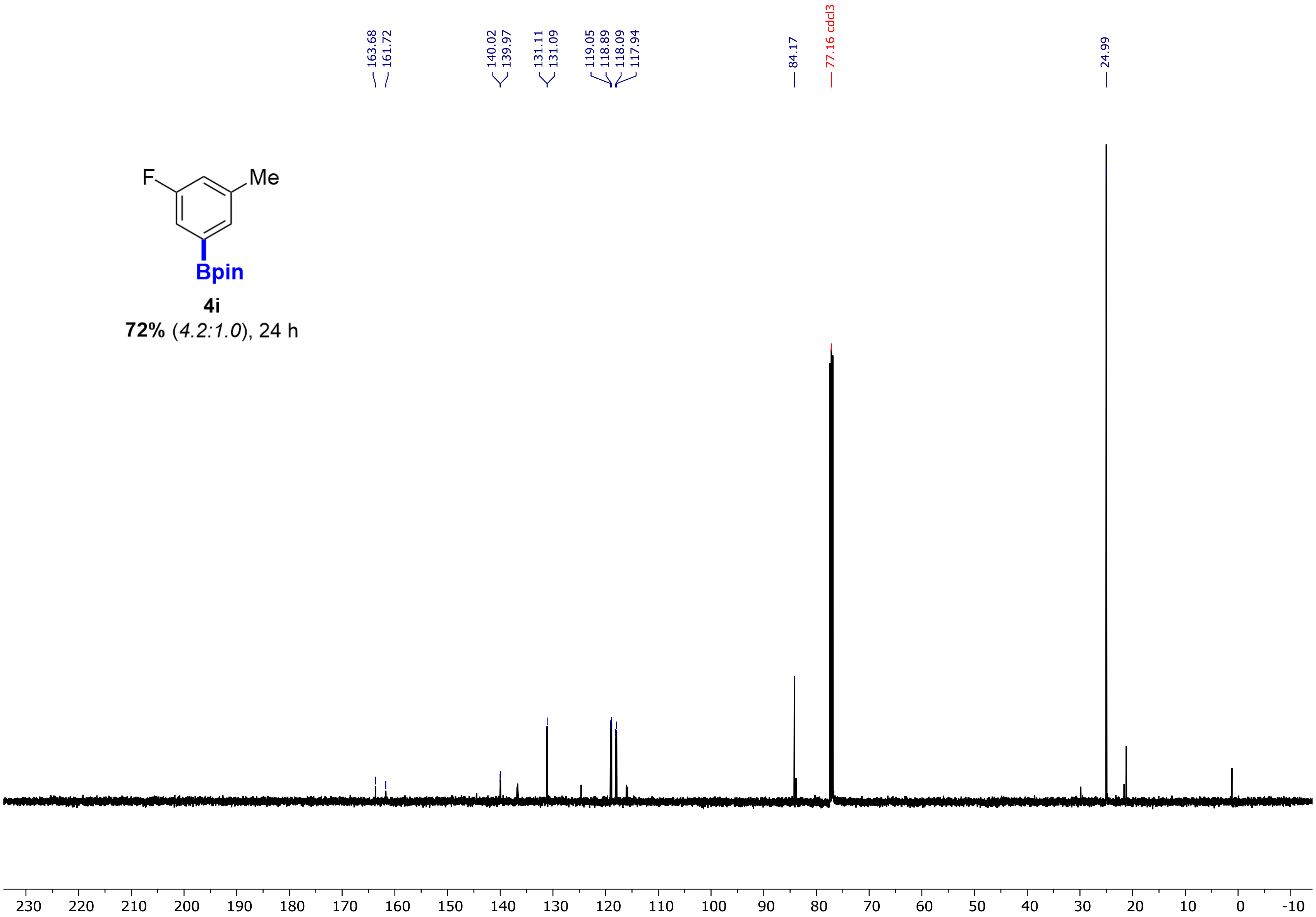

Figure S43. ^{13}C NMR of **4i** (126 MHz, $CDCl_3$)

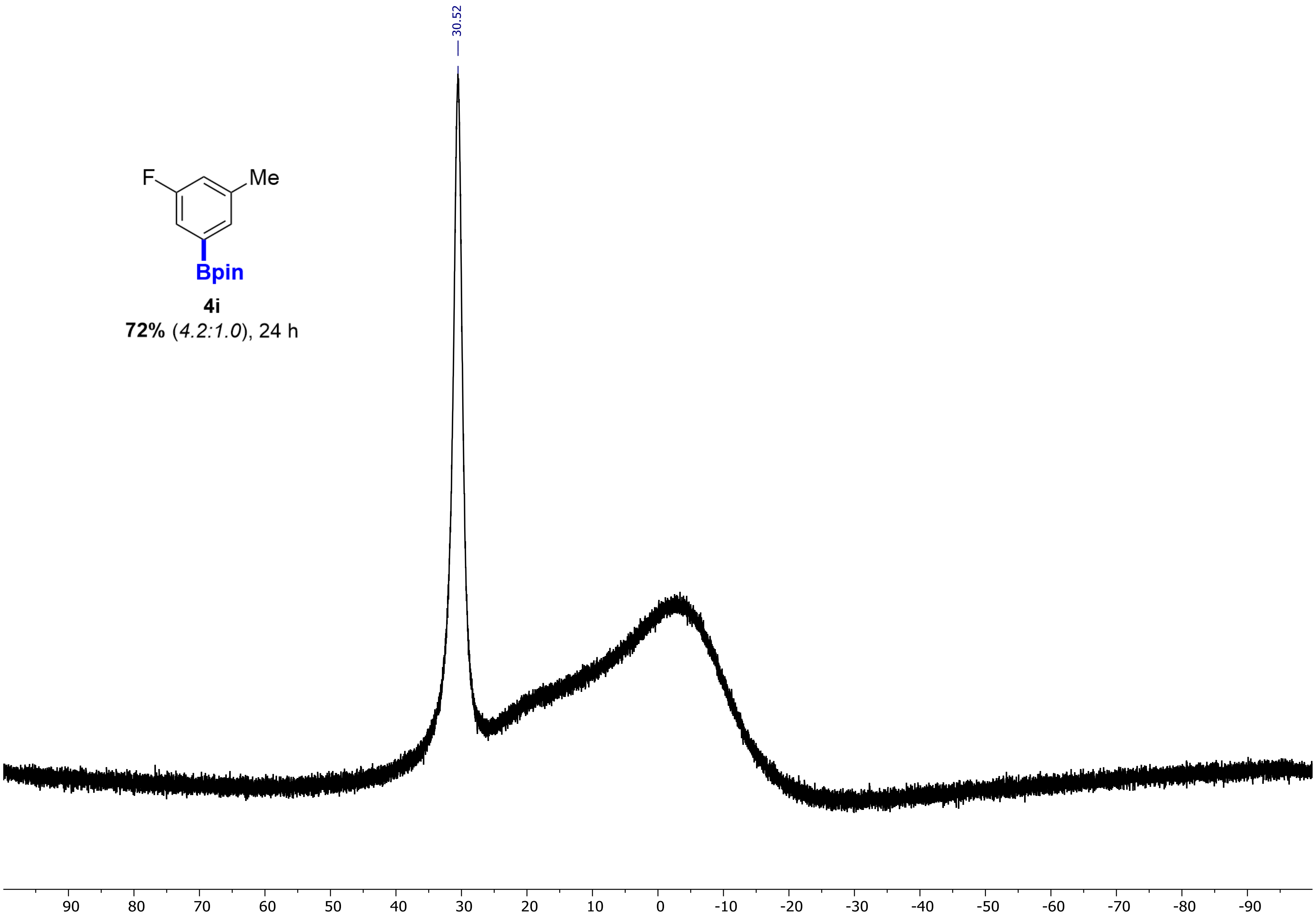

Figure S44. ^{11}B NMR of **4i** (160 MHz, $CDCl_3$)

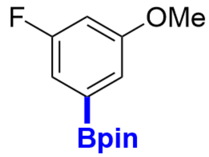

4j
80% (*5.8:1.0*), 24 h

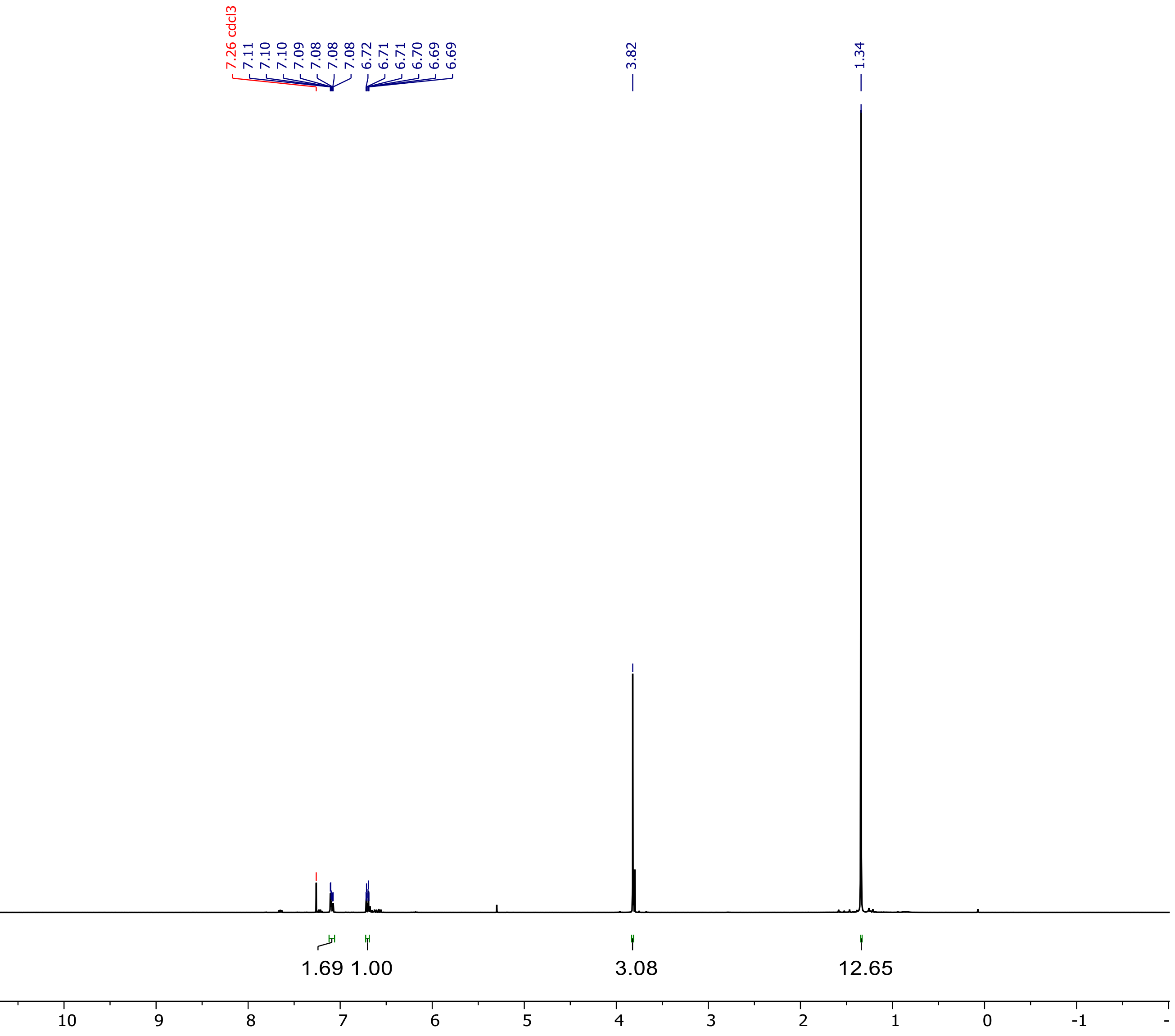

Figure S45. ^{1}H NMR of **4j** (500 MHz, $CDCl_3$)

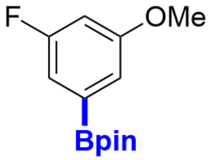

4j

80% (*5.8:1.0*), 24 h

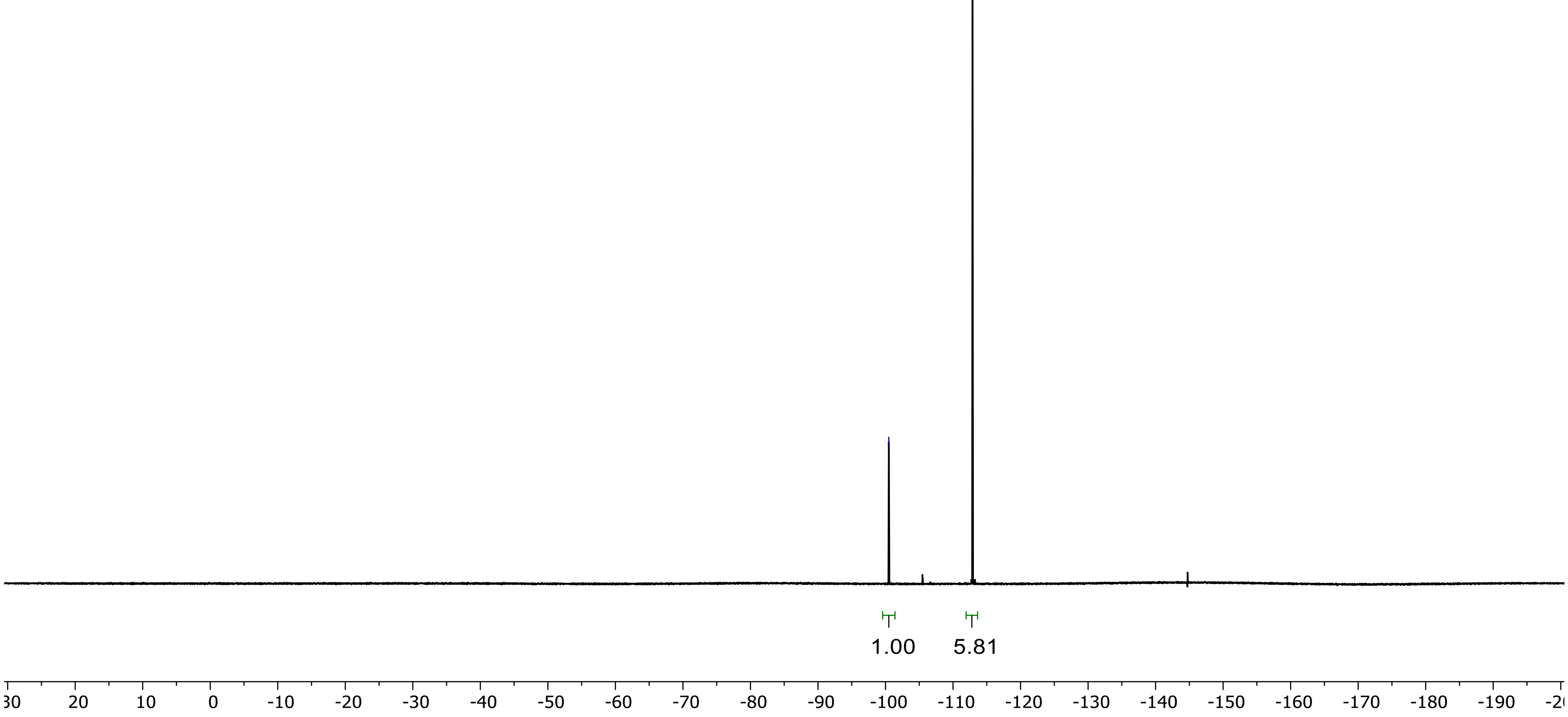

Figure S46. ^{19}F NMR of **4j** (470 MHz, $CDCl_3$)

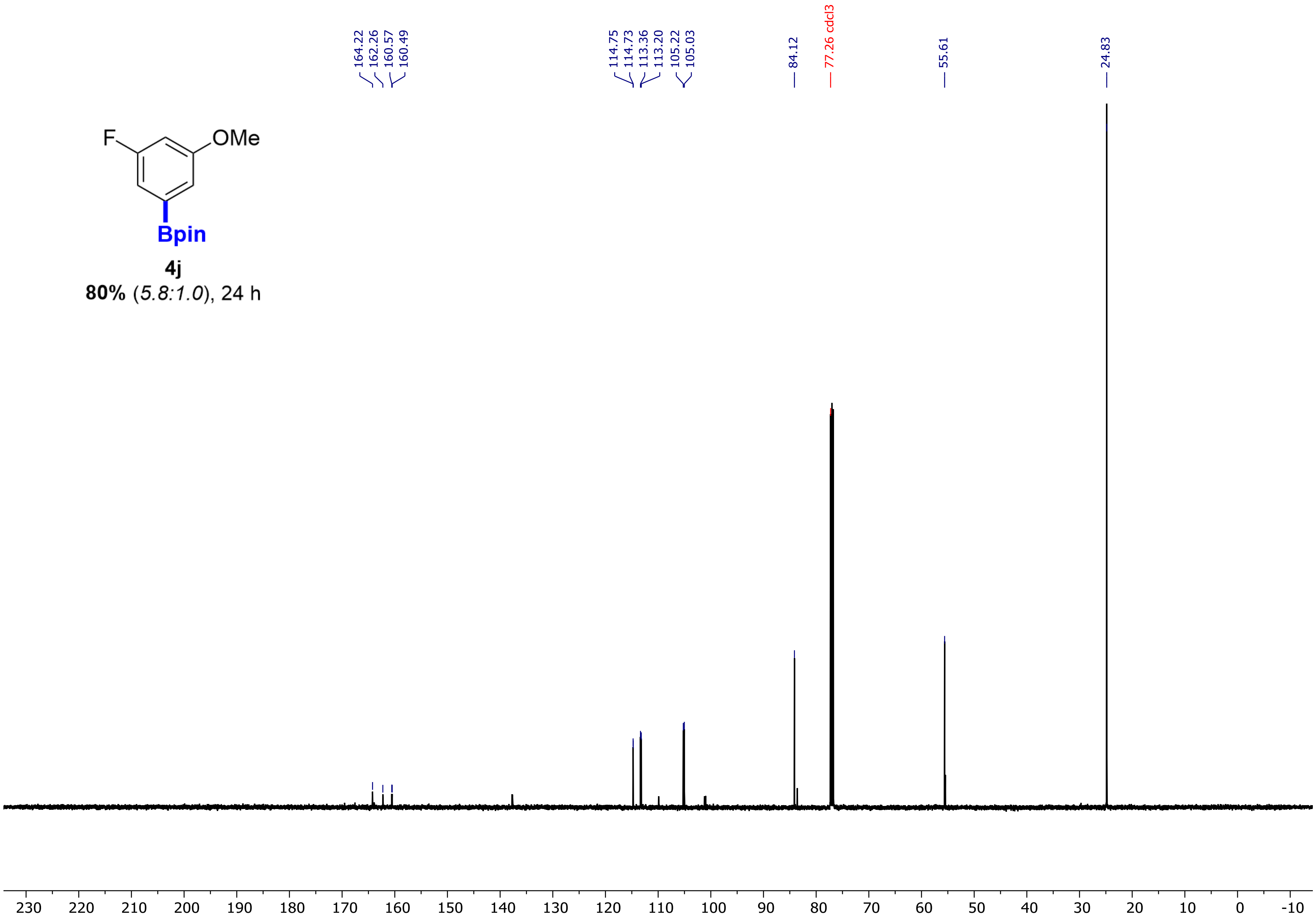

Figure S47. ^{13}C NMR of **4j** (126 MHz, $CDCl_3$)

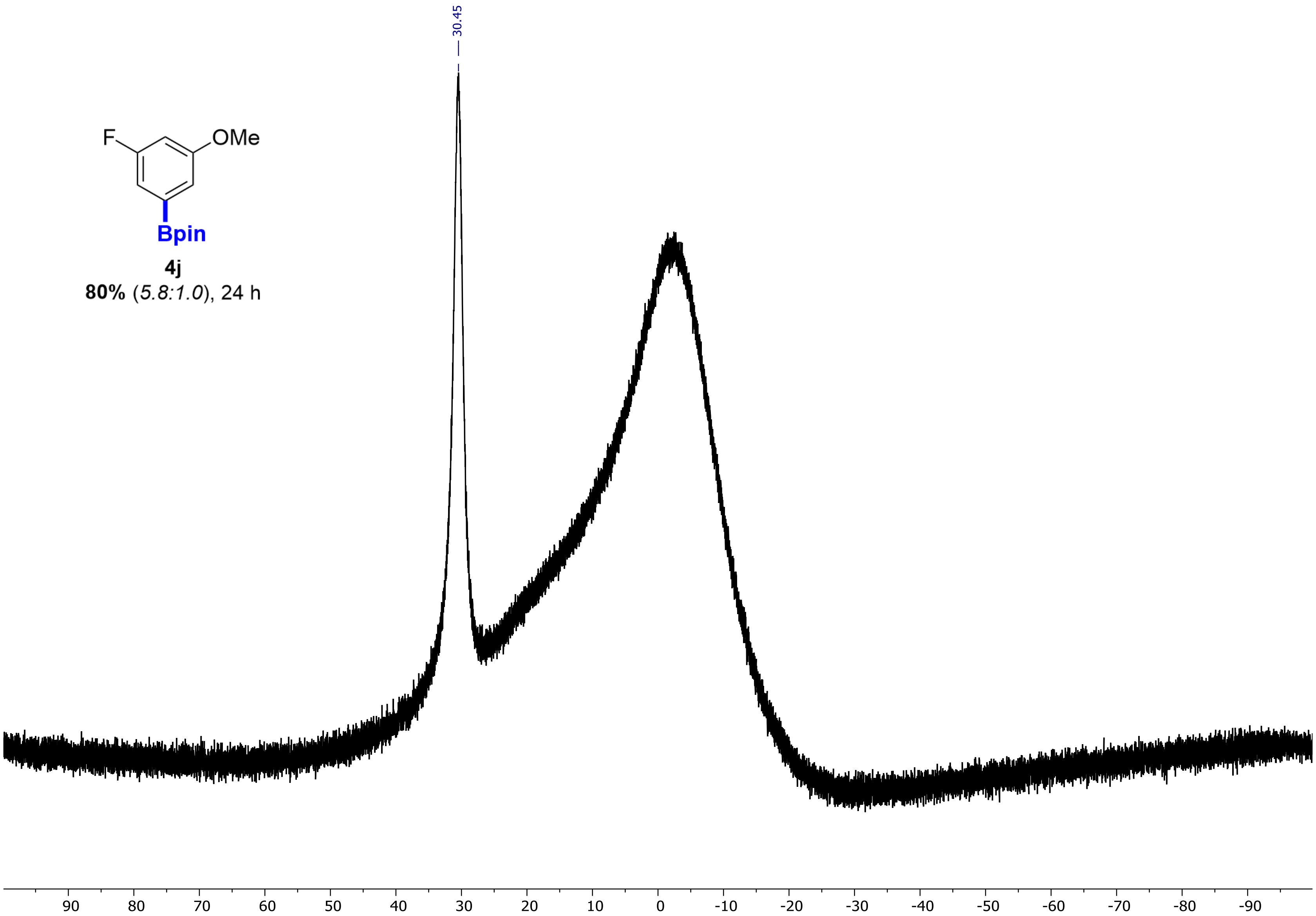

Figure S48. ^{11}B NMR of **4j** (160 MHz, $CDCl_3$)

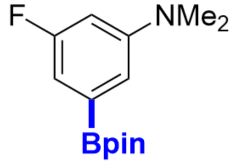

4k

80% (*3.1:1.0*), 24 h

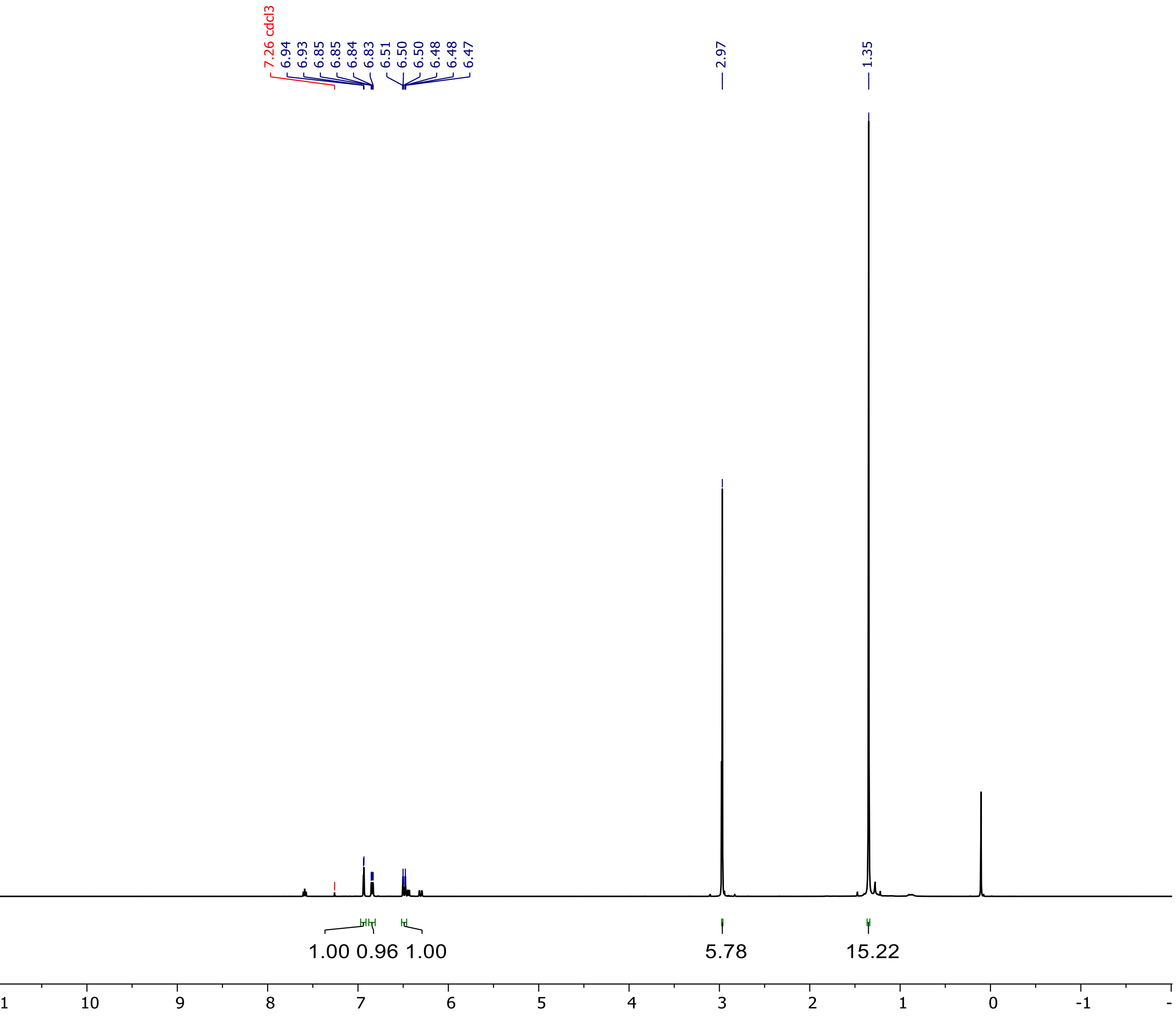

Figure S49. ^{1}H NMR of **4k** (500 MHz, $CDCl_3$)

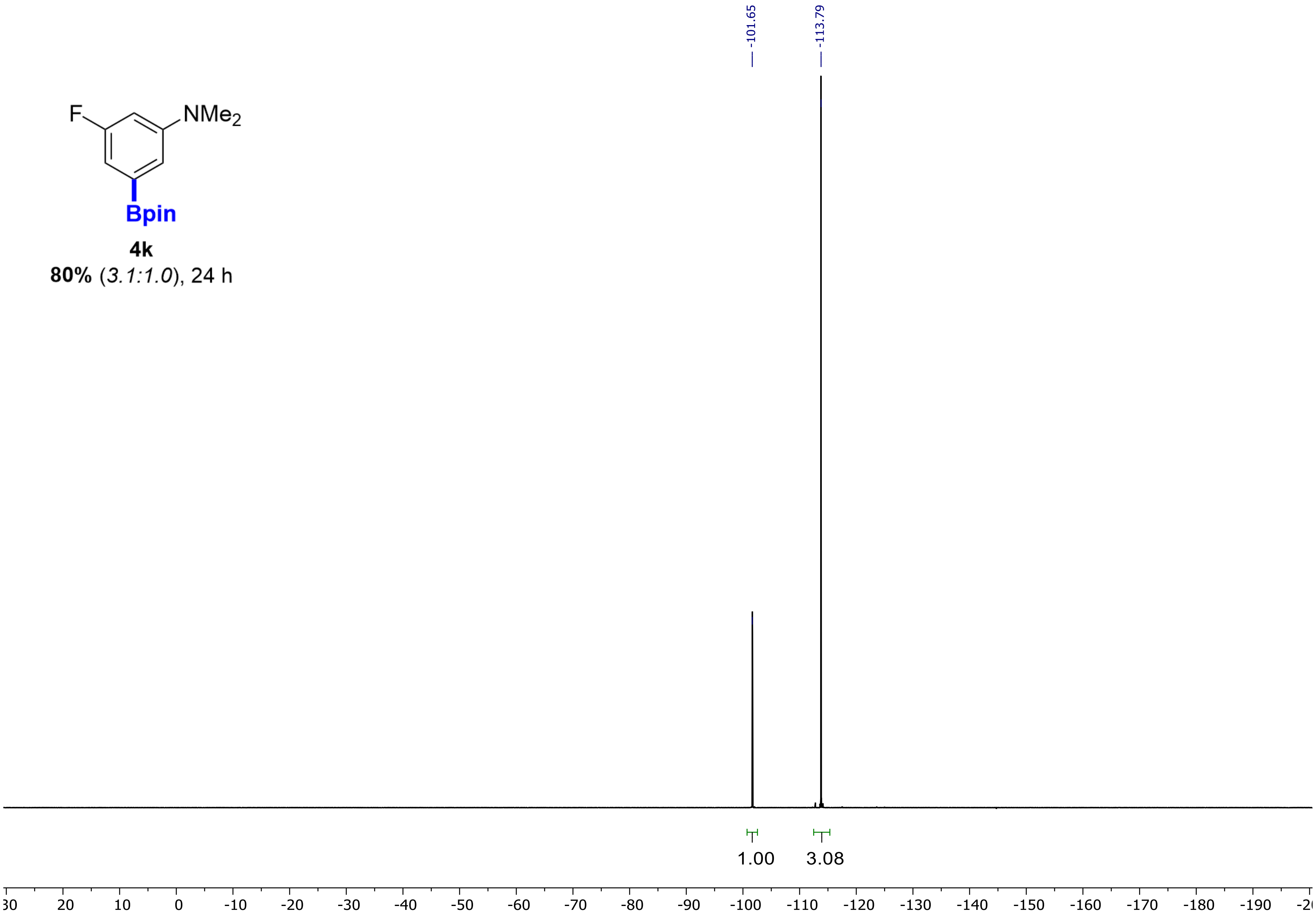

Figure S50. ^{19}F NMR of **4k** (470 MHz, $CDCl_3$)

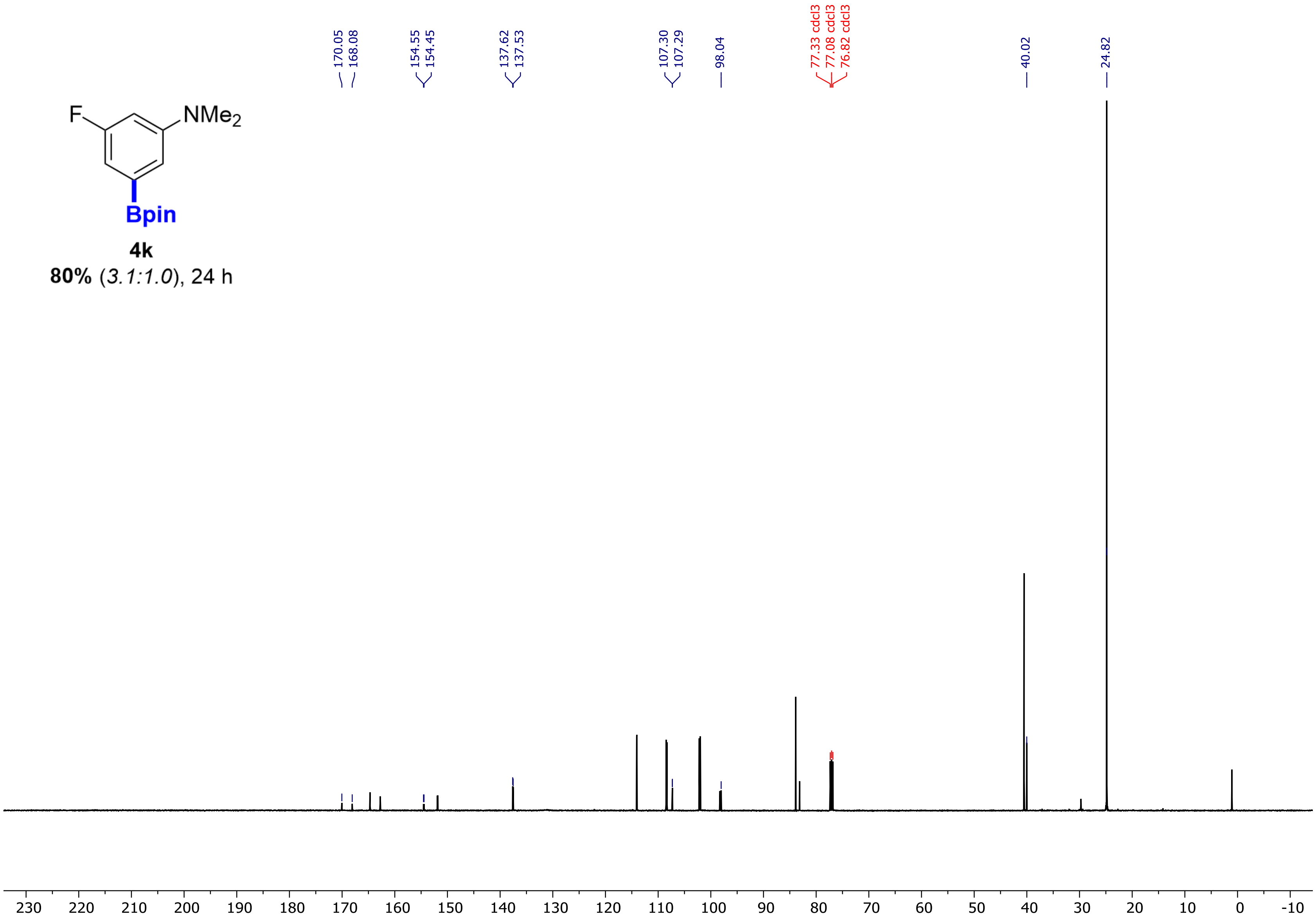

Figure S51. ^{13}C NMR of **4k** (126 MHz, $CDCl_3$)

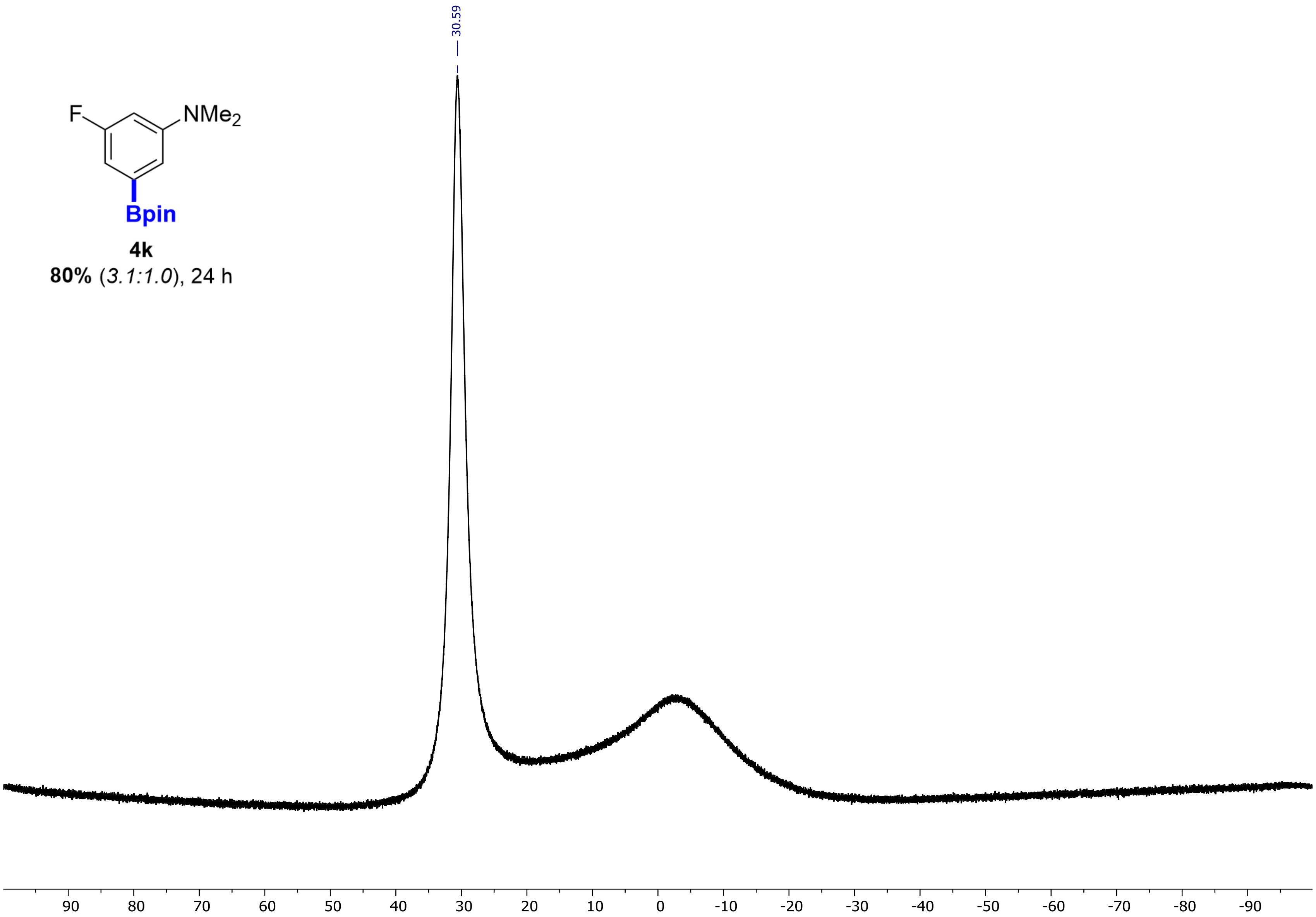

4k

80% (*3.1:1.0*), 24 h

Figure S52. ^{11}B NMR of **4k** (160 MHz, $CDCl_3$)

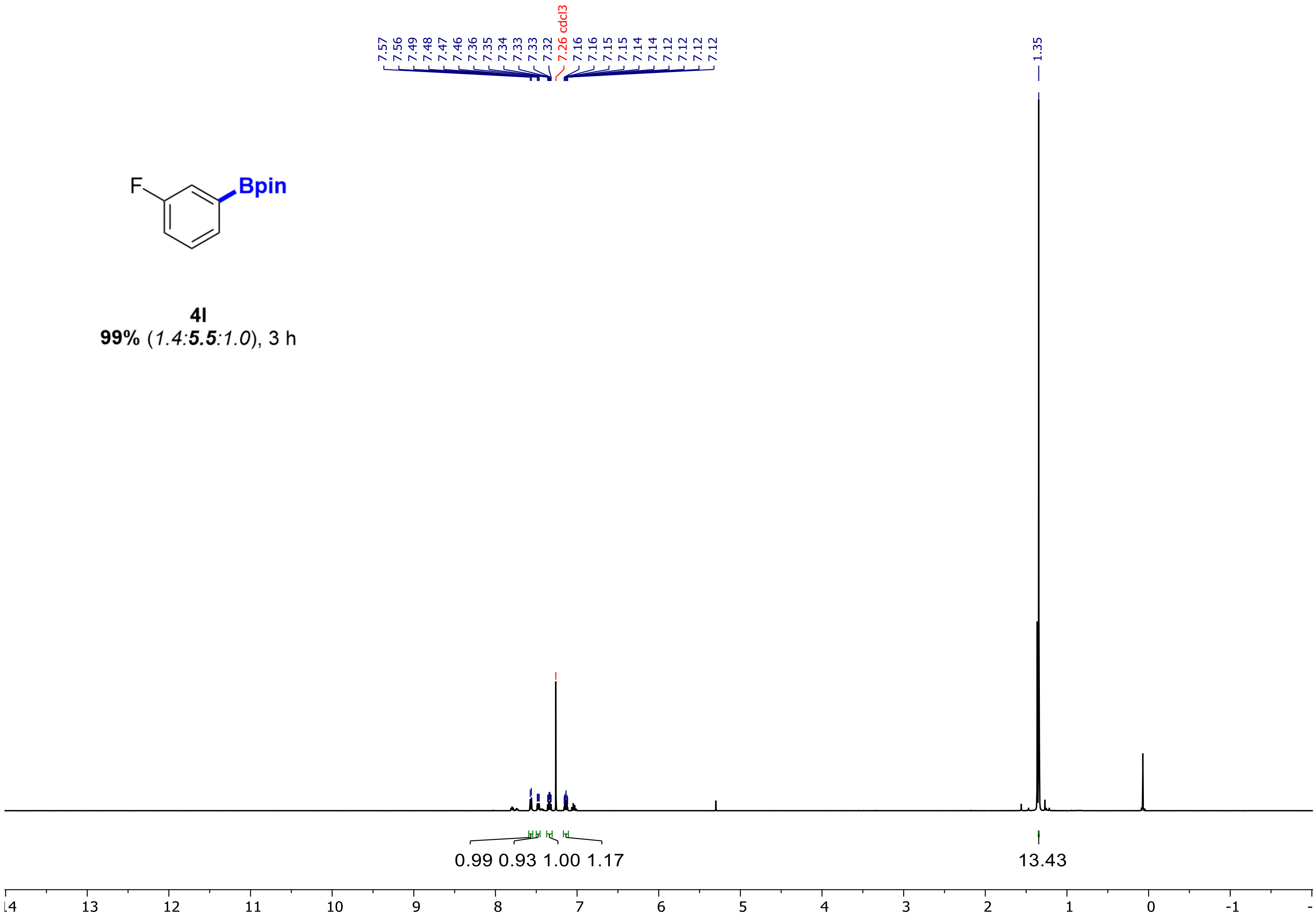

Figure S53. ^{1}H NMR of **4l** (500 MHz, $CDCl_3$)

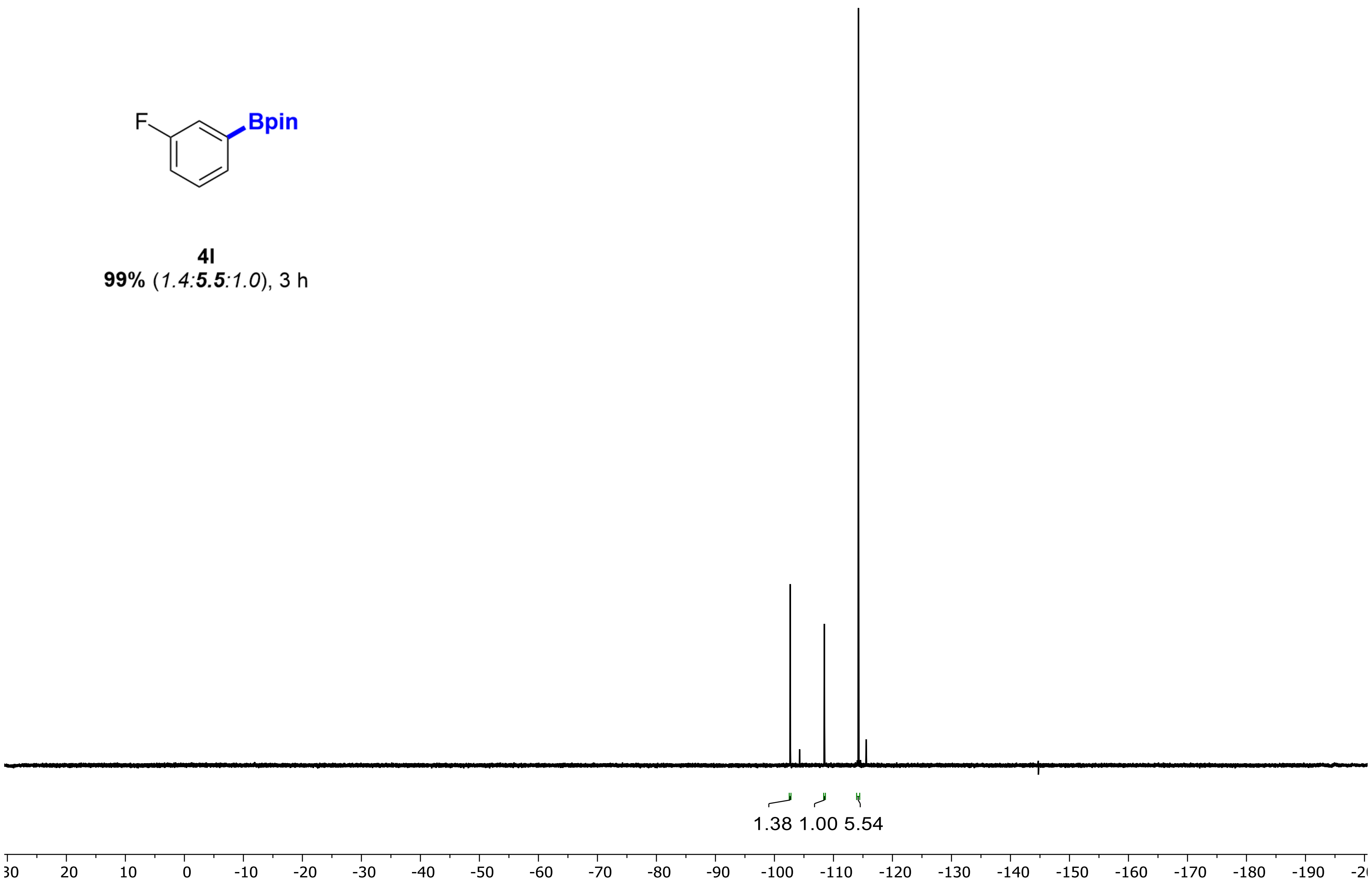

Figure S54. ^{19}F NMR of **4l** (470 MHz, $CDCl_3$)

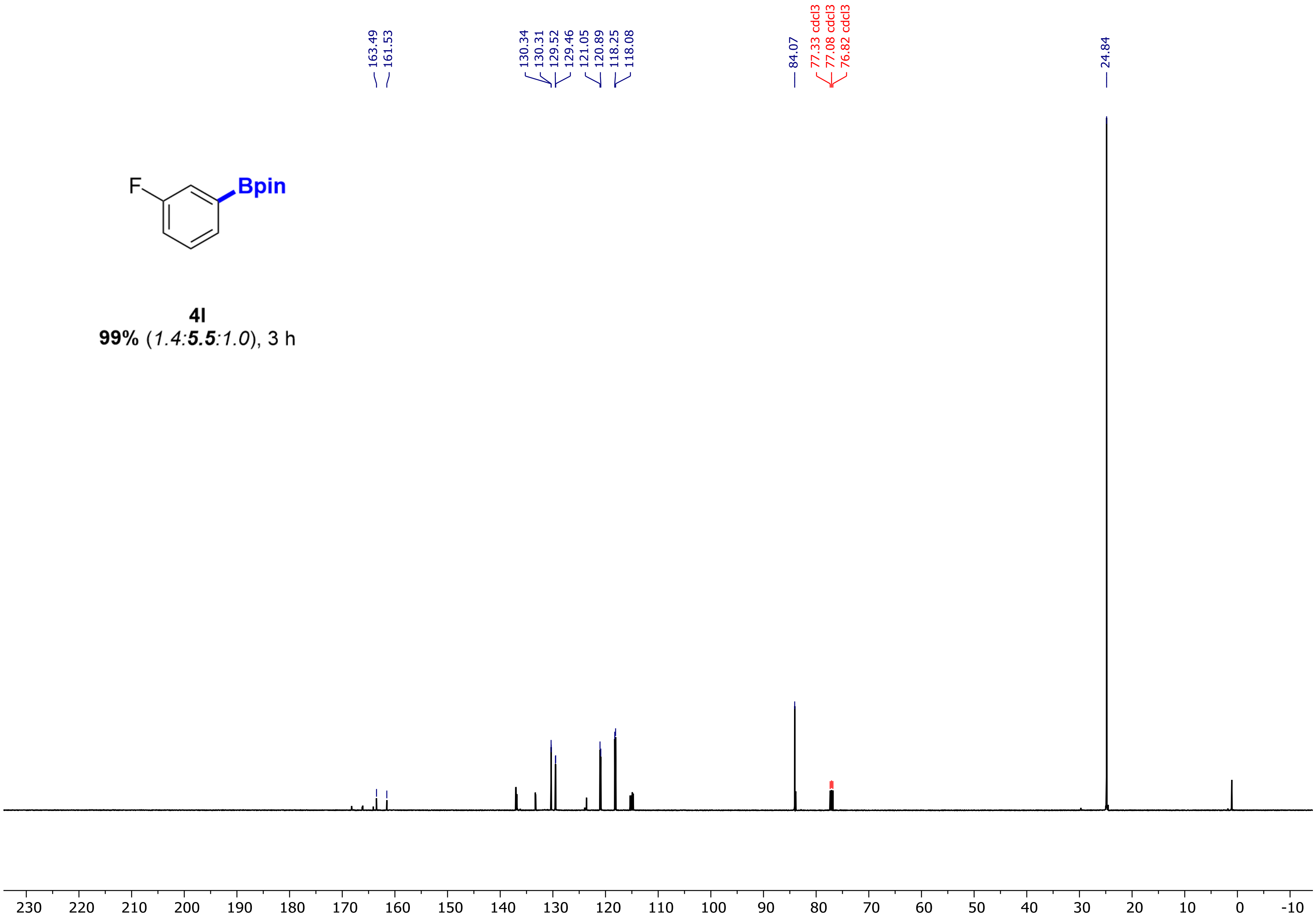

4l
99% (*1.4*:***5.5***:*1.0*), 3 h

Figure S55. ^{13}C NMR of **4l** (126 MHz, $CDCl_3$)

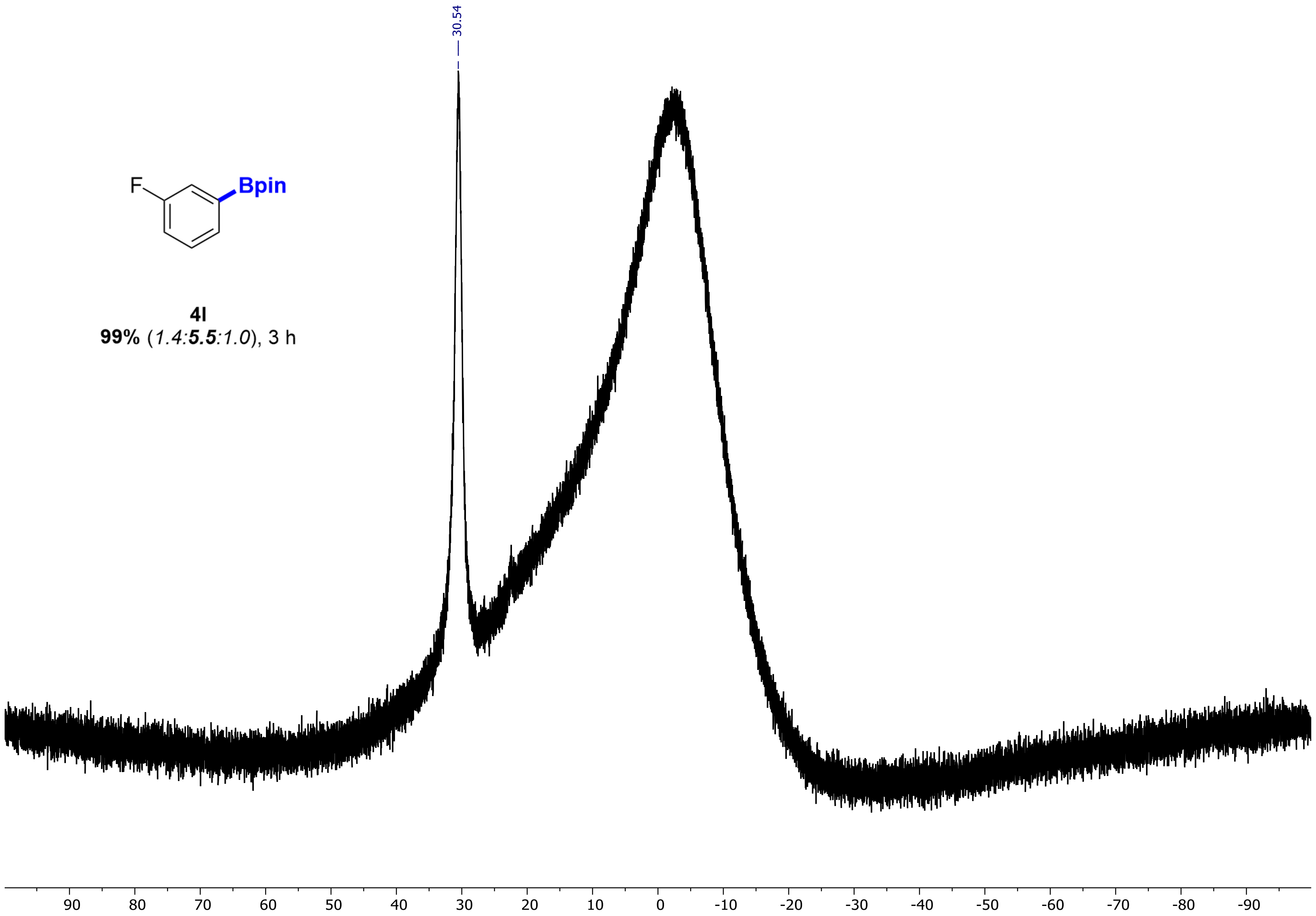

Figure S56. ^{11}B NMR of **4l** (160 MHz, $CDCl_3$)

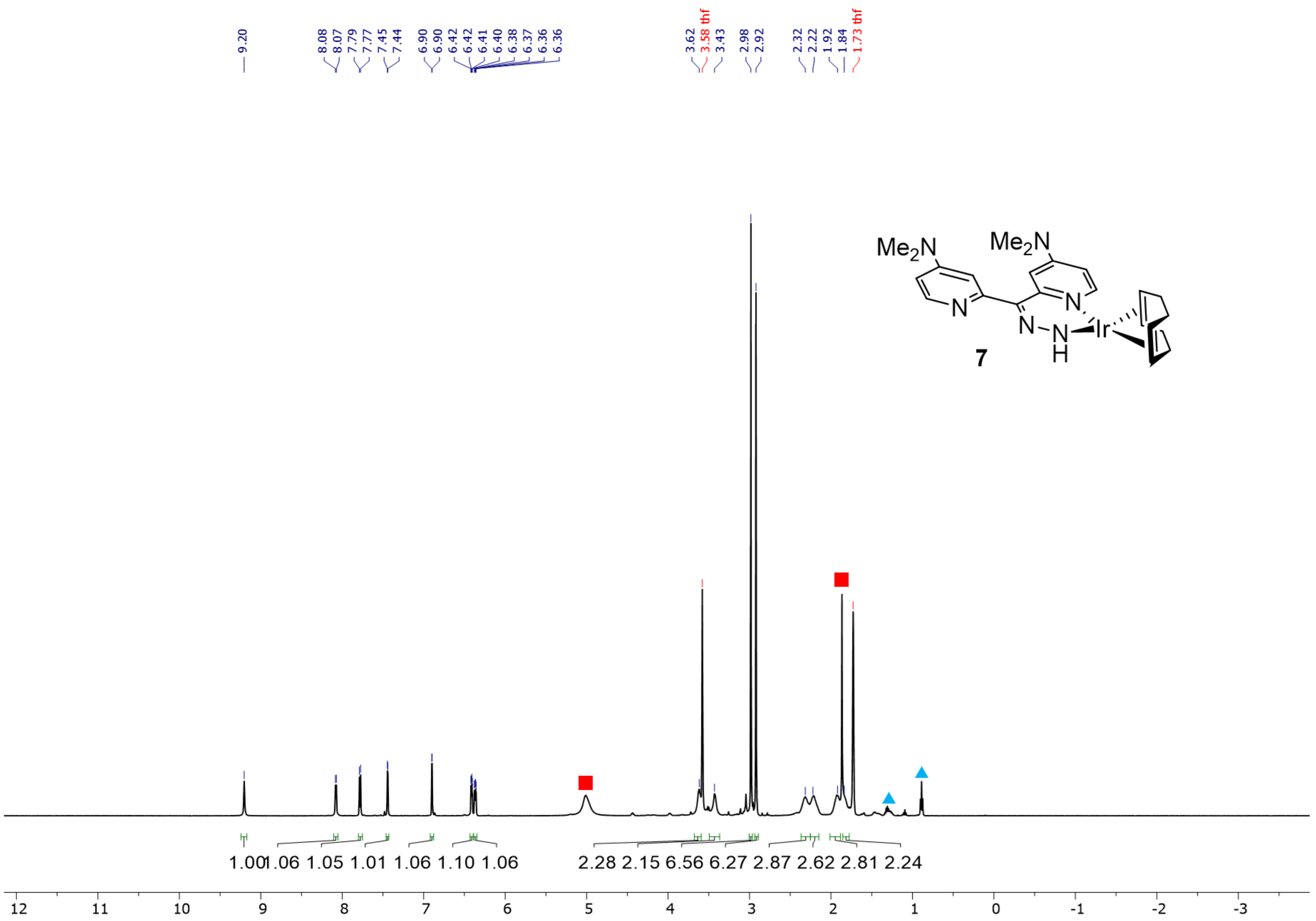

Figure S57. ^{1}H NMR of **7** (500 MHz, THF-d_8). Blue triangles (▲) represent impurities from NMR solvent. Red squares (■) represent impurities found after isolation.

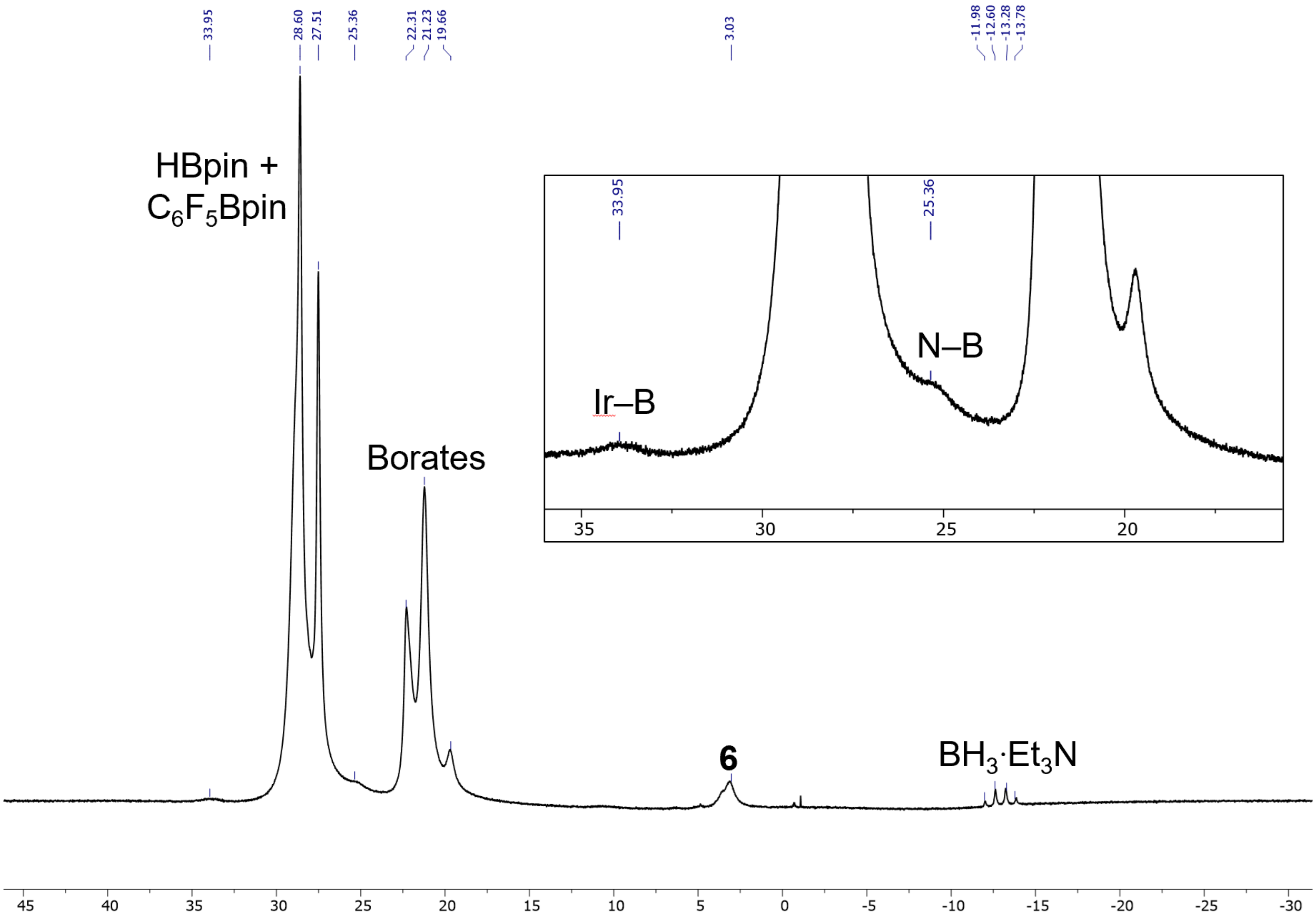

Figure S58. Annotated ^{11}B NMR (500 MHz, THF/THF-d_8) of NMR tube borylation of pentafluorobenzene.
